# Examining two sets of introgression lines across multiple environments reveals background-independent and stably expressed quantitative trait loci of fiber quality in cotton

**DOI:** 10.1007/s00122-020-03578-0

**Published:** 2020-03-17

**Authors:** Yuzhen Shi, Aiying Liu, Junwen Li, Jinfa Zhang, Shaoqi Li, Jinfeng Zhang, Liujun Ma, Rui He, Weiwu Song, Lixue Guo, Quanwei Lu, Xianghui Xiang, Wankui Gong, Juwu Gong, Qun Ge, Haihong Shang, Xiaoying Deng, Jingtao Pan, Youlu Yuan

**Affiliations:** 1 State Key Laboratory of Cotton Biology, Key Laboratory of Biological and Genetic Breeding of Cotton, The Ministry of Agriculture, Institute of Cotton Research, Chinese Academy of Agricultural Sciences, Anyang, 455000 Henan China; 2 Department of Plant and Environmental Sciences, New Mexico State University, Las Cruces, NM 88003 USA

## Abstract

**Key message:**

Background-independent (BI) and stably expressed (SE) quantitative trait loci (QTLs) were identified using two sets of introgression lines across multiple environments. Genetic background more greatly affected fiber quality traits than environmental factors. Sixty-one SE-QTLs, including two BI-QTLs, were novel and 48 SE-QTLs, including seven BI-QTLs, were previously reported.

**Abstract:**

Cotton fiber quality traits are controlled by QTLs and are susceptible to environmental influence. Fiber quality improvement is an essential goal in cotton breeding but is hindered by limited knowledge of the genetic basis of fiber quality traits. In this study, two sets of introgression lines of *Gossypium hirsutum* × *G. barbadense* were used to dissect the QTL stability of three fiber quality traits (fiber length, strength and micronaire) across environments using 551 simple sequence repeat markers selected from our high-density genetic map. A total of 76 and 120 QTLs were detected in the CCRI36 and CCRI45 backgrounds, respectively. Nine BI-QTLs were found, and 78 (41.71%) of the detected QTLs were reported previously. Thirty-nine and 79 QTLs were SE-QTLs in at least two environments in the CCRI36 and CCRI45 backgrounds, respectively. Forty-eight SE-QTLs, including seven BI-QTLs, were confirmed in previous reports, and 61 SE-QTLs, including two BI-QTLs, were considered novel. These results indicate that genetic background more strongly impacts on fiber quality traits than environmental factors. Twenty-three clusters with BI- and/or SE-QTLs were identified, 19 of which harbored favorable alleles from *G. barbadense* for two or three fiber quality traits. This study is the first report using two sets of introgression lines to identify fiber quality QTLs across environments in cotton, providing insights into the effect of genetic backgrounds and environments on the QTL expression of fiber quality and important information for the genetic basis underlying fiber quality traits toward QTL cloning and molecular breeding.

**Electronic supplementary material:**

The online version of this article (10.1007/s00122-020-03578-0) contains supplementary material, which is available to authorized users.

## Introduction

Cotton is an important economic crop worldwide that produces natural fibers used in the textile industry. It is essential that fiber quality is improved in order to keep pace with the development of spinning technology and cotton harvesting mechanization. However, the narrow genetic variation in Upland cotton limits the improvement in cotton varieties (Qin et al. 2008). It has been a long-term challenge for cotton breeders to improve fiber quality and yield to meet the needs of cotton producers and the textile industry. Cotton (*Gossypium* spp.) contains 52 species (Li et al. 2014), including two important cultivated tetraploid species: *G. hirsutum* (Upland cotton), with a high fiber yield, wide adaptability and medium fiber quality, and *G. barbadense* (Sea-Island, Egyptian or Pima cotton), with a low fiber yield, and narrow adaptability but high fiber quality (Lu et al. 2017; Shi et al. 2016).

Therefore, introducing desirable genes from *G. barbadense* into Upland cotton cultivars and mapping the quantitative trait loci (QTLs) for fiber quality traits transferred from *G. barbadense* using introgression lines or chromosome segment substitution lines in the Upland cotton background could facilitate improvements in the fiber quality of Upland cotton.

Fiber length (FL), strength (FS) and micronaire (FM) are the three most important traits for evaluating fiber quality. Fiber quality traits are complex quantitative traits controlled by multiple genes and are susceptible to environmental impacts (Tan et al. 2018). Therefore, the use of traditional breeding methods alone for fiber quality breeding is neither accurate nor efficient. Molecular marker-assisted selection (MAS) is a fast and effective method for improving Upland cotton fiber quality.

In the past 20 years, researchers have identified a large number of QTLs related to fiber quality in *G. barbadense* using interspecific segregating populations of *G. hirsutum***×***G. barbadense* or natural populations of *G. barbadense* (Abdullaev et al. 2017; Said et al. 2015a), but most of the mapping populations are early segregating populations of *G. hirsutum***×***G. barbadense* such as F_2_ populations (Jiang et al. 1998; Kohel et al. 2001; Lin et al. 2005; Mei et al. 2004; Paterson et al. 2003), F_2:3_ populations (He et al. 2007) and early backcross generation populations (BC_1_, BC_2_, BC_2_S_1_) (Lacape et al. 2005; Shi et al. 2015, 2016). A few mapping populations are recombinant inbred line (RIL) populations (Lacape et al. 2009, 2010) or backcross introgression line (BIL) populations (Nie et al. 2015; Yu et al. 2013), as well as natural populations of *G. barbadense* (Abdullaev et al. 2017; Wang et al. 2013a). Due to the complex background of these mapping populations, it is difficult to accurately identify and precisely locate QTLs (Islam et al. 2016). Therefore, most of the QTL mapping results cannot be applied to the genetic improvement in Upland cotton (Sun et al. 2017). Introgression lines (ILs), also known as chromosome segment introgression lines (CSILs) or chromosome segment substitution lines (CSSLs), are constructed by hybridization, backcrossing, self-pollination and MAS. Only the introgressed segment differs between a CSSL and its recipient parent. As the same set of CSSLs has the same or a similar genetic background but differs only in a specific genetic region on one chromosome (thus eliminating the influence of complex genetic backgrounds), CSSLs are ideal materials for studying quantitative traits and QTL mapping in crops. The construction and utilization of CSSLs have been widely reported in tomato, rice, maize and other crops (Balakrishnan et al. 2019; Bouchez et al. 2002; Monforte and Tanksley 2000; Okada et al. 2018; Qi et al. 2013; Qiu et al. 2017). In cotton, chromosome substitution lines (CSLs) were first constructed, in which a pair of chromosomes or chromosome arms of Upland TM-1 (the recipient parent) were replaced by those of 3–79 (the donor parent, *G. barbadense*) (Stelly et al. 2005). CSLs differ from CSSLs or ILs in that a pair of recipient parent chromosomes or a pair of chromosome arms is replaced by a pair from the donor parents in the recipient parent, while CSSLs or ILs contain one or a few chromosome segments from the donor parent in the recipient parent background. Many researchers have evaluated and performed genetic studies on CSLs (Jenkins et al. 2006; Saha et al. 2013; Wu et al. 2006), and CSSLs have been gradually constructed and used. Wang et al. (2008) constructed a set of CSSLs using the standard genetic line TM-1 as the recipient parent and Hai7124 as the donor parent through backcrossing and then used them to map QTLs for fiber quality (Wang et al. 2012). A QTL for FS, *qFS*-*D11*-*1*, was fine mapped using the ILs of TM-1 (*G. hirsutum* L.) × H102 (*G. barbadense* L.) (Su et al. 2013), and a QTL for FL, *qFL*-*chr1*, was fine mapped and analyzed using near-isogenic introgression lines (NIILs) of Upland Tamcot 2111 (*G. hirsutum*, the recurrent parent) **× **Pima S-6 (*G. barbadense*, the donor parent) (Xu et al. 2017). Some QTLs related to fiber quality in *G. barbadense* were identified based on segregating populations of derived progenies of one IL (Chen et al. 2018; Wang et al. 2011, 2016). To date, most of the CSLs, ILs or CSSLs reported in cotton have been detected in the obsolete TM-1 genetic background. Although many QTLs for fiber quality traits have been detected in cotton, few of them have been used in MAS in breeding (Cao et al. 2014). This may be due to inaccurate QTL mapping in populations with complex genetic backgrounds, the use of different environments or the use of different genetic backgrounds in QTL mapping populations and breeding populations. In addition, using the QTLs identified in mapping populations in breeding populations is a challenge. To date, the genetic background effects on QTL expression have not been reported.

To transfer beneficial genes from *G. barbadense* into Upland cotton cultivars, we constructed two sets of CSSLs with two different Upland cotton genetic backgrounds in which Hai1 (*G. barbadense*) was the donor parent and CCRI36 and CCRI45 (*G. hirsutum*) were the recipient parents (Li et al. 2016, 2019a; Lu et al. 2017; Ma et al. 2013; Yang et al. 2009). Some of the CSSLs were genetically evaluated, and some QTLs for yield and fiber quality were identified using secondary segregating populations derived from one, two or four CSSLs as parents (Guo et al. 2015, 2018; Li et al. 2019b; Song et al. 2017; Zhai et al. 2016). With the rapid development of biotechnology, multiple cotton genomes have been sequenced, providing a foundation for further cotton gene identification and molecular breeding at the genome level (Hu et al. 2019; Li et al. 2015; Wang et al. 2019).

In this paper, two sets of CSSLs were evaluated and used to dissect the genetic basis of the stability of cotton fiber quality traits across multiple environments and multiple genetic backgrounds, including the identification of more genetic BI- and/or SE-QTLs for fiber quality traits, thus providing new and important stable QTLs with known genomic segments for fine gene mapping, gene cloning and molecular breeding. To the best of our knowledge, this study represents the first report using two sets of CSSLs with different genetic backgrounds but with the same donor parent to dissect the stability of QTLs affecting fiber quality traits as well as to compare and analyze the influence of genetic backgrounds and environments on the expression of fiber quality QTLs in cotton.

## Materials and methods

### Development of two sets of cotton CSSLs and multi-environment field experiments

Two sets of CSSLs were derived from two interspecific crosses with Hai1 (*G. barbadense*) as the donor parent and CCRI36 or CCRI45 (*G. hirsutum*) as the recipient parent. CCRI45 (also called CCRI221) is a late-maturing Upland cotton (*G. hirsutum*) cultivar, and CCRI36 is an early-maturing Upland cotton cultivar; both cultivars have high yield and were bred by the Institute of Cotton Research (ICR), the Chinese Academy of Agricultural Sciences (CAAS), Anyang, Henan Province. Hai1 is a cultivated line of *G. barbadense* with very high fiber quality.

First, two crosses (resulting in two F_1_ populations) were performed, with Hai1 as the male parent and CCRI36 or CCRI45 (*G. hirsutum*) as the female parent. Subsequently, BC_5_F_3_ populations with the CCRI36 background were obtained by five generations of successive backcrossing (with CCRI36 as the recurrent parent) and two generations of self-pollination and MAS (Li et al. 2016, 2019a). Similarly, BC_4_F_3_ populations with the CCRI45 background were also obtained by four generations of backcrossing (with CCRI45 as the recurrent parent) and two generations of self-pollination and MAS (Yang et al. 2009; Li et al. 2016).

In 2009, 2660 CCRI36 **× **Hai1 BC_5_F_3_ individuals and 2328 CCRI45 **× **Hai1 BC_4_F_3_ individuals were grown in the field in Anyang in Henan Province (Anyang Experiment Farm, ICR, CAAS). In 2010, BC_5_F_3:4_ individuals of CCRI36 **× **Hai1 and BC_4_F_3:4_ individuals of CCRI45 **× **Hai1 were planted in progeny rows. The single-row length was 5 m and the row width was 0.8 m in the 2009 Anyang (09HNA) and 2010 Anyang (10HNA) experiments.

On the basis of the above design, two subpopulations in each genetic background were randomly selected, including 408 CSSLs in the CCRI36 background, named 36Pop, and 332 CSSLs in the CCRI45 background, named 45Pop.

Subsequently, 36Pop was evaluated in a total of five environments as follows. In 2011, individual CSSLs in 36Pop (BC_5_F_3:5_) and their recurrent parent were grown in three environments at three different locations: Anyang in Henan Province (11HNA), Liaoyang in Liaoning Province (11LNL) and Shihezi in Xinjiang Autonomous Region (11XJS). In 2014, the same population and the recurrent parent were further evaluated in the Shihezi experiment farm of ICR of CAAS and the experiment field of cotton research institute of Xinjiang Academy of Agricultural and Reclamation Science in Xinjiang Autonomous Region (14XJS and 14XJN, respectively).

45Pop was evaluated in a total of seven environments as follows. Individual CSSLs in 45Pop (BC_4_F_3:5_) and their recurrent parent were grown in seven environments at four different locations in different years: Anyang in Henan Province in 2011 and 2015 (11HNA and 15HNA, respectively), Korla in Xinjiang Autonomous Region in 2011 (11XJK) and 2014 (14XJK), Alaer in Xinjiang Autonomous Region in 2014 (14XJA) and Zhoukou in Henan Province in 2014 (14HNZ) and 2015 (15HNZ).

A randomized incomplete-block design with two replicates was adopted in all the environments, except in 15AY with one replicate, in which a randomized incomplete-block design with one replicate was adopted. In each environment, the recurrent parent, used as a control, was planted with 19 CSSLs at intervals in each of the environments. Single-row plots with a 5 m length and 0.8 m width were used in 11HNA and 15AY, whereas single-row plots with a 5 m length and 1 m width were used in 14HNZ and 15HNZ. Two-row plots with a 3 m length and 0.4 m width between each row were used in 11LNL, whereas two-narrow row plots with a 3 m length and 0.2 m width between the narrow rows, a plastic membrane cover and a wide/narrow row-spacing pattern (a 0.6 m width between two wide rows) were adopted in 11XJS, 11XJK, 14XJS, 14XJN, 14XJK and 14XJA.

Local field management practices were carried out in each of the environments or locations.

### Evaluation of phenotypic traits

Naturally opened bolls were collected from the BC_5_F_3_ individuals of CCRI36 **× **Hai1 and BC_4_F_3_ individuals of CCRI45 **× **Hai1 in 09AY, and 30 naturally opened bolls (from the middle of plants) were harvested in every plot (row) in the other environments (10HNA, 11HNA, 11LNL, 11XJS, 11XJK, 14XJN, 14XJS, 14XJA, 14XJK, 14HNA, 15HNA and 15HNZ) for testing three important fiber quality parameters of fiber strength (FS, cN/Tex), fiber length (FL, mean upper-half length, mm) and fiber micronaire value (FM, an integrated fiber quality parameter of fineness and maturity, unit) using HFT9000 (Premier Evolvics Pvt. Ltd, India) instruments with HVICC Calibration at the Cotton Quality Supervision, Inspection and Testing Center, Ministry of Agriculture, Anyang, China.

### Molecular markers and genotype detection

Genomic DNA was extracted from young leaves of the CSSLs and their parents using a modified cetyl trimethylammonium bromide (CTAB) method (Paterson et al. 1993). The details for PCR amplification, PCR product electrophoresis and silver staining were the same as in the report of Sun et al. (2012).

Based on the genetic linkage map comprising 2292 marker loci distributed on 26 chromosomes and covering almost the whole cotton AD genome (5115.16 cM) with an average marker interval of 2.23 cM, 551 simple sequence repeat (SSR) markers with an average interval of 10 cM between two markers were selected for the screening of genotypes in two sets of CSSLs (Shi et al. 2015). Chromosome (C) 4 had the least number of markers (11 SSRs), while C11, C19 and C21 had the largest (30 SSRs). The longest genetic distance between two markers was 25.99 cM, and the shortest was 0.45 cM. The details of selected markers and their adjacent markers on the genetic map are in Table S1. The sequences of each primer used in this report can be downloaded at http://www.cottonmarker.org and were synthesized by Bioethics Engineering Co., Ltd (Shanghai).

### Data analysis and QTL mapping

The phenotypic data from the CSSLs with the CCRI36 background in seven environments (09HNA, 10HNA, 11HNA, 11LNL, 11XJS, 14XJN, 14XJS) and with the CCRI45 background in nine environments (09HNA, 10HNA, 11HNA, 11XJK, 14XJA, 14XJK, 14HNZ, 15HNZ, 15HNA) were used for analysis.

Descriptive statistical analysis, correlation analysis and analysis of variance (ANOVA) were performed using SPSS 20.0 software (SPSS, Chicago, IL, America). Genotypic analysis of populations and analysis of chromosome introgressed segments calculations (including background recovery rate of the CSSLs, the number and length of introgressed segments) were performed using GGT 2.0 software developed by van Berloo (http://www.plantbreeding.wur.nl/UK/software_ggt.html) (van Berloo 2008).

QTL mapping was performed with QTL IciMapping (version 4.0), and the RSETP-LRTADD mapping method was applied with a logarithm of odds (LOD) threshold of 2.5 (Li et al. 2007; Wang et al. 2019).

The QTLs were named as follows: (q + trait abbreviation) + chromosome/linkage group + QTL number. QTLs for the same trait in different environments and populations were considered stable when their confidence intervals overlapped (Shi et al. 2015, 2016; Sun et al. 2012).

The resulting linkage map and QTLs were drawn using MapChart ver.2.2 software (Voorrips 2002).

## Results

### Evaluation of CSSLs and fiber quality

The results of the descriptive statistical analysis of each trait in the different populations are shown in Table 1. The average FL, FS and FM values of the recurrent parent were generally consistent with those of the corresponding population in all environments. The FL of the populations in all environments was slightly greater than that of the recurrent parents, except in 14XJN; the FS of the populations in the other environments was slightly higher than that of the recurrent parents, except in 10AY; and the FM value of the populations in all environments was slightly lower than that of the recurrent parents. The average FL, FS and FM values of 36 and 45 recurrent parents were similar in all environments, with medium fiber quality. The descriptions of the statistical analysis of quality for 45Pop in 09HNA, 10HNA, 11HNA and 11XJK follow those reported by Ma et al. (2013).

**Table 1:** Fiber quality in CSSL populations and their recipient parents CCRI36 and CCRI45 in multiple environments

CSSL populations								Recipient parents
Background	Trait	Environment	Mean ± SD	Range	CV (%)	Kurtosis	Skewness	
CCRI36	FL	09HNA	27.96 ± 1.55	23.66–32.19	5.54	− 0.13	− 0.13	27.74
		10HNA	28.8 ± 1.11	25.42–32.25	3.87	0.28	− 0.31	28.56
		11HNA	28.5 ± 0.96	25.41–32.02	3.37	0.43	− 0.02	28.11
		11LNL	29.83 ± 0.94	26.61–33.29	3.14	1.30	0.31	29.64
		11XJS	28.21 ± 0.93	24.6–30.94	3.28	0.71	0.03	27.94
		14XJN	28.83 ± 1.08	25.44–32.78	3.73	0.86	0.27	29.16
		14XJS	28.89 ± 0.97	25.83–33.04	3.37	1.00	0.26	28.80
	FM	09HNA	3.68 ± 0.55	2–5.35	15.03	0.23	− 0.25	4.08
		10HNA	4.21 ± 0.34	3.06–5.42	8.01	1.12	− 0.18	4.28
		11HNA	3.950.26	2.95–4.81	6.47	1.48	− 0.35	4.05
		11LNL	4.04 ± 0.25	3.24–5.03	6.30	0.59	0.10	4.26
		11XJS	4.25 ± 0.28	3.27–5.02	6.67	0.30	− 0.26	4.43
		14XJN	4.12 ± 0.32	3.12–5.02	7.79	0.30	− 0.13	4.19
		14XJS	4.01 ± 0.31	2.85–4.96	7.68	0.44	− 0.16	4.19
	FS	09HNA	27.93 ± 2.82	21.7–36.5	10.09	− 0.60	− 0.04	28.23
		10HNA	29.08 ± 1.34	25.2–32.2	4.61	0.15	− 0.47	29.05
		11HNA	28.7 ± 1.19	25.3–33	4.15	0.30	0.12	28.80
		11LNL	29.88 ± 1.05	26.5–33.5	3.52	0.58	0.10	29.95
		11XJS	27.7 ± 1.05	23.85–30.9	3.78	0.39	0.07	27.79
		14XJN	28.21 ± 1.35	23.65–32.4	4.78	0.51	0.12	29.10
		14XJS	29 ± 1.33	25–32.8	4.60	− 0.02	0.05	29.43
CCRI45	FL	14XJA	29.92 ± 1.04	27.06–33.45	3.48	0.09	0.16	27.71
		14XJK	31.12 ± 1.06	27.99–34.11	3.41	0.20	− 0.17	29.63
		14HNZ	30.75 ± 1.11	27.8–35.19	3.61	0.41	0.12	29.29
		15HNA	28.89 ± 1.43	25.3–33.8	4.95	− 0.11	0.29	28.13
		15HNZ	29.53 ± 1.09	26.5–33.3	3.69	0.62	0.43	27.85
	FM	14XJA	4.13 ± 0.34	3.09–5.13	8.23	0.22	− 0.21	4.20
		14XJK	3.94 ± 0.34	2.92–4.88	8.63	0.02	− 0.05	4.17
		14HNZ	5.02 ± 0.39	3.35–6.15	7.77	1.01	− 0.50	5.22
		15HNA	4.94 ± 0.41	3.5–5.9	8.30	0.19	− 0.40	5.03
		15HNZ	5.29 ± 0.36	4.15–6.2	6.81	0.05	− 0.32	5.53
	FS	14XJA	28.47 ± 1.33	24.4–33.15	4.67	0.59	0.29	26.41
		14XJK	27.3 ± 1.55	22.39–32.05	5.68	0.27	0.06	26.72
		14HNZ	31.49 ± 1.54	27.55–35.65	4.89	− 0.29	0.04	29.32
		15HNA	29.63 ± 2.28	22.9–36.5	7.69	− 0.15	0.03	27.97
		15HNZ	29.97 ± 1.7	25.55–33.7	5.67	− 0.58	− 0.17	27.29

The statistical analysis of the quality descriptions of 45Pop in 09HNA, 10HNA, 11HNA and 11XJK refer to the article reported by Ma et al. (2013)

In all environments, the range and coefficient of variation of each trait in all the populations were large. For the same population in all environments, the variation in FM was the greatest among the three traits, and the variation in FS was greater than that in FL.

These results indicate abundant genetic variation in the CSSL populations produced by advanced backcrossing and continuous self-crossing.

The absolute skewness of all traits in all populations and environments was less than 1, thus following a normal distribution (Table 1, Fig. S1).

The correlation coefficients of each trait between different environments were significant (Table S2), indicating that these materials were stable across multiple environments. Most of the correlation coefficients among environments for FL and FM were larger than 0.5, whereas those for FS were smaller than 0.5, indicating that FS was more greatly affected by the environment than FL and FM.

ANOVA revealed highly significant effects of genotype (*G*), the environment (*E*) and the interaction between genotype and the environment (*G* × *E* interaction) on all three traits in the populations (Table 2). The broad-sense heritability values, calculated by partitioning the variance into genetic and *G* × *E* effects, were above 85% for all three traits.

**Table 2:** Analysis of variation (ANOVA) for fiber quality traits of the two CSSL populations with different genetic backgrounds across multiple environments

Trait	Population	Genotype (*G*)	Environment (*E*)	G * *E*	MSE	Heritability (*h*^2^, %)
FL	36Pop	8.36***	244.65***	0.81***	0.55	90.59
FL	45Pop	12.65***	288.81***	1.22***	0.73	90.68
FM	36Pop	0.74***	18.56***	0.09***	0.05	89.00
FM	45Pop	1.28***	160.94***	0.16***	0.08	88.26
FS	36Pop	12.57***	407.83***	1.96***	1.08	85.44
FS	45Pop	19.61***	980.27***	2.55***	1.45	87.67

*, ** and ***, significant at *P* ≤ 0.05, 0.01 and 0.001, respectively

Through the evaluation and analysis of multiple environments, some of the CSSLs with excellent and stable CSSLs were screened out (Li et al. 2017; Lu et al. 2017). These materials with outstanding fiber quality can be used in cotton breeding and gene identification and cloning.

### Genotype analysis

Figures S2 and S3 show the introgressed Hai1 segments on 26 chromosomes in the 36Pop and 45Pop populations of CSSLs, covering almost the entire genome.

**Fig. 1:**
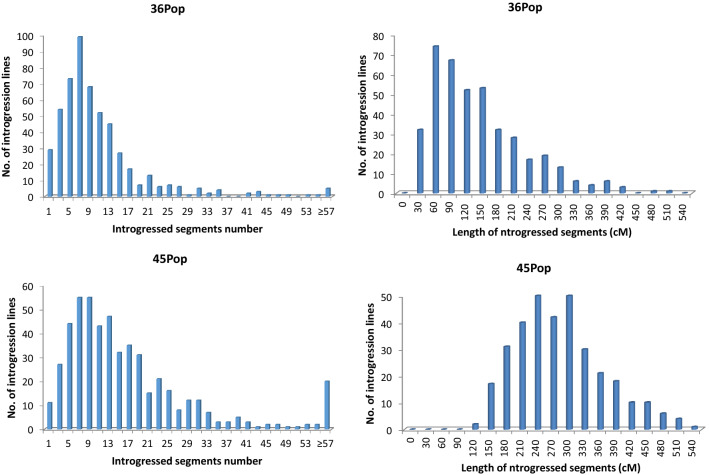
Number and total length of the introgressed Hai1 segments in the two CSSLs populations

The maximum length of the introgressed segments from Hai1 in each individual in the 36Pop population was 488.2 cM; the minimum length was 4.5 cM, and the average length was 125.80 cM. The percent return to the background of the recurrent parent in the population was 90.5–99.8%, with an average of 97.5%. The number of introgressed Hai1 segments was generally 5–20, and the length of the introgressed Hai1 segments was mainly between 30 and 210 cM (Fig. 1). The maximum length of the introgressed segments from Hai1 in each individual in the 45Pop population was 514.1 cM; the minimum length was 94.5 cM, and the average length was 125.80 cM. The percent return to the background of the recurrent parent in the population was 90.5–99.8%, with an average of 97.5%. The number of introgressed Hai1 segments was generally 5–20, and the length of the introgressed Hai1 segments was mainly between 150 and 390 cM (Fig. 1).

### Analysis of QTLs in CSSL populations in the CCRI36 background

**Fig. 2:**
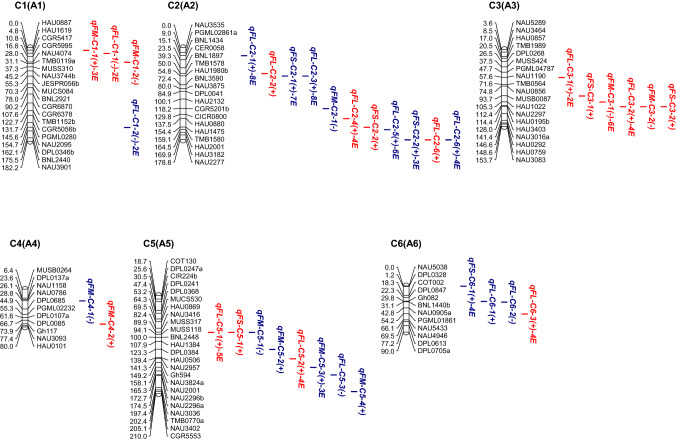

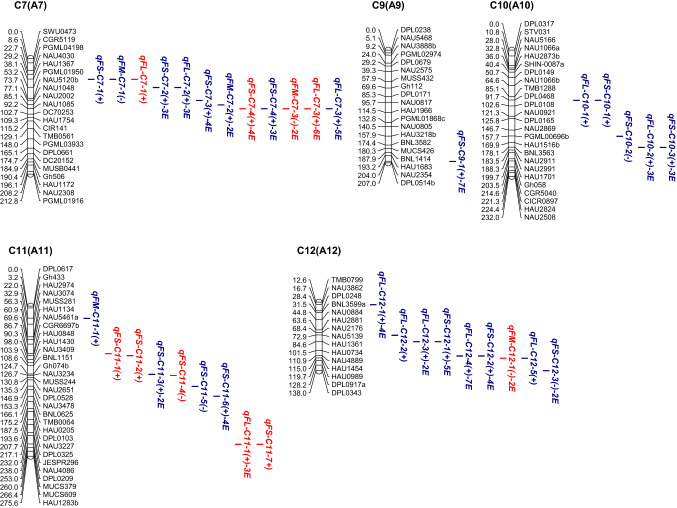

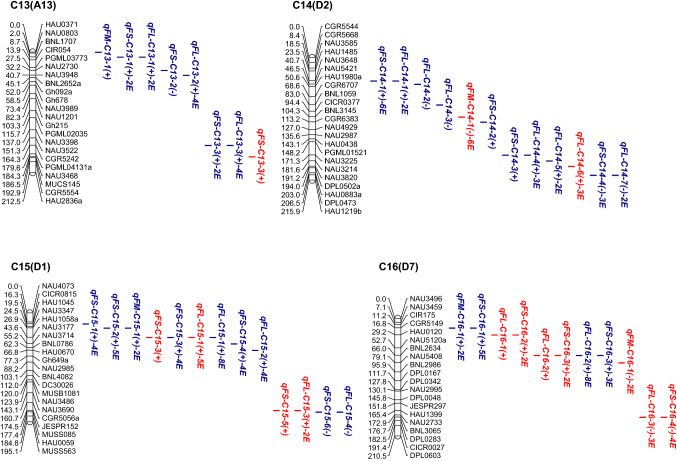

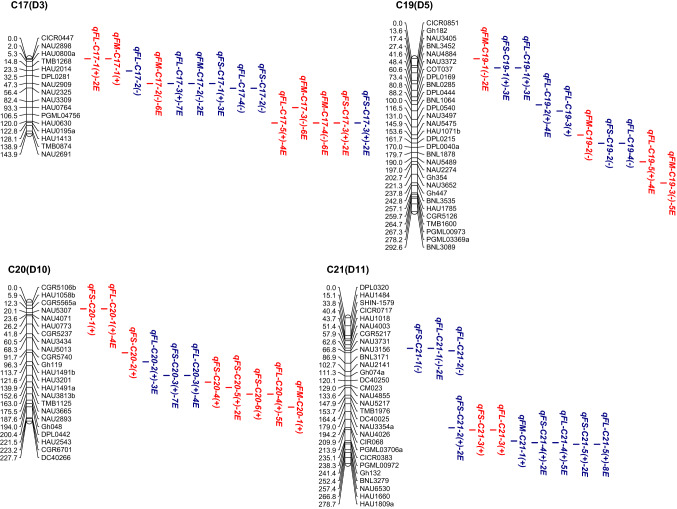

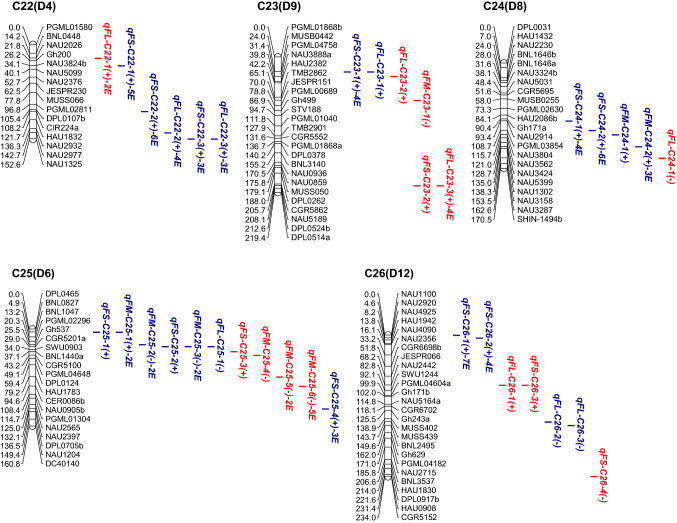
**a**–**e** Chromosomal locations of QTLs for three traits of fiber quality in the CSSLs populations. E, environment; numbers preceding E: the number of environments in which the QTL was detected. Red QTLs, CCRI36-background QTLs; blue QTLs, CCRI45-background QTLs (color figure online)

In the CCRI36 background, a total of 76 QTLs were identified, with a phenotypic variation explained (PVE) of 2.77–13.91% for all three fiber quality traits, 39 of which were detected in at least two environments (Fig. 2, Table S3).

*FL*: A total of 29 QTLs for FL (FL-QTLs) were detected on 19 chromosomes in the CSSL population with the CCRI36 background, but no FL-QTL was detected on C4, C8, C9, C10, C12, C13, C18 and C25. C12 and C16 contained the most FL-QTLs. The PVE by these FL-QTLs ranged from 2.83 to 11.49%; 26 of the 29 FL-QTLs had positive additive effects, and the Hai1 alleles increased FL.

Twenty of QTLs were detected in at least two environments: One QTL (*qFL*-*C7*-*3*) was detected in six environments with a PVE of 3.14–4.32%, three QTLs (*qFL*-*C5*-*1, qFL*-*C15*-*1* and *qFL*-*C20*-*4*) were detected in five environments with a PVE of 3.05–11.49%, eight QTLs (*qFL*-*C2*-*4, qFL*-*C3*-*2, qFL*-*C5*-*2, qFL*-*C6*-*3, qFL*-*C17*-*5, qFL*-*C19*-*5, qFL*-*C20*-*1 and qFL*-*C23*-*3*) were detected in four environments with a PVE of 2.83–10.83%, three QTLs (*qFL*-*C11*-*1, qFL*-*C14*-*6 and qFL*-*C16*-*3*) were detected in three environments with a PVE of 3.10–6.11%, and five QTLs were detected in two environments. Among the 20 FL-QTLs, 18 had positive additive effects, and the Hai1 alleles increased FL.

*FS*: A total of 26 QTLs for FS (FS-QTLs) were detected in CSSL population with the CCRI36 background. They had a PVE of 2.81–10.81%, distributed on 14 chromosomes (C2, C3, C5, C7, C11, C13, C15, C16, C17, C20, C21, C23, C25 and C26), among which C11, C16 and C20 contained the most FS-QTLs (3–5 QTLs). Twenty-three of the 26 FS-QTLs had positive additive effects, and the Hai1 alleles increased FS.

Six of 26 FS-QTLs were detected in at least two environments: Two QTLs (*qFS*-*C7*-*4* and *qFS*-*C16*-*4*) were detected in four environments with a PVE of 3.19–4.21%, and four QTLs were detected in two environments. Five of the six stable QTLs had positive additive effects, and the Hai1 alleles increased FS.

*FM*: A total of 21 QTLs for FM (FM-QTLs) were detected in the CCRI36 background and were distributed on 11 chromosomes (C1, C3, C4, C7, C12, C16, C17, C19, C20, C23 and C25), among which C17, C19 and C25 contained the most FM-QTLs (3–4 QTLs). The PVE by these QTLs ranged from 2.77 to 13.91%. Seventeen of the 21 QTLs had negative additive effects, and the Hai1 alleles decreased FM.

Among the FM-QTLs, 13 were detected in at least two environments: Five QTLs (*qFM*-*C3*-*1*, *qFM*-*C14*-*1*, *qFM*-*C17*-*2*, *qFM*-*C17*-*3* and *qFM*-*C17*-*4*) were detected in six environments with a PVE of 4.11–13.91%, two QTLs (*qFM*-*C19*-*3* and *qFM*-*C25*-*6*) were detected in five environments with a PVE of 2.93–5.12%, one QTLs (*qFM*-*C1*-*1*) was detected in three environments with a PVE of 2.61–3.91% and five QTLs were detected in two environments. Among the 13 stable QTLs, 12 had negative additive effect, and the Hai1 alleles decreased FM.

### Analysis of QTLs in CSSL populations in the CCRI45 background

In the CCRI45 background, a total of 120 QTLs were identified with a PVE ranging from 3.40 to 22.94% for all three fiber quality traits, 79 of which were detected in at least two environments (Fig. 2, Table S3).

*FL*:A total of 49 FL-QTLs were detected on 18 chromosomes in the CSSL populations with the CCRI45 background, while no FL-QTL was detected on C3, C4, C8, C9, C11, C18, C23 and C24. C12 and C14 contained the most FL-QTLs (5–6 QTLs). The PVE by these QTLs ranged from 3.12 to 19.95%; 34 of 49 QTLs had positive additive effects, and the Hai1 alleles increased FL.

Thirty-one of 49 FL-QTLs were detected in at least two environments: Five QTLs (*qFL*-*C2*-*1, qFL*-*C2*-*3, qFL*-*C15*-*1, qFL*-*C16*-*2 and qFL*-*C21*-*5*) were detected in eight environments with a PVE of 3.40–16.95%, five QTLs (*qFL*-*C12*-*4, qFL*-*C17*-*3, qFL*-*C2*-*5, qFL*-*C7*-*3 and qFL*-*C21*-*4*) were detected in 5–7 environments with a PVE of 3.48–15.42%, eight QTLs (*qFL*-*C2*-*6, qFL*-*C12*-*1, qFL*-*C13*-*2, qFL*-*C13*-*3, qFL*-*C15*-*2, qFL*-*C19*-*2, qFL*-*C20*-*3 and qFL*-*C22*-*2*) were detected in four environments with a PVE of 3.44–10.25%, six QTLs (*qFL*-*C7*-*2, qFL*-*C10*-*2, qFL*-*C14*-*4, qFL*-*C19*-*1, qFL*-*C20*-*2* and *qFL*-*C22*-*3*) were detected in three environments with a PVE of 3.41–10.42% and seven QTLs were detected in two environments. Among the 31 stable QTLs, 28 had positive additive effects, and the Hai1 alleles increased FL.

*FS*: A total of 52 FS-QTLs were detected on 20 chromosomes, with a PVE of 3.40–22.94%; and no FS-QTL was detected on C1, C3, C4, C5, C8 and C18. C15, C7, C14 and C21 contained the most FS-QTLs (4–5 QTLs). Forty-three of the 52 QTLs had positive additive effects, and the Hai1 alleles increased FS. Thirty-nine of the 54 FS-QTLs were detected in at least two environments: Four QTLs (*qFS*-*C2*-*1, qFS*-*C9*-*1, qFS*-*C20*-*3* and *qFS*-*C26*-*1*) were detected in seven environments with a PVE of 3.41–15.24%, seven QTLs (*qFS*-*C14*-*1, qFS*-*C22*-*2, qFS*-*C24*-*2, qFS*-*C12*-*1, qFS*-*C15*-*2, qFS*-*C16*-*1* and *qFS*-*C22*-*1*) were detected in 5–6 environments with a PVE of 3.55–15.55%, 10 QTLs (*qFS*-*C6*-*1, qFS*-*C7*-*3, qFS*-*C11*-*6, qFS*-*C12*-*2, qFS*-*C15*-*1, qFS*-*C15*-*3, qFS*-*C15*-*4, qFS*-*C23*-*1, qFS*-*C24*-*1* and *qFS*-*C26*-*2*) were detected in four environments with a PVE of 3.46–12.28%, 10 QTLs (*qFS*-*C2*-*2, qFS*-*C7*-*2, qFS*-*C7*-*4, qFS*-*C10*-*3, qFS*-*C14*-*4, qFS*-*C16*-*3, qFS*-*C17*-*1, qFS*-*C19*-*1, qFS*-*C22*-*3* and *qFS*-*C25*-*4*) were detected in three environments with a PVE of 3.40–22.94% and eight were detected in two environments. Among the 39 stable QTLs, 37 had positive additive effects, and the Hai1 alleles increased FS.

*FM*: A total of 19 FM-QTLs were detected on 12 chromosomes (C2, C4, C5, C7, C11, C13, C15, C16, C17, C21, C24 and C25), in which C5 and C25 contained the most FM-QTLs (3–4 QTLs). The PVE by these QTLs ranged from 3.43 to 8.37%. Twelve of the 19 FM-QTLs had negative additive effects, and the Hai1 alleles decreased FM.

Among these FM-QTLs, nine were detected in at least two environments: Two QTLs (*qFM*-*C5*-*3* and *qFM*-*C24*-*2*) were detected in three environments with a PVE of 3.45–7.66%, and seven QTLs (*qFM*-*C15*-*1*, *qFM*-*C16*-*1*, *qFM*-*C17*-*2*, *qFM*-*C25*-*1*, *qFM*-*C25*-*2* and *qFM*-*C25*-*3*) were detected in two environments with a PVE of 3.43–8.37%. Among the nine stable QTLs, six had positive additive effects, and the Hai1 alleles increased FM.

### QTLs detected in both genetic backgrounds simultaneously

Among the above QTLs, nine background-independent QTLs (BI-QTLs) were simultaneously detected in both backgrounds, including four FL-QTLs, four FS-QTLs and one FM-QTLs (Table 3, Table S3).

**Table 3:** Common QTLs in two independent genetic backgrounds

QTLs	Backgrounds	*C*	Position	Nearest markers	No. of EE	Previous reports
*qFL*-*C2*-*6*(+)	CCRI36	2	178.82	NAU2277	1	
*qFL*-*C2*-*6*(+)	CCRI45	2	178.82	NAU2277	4	
*qFL*-*C7*-*3*(+)	CCRI36	7	92.24	NAU1085	6	Sun et al. (2012), Song et al. (2017), Li et al. (2019b), Jamshed et al. (2016), Deng et al. (2019)
*qFL*-*C7*-*3*(+)	CCRI45	7	92.24	NAU1085	5	Sun et al. (2012), Song et al. (2017), Li et al. (2019b), Jamshed et al. (2016), Deng et al. (2019)
*qFL*-*C15*-*1*(+)	CCRI36	15	43.61	NAU3177	5	
*qFL*-*C15*-*1*(+)	CCRI45	15	43.61	NAU3177	8	
*qFL*-*C16*-*2*(+)	CCRI36	16	65.97	BNL2634	1	Shen et al. (2005), Li et al. (2019b)
*qFL*-*C16*-*2*(+)	CCRI45	16	65.97	BNL2634	8	Shen et al. (2005), Li et al. (2019b)
*qFM*-*C17*-*2*(−)	CCRI36	17	47.26	NAU2909	6	Zhai et al. (2016)
*qFM*-*C17*-*2*(−)	CCRI45	17	47.26	NAU2909	2	Zhai et al. (2016)
*qFS*-*C7*-*4*(+)	CCRI36	7	92.24	NAU1085	4	Sun et al. (2012), Song et al. (2017), Li et al. (2019b), Jamshed et al. (2016)
*qFS*-*C7*-*4*(+)	CCRI45	7	92.24	NAU1085	3	Sun et al. (2012), Song et al. (2017), Li et al. (2019b), Jamshed et al. (2016)
*qFS*-*C15*-*3*(+)	CCRI36	15	43.61	NAU3177	1	Li et al. (2019b)
*qFS*-*C15*-*3*(+)	CCRI45	15	43.61	NAU3177	4	Li et al. (2019b)
*qFS*-*C16*-*3*(+)	CCRI36	16	65.97	BNL2634	2	Shen et al. (2005), Li et al. (2019b)
*qFS*-*C16*-*3*(+)	CCRI45	16	65.97	BNL2634	3	Shen et al. (2005), Li et al. (2019b)
*qFS*-*C17*-*3*(+)	CCRI36	17	122.79	HAU0195a	2	Li et al. (2019b)
*qFS*-*C17*-*3*(+)	CCRI45	17	122.79	HAU0195a	2	Li et al. (2019b)

No. of EE, No. of environments of QTL expression; C, chromosome; (+), positive additive effect indicated that Hai1 alleles increased the phenotypic trait values; (−), negative additive effect indicated that Hai1 alleles decreased the phenotypic trait values

Among the four FL-QTLs, *qFL*-*C7*-*3* near NAU1085 on C7 was simultaneously detected in six environments in the CCRI36 background and in five environments in the CCRI45 background; *qFL*-*C15*-*1* near NAU3177 on C15 was simultaneously detected in five environments in the CCRI36 background and in eight environments in the CCRI45 background; *qFL*-*C16*-*2* near BNL2634 on C16 was simultaneously detected in eight environments in the CCRI45 background and in one environment in the CCRI36 background; and *qFL*-*C2*-*6* near NAU2277 on C2 was simultaneously detected in four environments in the CCRI45 background and in one environment in the CCRI36 background. The Hai1 alleles in the four FL-QTLs all increased FL.

Among the four FS-QTLs, *FS*-*C7*-*4* near NAU1085 on C7 was simultaneously detected in four environments in the CCRI36 background and in three environments in the CCRI45 background; *qFS*-*C16*-*3* near BNL2634 on C16 was simultaneously detected in two environments in the CCRI36 background and in three environments in the CCRI45 background; *qFS*-*C17*-*3* near HAU0195a on C17 was simultaneously detected in two environments of each of both backgrounds; *qFS*-*C15*-*3* near NAU3177 on C15 was simultaneously detected in four environments in the CCRI45 background and in one environment in the CCRI36 background. The Hai1 alleles in the four FS-QTLs increased FS.

Only one FM-QTL (*qFM*-*C17*-*2*) near NAU2909 on C17 was detected in six environments in the CCRI36 background and in two environments in the CCRI45 background.

Therefore, six QTLs (*qFL*-*C7*-*3*, *qFL*-*C15*-*1*, *qFM*-*C17*-*2*, *qFS*-*C7*-*4*, *qFS*-*C16*-*3* and *qFS*-*C17*-*3*) were detected in multiple environments in each of both backgrounds, and three QTLs (*qFL*-*C2*-*6*, *qFL*-*C16*-*2* and *qFS*-*C15*-*3*) were detected in multiple environments in one background and in one environment in another background.

### Fiber quality QTL clusters

The QTL clusters were defined as a QTL-rich region that contained two or more QTLs of various trait types within common confidence region. Some of the QTLs formed clusters, which is a common and previously reported phenomenon (Lacape et al. 2010; Sun et al. 2012; Said et al. 2015a, b; Zhai et al. 2016). A total of 23 QTL clusters were found in this paper, with at least two stable or common QTLs affecting at least two or more different traits. These clusters were distributed on 13 chromosomes (C2, C7, C10, C12, C13, C14, C15, C16, C17, C19, C20, C21 and C22) (Table 4, Table S4).

**Table 4:** QTL clusters for fiber quality traits in CSSLs populations

Cluster	QTL	Genetic backgrounds	*C*	Position	Nearest marker	No. of EE	Previous reports
*BISER*-*C2*-*1*	*qFL*-*C2*-*6*(+)	CCRI36	2	178.82	NAU2277	1	
	*qFL*-*C2*-*6*(+)	CCRI45	2	178.82	NAU2277	4	
	*qFS*-*C2*-*2*(+)	CCRI45	2	178.82	NAU2277	3	
*BISER*-*C7*-*1*	*qFL*-*C7*-*3*(+)	CCRI36	7	92.24	NAU1085	6	Sun et al. (2012), Song et al. (2017), Li et al. (2019b), Jamshed et al. (2016), Deng et al. (2019)
	*qFL*-*C7*-*3*(+)	CCRI45	7	92.24	NAU1085	5	Sun et al. (2012), Song et al. (2017), Li et al. (2019b), Jamshed et al. (2016), Deng et al. (2019)
	*qFM*-*C7*-*3*(−)	CCRI36	7	92.24	NAU1085	2	Sun et al. (2012)
	*qFS*-*C7*-*4*(+)	CCRI36	7	92.24	NAU1085	4	Sun et al. (2012), Song et al. (2017), Li et al. (2019b), Jamshed et al. (2016)
	*qFS*-*C7*-*4*(+)	CCRI45	7	92.24	NAU1085	3	Sun et al. (2012), Song et al. (2017), Li et al. (2019b), Jamshed et al. (2016)
*BISER*-*C15*-*1*	*qFL*-*C15*-*1*(+)	CCRI36	15	43.61	NAU3177	5	
	*qFL*-*C15*-*1*(+)	CCRI45	15	43.61	NAU3177	8	
	*qFS*-*C15*-*3*(+)	CCRI36	15	43.61	NAU3177	1	Li et al. (2019b)
	*qFS*-*C15*-*3*(+)	CCRI45	15	43.61	NAU3177	4	Li et al. (2019b)
*BISER*-*C16*-*1*	*qFL*-*C16*-*2*(+)	CCRI36	16	65.97	BNL2634	1	Shen et al. (2005), Li et al. (2019b)
	*qFL*-*C16*-*2*(+)	CCRI45	16	65.97	BNL2634	8	Shen et al. (2005), Li et al. (2019b)
	*qFS*-*C16*-*3*(+)	CCRI36	16	65.97	BNL2634	2	Shen et al. (2005), Li et al. (2019b)
	*qFS*-*C16*-*3*(+)	CCRI45	16	65.97	BNL2634	3	Shen et al. (2005), Li et al. (2019b)
	*qFL*-*C17*-*3*(+)	CCRI45	17	47.26	NAU2909	7	
*BISER*-*C17*-*1*	*qFM*-*C17*-*2*(−)	CCRI36	17	47.26	NAU2909	6	Zhai et al. (2016)
	*qFM*-*C17*-*2*(−)	CCRI45	17	47.26	NAU2909	2	Zhai et al. (2016)
	*qFS*-*C17*-*1*(+)	CCRI45	17	47.26	NAU2909	3	Ning et al. (2014)
*BISER*-*C17*-*2*	*qFL*-*C17*-*5*(+)	CCRI36	17	122.79	HAU0195a	4	
	*qFM*-*C17*-*4*(−)	CCRI36	17	122.79	HAU0195a	6	Wang et al. (2016)
	*qFS*-*C17*-*3*(+)	CCRI36	17	122.79	HAU0195a	2	Li et al. (2019b)
	*qFS*-*C17*-*3*(+)	CCRI45	17	122.79	HAU0195a	2	Li et al. (2019b)
*SER*-*C2*-*1*	*qFL*-*C2*-*3*(+)	CCRI45	2	54.78	HAU1980b	8	Zhai et al. (2016)
	*qFS*-*C2*-*1*(+)	CCRI45	2	54.78	HAU1980b	7	
*SER*-*C7*-*1*	*qFL*-*C7*-*2*(+)	CCRI45	7	53.19	PGML01950	3	
	*qFS*-*C7*-*2*(+)	CCRI45	7	53.19	PGML01950	3	
*SER*-*C10*-*1*	*qFL*-*C10*-*2*(+)	CCRI45	10	178.11	BNL3563	3	Shi et al. (2019)
	*qFS*-*C10*-*3*(+)	CCRI45	10	178.11	BNL3563	3	
*SER*-*C12*-*1*	*qFL*-*C12*-*3*(+)	CCRI45	12	84.57	HAU1361	2	
	*qFS*-*C12*-*1*(+)	CCRI45	12	84.57	HAU1361	5	Wang et al. (2012), Shi et al. (2019)
*SER*-*C12*-*2*	*qFL*-*C12*-*4*(+)	CCRI45	12	110.86	NAU4889	7	
	*qFS*-*C12*-*2*(+)	CCRI45	12	110.86	NAU4889	4	
*SER*-*C13*-*1*	*qFL*-*C13*-*1*(+)	CCRI45	13	8.68	BNL1707	2	
	*qFS*-*C13*-*1*(+)	CCRI45	13	8.68	BNL1707	2	
*SER*-*C13*-*2*	*qFL*-*C13*-*3*(+)	CCRI45	13	164.25	CGR5242	4	
	*qFS*-*C13*-*3*(+)	CCRI45	13	164.25	CGR5242	2	
*SER*-*C14*-*1*	*qFL*-*C14*-*1*(+)	CCRI45	14	40.73	NAU3648	2	Zhai et al. (2016)
	*qFS*-*C14*-*1*(+)	CCRI45	14	40.73	NAU3648	6	Zhai et al. (2016)
	*qFL*-*C14*-*2*(−)	CCRI45	14	46.54	NAU5421	1	Zhai et al. (2016)
*SER*-*C14*-*2*	*qFL*-*C14*-*7*(−)	CCRI45	14	206.88	HAU1219a	2	
	*qFS*-*C14*-*4*(−)	CCRI45	14	206.88	HAU1219a	3	
*SER*-*C15*-*1*	*qFM*-*C15*-*1*(+)	CCRI45	15	26.90	HAU1058a	2	Chen et al. (2018), Wang et al. (2016)
	*qFS*-*C15*-*2*(+)	CCRI45	15	26.90	HAU1058a	5	Chen et al. (2018)
*SER*-*C16*-*1*	*qFM*-*C16*-*1*(+)	CCRI45	16	16.79	CGR5149	2	
	*qFS*-*C16*-*1*(+)	CCRI45	16	16.79	CGR5149	5	
*SER*-*C16*-*2*	*qFL*-*C16*-*3*(−)	CCRI36	16	176.71	BNL3065	3	Shi et al. (2019)
	*qFS*-*C16*-*4*(−)	CCRI36	16	176.71	BNL3065	4	Shi et al. (2019)
*SER*-*C19*-*1*	*qFL*-*C19*-*1*(+)	CCRI45	19	17.39	NAU3405	3	Wang et al. (2017a)
	*qFS*-*C19*-*1*(+)	CCRI45	19	17.39	NAU3405	3	
*SER*-*C20*-*1*	*qFL*-*C20*-*3*(+)	CCRI45	20	139.90	HAU1491a	4	
	*qFS*-*C20*-*3*(+)	CCRI45	20	139.90	HAU1491a	7	
*SER*-*C21*-*1*	*qFL*-*C21*-*4*(+)	CCRI45	21	238.32	PGML00972	5	
	*qFS*-*C21*-*4*(+)	CCRI45	21	238.32	PGML00972	2	Wang et al. (2016), Shi et al. (2019)
*SER*-*C21*-*2*	*qFL*-*C21*-*5*(+)	CCRI45	21	241.43	Gh132	8	
	*qFS*-*C21*-*5*(+)	CCRI45	21	241.43	Gh132	2	
*SER*-*C22*-*1*	*qFL*-*C22*-*3*(+)	CCRI45	22	152.64	NAU1325	3	
*BISER*-*C2*-*1*	*qFS*-*C22*-*3*(+)	CCRI45	22	152.64	NAU1325	3	

No. of EE, No. of environments of QTL expression; C, chromosome; (+), positive additive effect indicated that Hai1 alleles increased the phenotypic trait values; (−), negative additive effect indicated that Hai1 alleles decreased the phenotypic trait values

BI- and SE-QTL regions (BISERs) are those containing BI- and SE-QTLs affecting two or more different traits. SE-QTL regions (SERs) are those containing at least two SE-QTLs affecting at least two different traits. Twenty-three QTL clusters included six BISERs and 17 SERs. Six BISERs were involved in the control of two or three traits. *BISER*-*C7*-*1* was located near the NAU1085 marker (92.24 cM) on C7, which harbored two BI-QTLs (*qFL*-*C7*-*3* and *qFS*-*C7*-*4* with a positive additive effect) and one SE-QTL (*qFM*-*C7*-*3* with a negative additive effect). *BISER*-*C15*-*1* was located near the NAU3177 marker (43.61 cM) on C15, with two BI-QTLs (*qFL*-*C15*-*1* and *qFS*-*C15*-*3*, with positive additive effects). *BISER*-*C16*-*1* was located near the BNL2634 marker (65.97 cM) on C16, with two BI-QTLs (q*FL*-*C16*-*2* and *qFS*-*C16*-*3*, with positive additive effects). *BISER*-*C17*-*1* was located near the NAU2909 marker (47.26 cM) on C17, containing one BI-QTL (*qFM*-*C17*-*2* with a negative additive effect) and two SE-QTLs (*qFL*-*C17*-*3* and *qFS*-*C17*-*1* with a positive additive effect). *BISER*-*C17*-*2* was located near the HAU0195a marker (122.79 cM) on C17, containing one BI-QTL (*qFS*-*C17*-*3* with a positive additive effect) and two SE-QTLs (*qFL*-*C17*-*5* with a positive additive effect and *qFM*-*C17*-*4* with a negative additive effect). *BISER*-*C2*-*1* was located near the NAU2277 marker (178.82 cM) on C2, which contained one BI-QTL (*qFL*-*C2*-*6* with a positive additive effect) and one SE-QTL (*qFS*-*C2*-*2* with a positive additive effect). The Hai1 alleles in the three BISERs (*BISER*-*C7*-*1*, *BISER*-*C17*-*1* and *BISER*-*C17*-*2*) simultaneously increased FL and FS and decreased FM, and those in the other three BISERs simultaneously increased FL and FS.

Among the 17 SERs, 15 affected both FL and FS. These 15 SERs (*SER*-*C2*-*1*, *SER*-*C7*-*1*, *SER*-*C10*-*1*, *SER*-*C12*-*1*, *SER*-*C12*-*2, SER*-*C13*-*1*, *SER*-*C13*-*2*, *SER*-*C14*-*1, SER*-*C14*-*2, SER*-*C16*-*2, SER*-*C19*-*1, SER*-*C20*-*1, SER*-*C21*-*1, SER*-*C21*-*2* and *SER*-*C22*-*1*) were located near HAU1980b (54.78 cM) on C2; near PGML01950 (53.19 cM) on C7; near BNL3563 (178.11 cM) on C10; near HAU1361 (84.57 cM) and NAU4889 (110.86 cM) on C12; near BNL1707 (8.68 cM) and CGR5242 (164.25 cM) on C13; near NAU3648 (40.73 cM) and HAU1219a (206.88 cM) on C14; near BNL3065 (176.71 cM) on C16; near NAU3405 (17.39 cM) on C19; near HAU1491a (139.9 cM) on C20; near PGML00972 (238.32 cM) and Gh132 (241.43 cM) on C21; and near NAU1325 (152.64 cM) on C22. The Hai1 alleles in two regions (*SER*-*C14*-*2* and *SER*-*C16*-*2*) simultaneously decreased FL and FS, and those in the other 13 SERs simultaneously increased FL and FS. The other two SERs (*SER*-*C15*-*1* and *SER*-*C16*-*1*) affected FS and FM, and they were located near HAU1058a (26.9 cM) on C15 and near CGR5149 (16.79 cM) on C16. The Hai1 alleles in the two regions simultaneously increased FS and FM (Table 4, Table S4).

## Discussion

### Characteristics of the materials used in this study

CSSLs are valuable genetic resources for basic and applied research on the improvement in complex traits (Balakrishnan et al. 2019). The materials used in this paper were CSSLs with the Upland cotton background and one or more introgressed segments from *G. barbadense*. Only the introgressed segments differed between the CSSLs and their recipient parents. A set of CSSLs, which had the same or a similar genetic background and differed only in a specific genetic region, can eliminate the influence of a complex genetic background, making CSSLs ideal materials for researching quantitative trait inheritance and gene identification in crops and advantageous in the identification of QTLs. The CSSLs are similar to their recurrent parents in terms of field-observed phenotypes but with one or more specific traits of *G. barbadense* (Ma et al. 2013; Li et al. 2019b).

In this paper, the CSSLs exhibited high genetic diversity in fiber quality traits (Table 1, Fig. S1). Through multiple environmental evaluation, some stable and high-quality lines were obtained (Li et al. 2017; Lu et al. 2017). These CSSLs enriched our understanding of the genetic basis of traits in Upland cotton and will serve as useful materials for further QTL/gene fine mapping and genetic improvement in fiber quality in breeding.

### QTLs for fiber quality traits

Cotton fiber quality traits are very important traits that are largely affected by both genetic backgrounds and environmental factors. In the present study, a total of 76 QTLs (29 FL-QTLs, 26 FS-QTLs and 21 FM-QTLs) and 120 QTLs (49 FL-QTLs, 52 FS-QTLs and 19 FM-QTLs) were detected in the two sets of CSSLs of the CCRI36 and CCRI45 backgrounds, respectively (Fig. 2, Table S3). Nine QTLs (four FL-QTLs, four FS-QTLs and one FM-QTL) were simultaneously detected in both backgrounds (the CCRI36 background and the CCRI45 background) (Table 3, Table S3). Thus, a total of 187 QTLs were identified in this study, including 74 FL-QTLs, 39 FM-QTLs and 74 FS-QTLs.

By comparison, 36 of the 76 QTLs detected in the CCRI36 background and 49 of the 120 QTLs detected in the CCRI45 background were identical or similar to previously reported QTLs (Table S3) as they shared common markers in the confidence interval on the same chromosome. In the CCRI36 background: 11 FL-QTLs (*qFL*-*C1*-*1*, *qFL*-*C2*-*4*, *qFL*-*C5*-*2*, *qFL*-*C7*-*3*, *qFL*-*C16*-*2, qFL*-*C16*-*3, qFL*-*C19*-*5, qFL*-*C20*-*1, qFL*-*C20*-*4, qFL*-*C21*-*3* and *qFL*-*C22*-*1*), 12 FM-QTLs (*qFM*-*C1*-*1*, *qFM*-*C1*-*2*, *qFM*-*C3*-*1*, *qFM*-*C3*-*2*, *qFM*-*C7*-*3, qFM*-*C12*-*1, qFM*-*C14*-*1, qFM*-*C16*-*1, qFM*-*C17*-*2, qFM*-*C17*-*4, qFM*-*C20*-*1* and *qFM*-*C25*-*5*) and 13 FS-QTLs (*qFS*-*C7*-*4, qFS*-*C11*-*1, qFS*-*C13*-*3, qFS*-*C15*-*3, qFS*-*C15*-*5, qFS*-*C16*-*3, qFS*-*C16*-*4, qFS*-*C17*-*3, qFS*-*C20*-*1, qFS*-*C20*-*2, qFS*-*C20*-*4, qFS*-*C20*-*5* and *qFS*-*C25*-*3*) were previously reported (Deng et al. 2019; Fang et al. 2014; Guo et al. 2018; Jamshed et al. 2016; Li et al. 2019b; Ma et al. 2017; Said et al. 2015b; Shao et al. 2014; Shen et al. 2005; Shi et al. 2019; Song et al. 2017; Sun et al. 2012; Wang et al. 2016, 2017a, b; Yang et al. 2015; Zhai et al. 2016; Zhang et al. 2012, 2016). In the CCRI 45 background: 16 FL-QTLs (*qFL*-*C2*-*1, qFL*-*C2*-*3, qFL*-*C7*-*3, qFL*-*C10*-*1, qFL*-*C10*-*2, qFL*-*C14*-*1, qFL*-*C14*-*2, qFL*-*C14*-*3, qFL*-*C14*-*5, qFL*-*C15*-*4, qFL*-*C16*-*2, qFL*-*C17*-*4, qFL*-*C19*-*1, qFL*-*C19*-*2, qFL*-*C25*-*1* and *qFL*-*C26*-*3*) were previously reported, 10 FM-QTLs (*qFM*-*C5*-*2, qFM*-*C5*-*3, qFM*-*C7*-*1, qFM*-*C7*-*2, qFM*-*C15*-*1, qFM*-*C17*-*2, qFM*-*C24*-*2, qFM*-*C25*-*1, qFM*-*C25*-*2* and *qFM*-*C25*-*3*) and 23 FS-QTLs (*qFS*-*C7*-*3, qFS*-*C7*-*4, qFS*-*C9*-*1,qFS*-*C10*-*1, qFS*-*C10*-*2, qFS*-*C11*-*5, qFS*-*C11*-*6, qFS*-*C12*-*1, qFS*-*C14*-*1, qFS*-*C15*-*2, qFS*-*C15*-*3, qFS*-*C15*-*6, qFS*-*C16*-*3, qFS*-*C17*-*1, qFS*-*C17*-*3, qFS*-*C19*-*2, qFS*-*C21*-*1, qFS*-*C21*-*2, qFS*-*C21*-*4, qFS*-*C22*-*1, qFS*-*C24*-*1, qFS*-*C25*-*1* and *qFS*-*C25*-*2*) were previously reported (Chen et al. 2018; Deng et al. 2019; Fang et al. 2014; Guo et al. 2015; He et al. 2007; Huang et al. 2018; Jamshed et al. 2016; Li et al. 2019b; Lin et al. 2005; Ning et al. 2014; Said et al. 2015b; Shao et al. 2014; Shen et al. 2005; Shi et al. 2019; Song et al. 2017; Sun et al. 2012; Tang et al. 2015; Wang et al. 2011, 2012, 2015, 2016; Zhai et al. 2016; Zhang et al. 2012). Notably, seven of QTLs (*qFL*-*C7*-*3, qFL*-*C16*-*2, qFM*-*C17*-*2, qFS*-*C7*-*4, qFS*-*C15*-*3, qFS*-*C16*-*3* and *qFS*-*C17*-*3*) were BI-QTLs. Therefore, 77 (41.17%) of the QTLs detected in this study were previously reported, and the other 110 QTLs were considered novel.

It is worth mentioning that 49 of the QTLs were confirmed in our earlier-generation interspecific backcross populations (Shi et al. 2019) and in our secondary segregating populations constructed using CSSLs (Li et al. 2019b; Song et al. 2017; Zhai et al. 2016; Guo et al. 2015, 2018). These QTLs included 14 QTLs (*qFL*-*C2*-*4, qFL*-*C10*-*2, qFL*-*C16*-*3, qFL*-*C20*-*1, qFM*-*C1*-*2, qFM*-*C24*-*2, qFS*-*C11*-*1, qFS*-*C11*-*6, qFS*-*C12*-*1, qFS*-*C13*-*3, qFS*-*C16*-*4, qFS*-*C20*-*1, qFS*-*C21*-*1* and *qFS*-*C21*-*4*) confirmed in our earlier-generation interspecific backcross populations (Shi et al. 2019), 19 QTLs (*qFL*-*C7*-*3, qFL*-*C10*-*1, qFL*-*C15*-*4, qFL*-*C16*-*2, qFL*-*C25*-*1, qFM*-*C7*-*2, qFM*-*C16*-*1, qFM*-*C25*-*2, qFM*-*C25*-*3, qFM*-*C25*-*5, qFS*-*C7*-*3, qFS*-*C7*-*4, qFS*-*C10*-*1, qFS*-*C15*-*3, qFS*-*C15*-*6, qFS*-*C16*-*3, qFS*-*C17*-*3, qFS*-*C24*-*1* and *qFS*-*C25*-*2*) reported by Li et al. (2019b), seven QTLs (*qFL*-*C7*-*3, qFL*-*C20*-*4, qFL*-*C22*-*1, qFS*-*C7*-*4, qFS*-*C20*-*2, qFS*-*C20*-*4* and *qFS*-*C20*-*5*) reported by Song et al. (2017), 10 QTLs (*qFL*-*C2*-*3, qFL*-*C5*-*2, qFL*-*C14*-*1, qFL*-*C14*-*2, qFL*-*C20*-*1, qFM*-*C17*-*2, qFS*-*C10*-*2, qFS*-*C11*-*1, qFS*-*C14*-*1* and *qFS*-*C20*-*1*) reported by Zhai et al. (2016), two QTLs (*qFS*-*C15*-*5* and *qFS*-*C22*-*1*) reported by Guo et al. (2018) and two QTLs (*qFS-C19-2* and *qFS-C21-2*) reported by Guo et al. (2015). Of them, seven (*qFL*-*C7*-*3, qFL*-*C16*-*2, qFM*-*C17*-*2, qFS*-*C7*-*4, qFS*-*C15*-*3, qFS*-*C16*-*3* and *qFS*-*C17*-*3*) were BI-QTLs (Table S3).

In the CCRI36 background, a total of 39 SE-QTLs (20 FL-QTLs, six FS-QTLs and 13 FM-QTLs) were stably expressed in at least two environments, 23 of which were previously reported, including nine FL-QTLs (*qFL*-*C1*-*1, qFL*-*C2*-*4, qFL*-*C5*-*2, qFL*-*C7*-*3, qFL*-*C16*-*3, qFL*-*C19*-*5, qFL*-*C20*-*1, qFL*-*C20*-*4* and *qFL*-*C22*-*1*), nine FM-QTLs (*qFM*-*C1*-*1, qFM*-*C3*-*1, qFM*-*C7*-*3, qFM*-*C12*-*1, qFM*-*C14*-*1, qFM*-*C16*-*1, qFM*-*C17*-*2, qFM*-*C17*-*4* and *qFM*-*C25*-*5*) and five FS-QTLs (*qFS*-*C7*-*4, qFS*-*C16*-*3, qFS*-*C16*-*4, qFS*-*C17*-*3* and *qFS*-*C20*-*5*) (Table S3). In the CCRI45 background, a total of 79 SE-QTLs (31 FL-QTLs, 39 FS-QTLs and nine FM-QTLs) were stably expressed in multiple environments, 32 of which were previously reported, including nine FL-QTLs (*qFL*-*C2*-*1, qFL*-*C2*-*3, qFL*-*C7*-*3, qFL*-*C10*-*2, qFL*-*C14*-*1, qFL*-*C14*-*5, qFL*-*C16*-*2, qFL*-*C19*-*1* and *qFL*-*C19*-*2*), eight FM-QTLs (*qFM*-*C5*-*3, qFM*-*C7*-*2, qFM*-*C15*-*1, qFM*-*C17*-*2, qFM*-*C24*-*2, qFM*-*C25*-*1, qFM*-*C25*-*2* and *qFM*-*C25*-*3*) and 15 FS-QTLs (*qFS*-*C7*-*3, qFS*-*C7*-*4, qFS*-*C9*-*1, qFS*-*C11*-*6, qFS*-*C12*-*1, qFS*-*C14*-*1, qFS*-*C15*-*2, qFS*-*C15*-*3, qFS*-*C16*-*3, qFS*-*C17*-*1, qFS*-*C17*-*3, qFS*-*C21*-*2, qFS*-*C21*-*4, qFS*-*C22*-*1* and *qFS*-*C24*-*1*) (Table S3). Among them, seven QTLs (*qFL*-*C7*-*3, qFL*-*C16*-*2, qFM*-*C17*-*2, qFS*-*C7*-*4, qFS*-*C15*-*3, qFS*-*C16*-*3* and *qFS*-*C17*-*3*) were BI-QTLs and stably expressed in multiple environments of each of both backgrounds (Table 3, Table S3). Therefore, among the 109 SE-QTLs, 48 were confirmed in previous reports and 61 were considered novel. These QTLs are likely to exhibit genetic stability and warrant further clarification by QTL fine mapping and cloning to better understand the genetics and molecular mechanisms underlying fiber development.

In the present study, more QTLs were located on the D subgenome than the A subgenome (29 on the A subgenome and 47 on the D subgenome in the CCRI36 background, and 50 on the A subgenome and 70 on the D subgenome in the CCRI45 background), which was consistent with most previous reports (Fang et al. 2014; Jiang et al. 1998; Lacape et al. 2010; Paterson et al. 2003; Said et al. 2013; Yang et al. 2015).

### Effects of genetic backgrounds and environments on the expression of QTLs

Fiber quality traits in cotton are complex quantitative traits. One of the difficulties in improving complex traits is the environmental sensitivity of the identified QTLs. The percentages of SE-QTLs for the three traits (FL, FS and FM) were 60.00% and 49.00% in previous papers reported by Sun et al. (2012) and Jamshed et al. (2016), respectively. In the present paper, the overall percentage of SE-QTLs was 62.50%. These results were consistent with the results of previous reports (Sun et al. 2012; Jamshed et al. 2016), indicating that environmental factors have a large influence on fiber quality traits (Tan et al. 2018).

To date, there has been no report on the effect of genetic background on the QTL expression of fiber quality in cotton. In the present study, among the 187 QTLs detected overall, only nine (4.81%) were BI-QTLs, which indicated that genetic background has a strong influence on fiber quality traits in cotton. In rice, some studies show that the expressions of the QTLs for complex traits are strongly affected by genetic background (Qiu et al. 2017; Wan et al. 2005; Zhao et al. 2016; Zheng et al. 2011). The consistency of the QTLs among different genetic backgrounds in rice is relatively low for complex traits, such as appearance quality (14.5%) (Qiu et al. 2017), salt tolerance (15.4%) (Cheng et al. 2012) and drought tolerance (17.9%) (Wang et al. 2013b). Our results are consistent with these reports on other traits in rice.

Comparatively, the percentage of BI-QTLs was much lower than that of SE-QTLs in this study, indicating that genetic background has a stronger impact than the environment on fiber quality. For this reason, breeders should pay much attention to the effects of different environments as well as different genetic backgrounds when QTL information is used in molecular breeding for fiber quality traits.

### Important QTL regions for fiber quality improvement

In this paper, we detected a total of 23 QTL clusters, including six BISERs and 17 SERs, each with at least two SE- or BI-QTLs affecting two or more different fiber quality traits (Table 4, Table S4).

Six BISERs harbored nine BI-QTLs and 15 SE-QTLs, in which three QTLs (*qFL*-*C7*-*3, qFS*-*C7*-*4* and *qFM*-*C7*-*3*) in *BISER*-*C7*-*1*, two QTLs (*qFL*-*C16*-*2* and *qFS*-*C16*-*3*) in *BISER*-*C16*-*1*, two QTLs (*qFM*-*C17*-*2* and *qFS*-*C17*-*1*) in *BISER*-*C17*-*1* and two QTLs (*qFM*-*C17*-*4* and *qFS*-*C17*-*3*) in *BISER*-*C17*-*2* were previously reported (Table 4, Table S4). Therefore, two BISERs (*BISER*-*C2*-*1* and *BISER*-*C15*-*1*) were novel. Seventeen SERs harbored 35 SE-QTLs, 12 of which were previously reported. Two QTLs in each of three SERs (*SER*-*C14*-*1, SER*-*C15*-*1* and *SER*-*C16*-*2*) had been previously reported, whereas the other 14 SERs were novel.

These QTL regions are extremely important determinants of fiber quality traits. These stable or consistent QTL regions provide important resources for QTL fine mapping, gene cloning, MAS and pyramiding in cotton breeding.

Among the 23 clusters, 21 clusters harbored SE-QTLs or BI-QTLs for FS and FL, all with a positive correlation between FS-QTLs and FL-QTLs driven by the same direction of additive effects in each cluster, which explained the significant positive correlation between the two traits in the different populations. Five clusters harbored SE-QTLs or BI-QTLs for FS and FM, three of which (*BISER*-*C7*-*1, BISER*-*C17*-*1* and *BISER*-*C17*-*2*) were negatively correlated between FS-QTLs and FM-QTLs. No QTL clusters with at least two SE- or BI-QTLs for FL and FM were detected in this study.

Nineteen of the 23 QTL clusters (excluding *SER*-*C15*-*1, SER*-*C16*-*1, SER*-*C14*-*2* and *SER*-*C16*-*2*) contained favorable alleles from the introgression segment of Hai1 that could be used for the improvement in fiber quality traits. These QTL clusters are very important and noteworthy, especially the six BISERs with not only SE-QTLs but also BI-QTLs affecting two–three fiber quality traits. Therefore, the application or pyramiding of favorable alleles in the QTLs in the six BISERs from the introgression segment of Hai1 has the potential to greatly improve fiber quality traits in cotton varieties by MAS.

We identified some lines with introgression segments that could increase fiber length and fiber strength and decrease micronaire. For example, MBI9915 was the introgression line of the CCRI36 genetic background with excellent fiber quality, containing two BI-QTLs (*qFL*-*C7*-*3* and *qFS*-*C7*-*4*) with positive additive effects (Song et al. 2017), and MBI7561 was the introgression line of CCRI45 genetic background with excellent fiber quality, containing five BI-QTLs (*qFS*-*C7*-*4, qFS*-*C15*-*3, qFL*-*C16*-*2, qFS*-*C16*-*3* and *qFS*-*C17*-*3*) with positive additive effects (Li et al. 2019b). These provide a material foundation for further gene cloning and fiber quality improvement.

## Conclusion

This study represents the first report using two sets of CSSLs with different genetic backgrounds but with the same donor parent to dissect the stability of QTLs of fiber quality traits across multiple environments in cotton. A total of 76 and 120 QTLs were identified in the CSSLs with the CCRI36 and CCRI45 backgrounds, respectively. Among them, nine BI-QTLs were found, and 78 (41.71%) of the detected QTLs were reported previously. Thirty-nine and 79 were SE-QTLs in at least two environments in the CCRI36 and CCRI45 backgrounds, respectively. Forty-eight SE-QTLs, including seven BI-QTLs, were confirmed in previous reports and 61 SE-QTLs, including two BI-QTLs, were considered novel. Twenty-three clusters with BI- and/or SE-QTLs were identified, 19 of which harbored favorable alleles from *G. barbadense* for two or three fiber quality traits. In summary, these results revealed the BI- and/or SE-QTL regions, indicated that genetic background has a stronger effect on fiber quality traits than environmental factors and provided insights into the effects of genetic background and environment on the expression of fiber quality QTLs in cotton. This study provides valuable information and new stable QTL regions for further QTL cloning and improvement in fiber quality by MAS in cotton breeding.

## Electronic supplementary material

Below is the link to the electronic supplementary material.

**Fig. S1** Frequency distribution of fiber quality traits in the two CSSL populations. (PDF 393 kb)

**Fig. S2** Introgressed Hai1 chromosome segments in the 36Pop population of CSSLs with the CCRI36 genetic background. (PDF 905 kb)

**Fig. S3** Introgressed Hai1 chromosome segments in the 45Pop population of CSSLs with the CCRI45 genetic background. (PDF 758 kb)

**Table S1** Details of selected markers from the genetic linkage map of the CCRI36 × Hai1 BC_1_F_1_ population. (PDF 754 kb)

**Table S2** Correlation analysis of three traits in two populations of two sets of CSSLs with different genetic backgrounds across multiple environments. (PDF 55 kb)

Table S3 Details of all QTLs for three traits of fiber quality in CSSL populations. (PDF 468 kb)

**Table S4** Details of QTL clusters for fiber quality in CSSL populations. (PDF 171 kb)

## References

[CR1] Abdullaev AA, Salakhutdinov IB, Egamberdiev SS, Khurshut EE, Rizaeva SM, Ulloa M, Abdurakhmonov IY (2017). Genetic diversity, linkage disequilibrium, and association mapping analyses of *Gossypium barbadense* L. germplasm. PLoS ONE.

[CR2] Balakrishnan D, Surapaneni M, Mesapogu S, Neelamraju S (2019). Development and use of chromosome segment substitution lines as a genetic resource for crop improvement. Theor Appl Genet.

[CR3] Bouchez A, Hospital F, Causse M, Gallais A, Charcosset A (2002). Marker-assisted introgression of favorable alleles at quantitative trait loci between maize elite lines. Genetics.

[CR4] Cao Z, Wang P, Zhu X, Chen H, Zhang T (2014). SSR marker-assisted improvement of fiber qualities in *Gossypium hirsutum* using *G. barbadense* introgression lines. Theor Appl Genet.

[CR5] Chen Y, Liu G, Ma H, Song Z, Zhang C, Zhang J, Zhang J, Wang F, Zhang J (2018). Identification of introgressed alleles conferring high fiber quality derived from *Gossypium barbadense* L. in secondary mapping populations of *G. hirsutum* L. Front Plant Sci.

[CR6] Cheng L, Wang Y, Meng L, Hu X, Cui Y, Sun Y, Zhu L, Ali J, Xu J, Li Z (2012). Identification of salt-tolerant QTLs with strong genetic background effect using two sets of reciprocal introgression lines in rice. Genome.

[CR7] Deng X, Gong J, Liu A, Shi Y, Gong W, Ge Q, Li J, Shang H, Wu Y, Yuan Y (2019). QTL mapping for fiber quality and yield-related traits across multiple generations in segregating population of CCRI 70. J Cotton Res.

[CR8] Fang DD, Jenkins JN, Deng DD, McCarty JC, Li P, Wu JX (2014). Quantitative trait loci analysis of fiber quality traits using a random-mated recombinant inbred population in Upland cotton (*Gossypium hirsutum* L.). BMC Genom.

[CR9] Guo L, Shi Y, Li J, Gong J, Liu A, Shang H, Gong W, Chen T, Qun Ge, Sun J, Yuan Y (2015). Mapping QTL of fiber yield and quality traits in F_2_ populations of chromosome segment substitution lines from *Gossypium hirsutum *× *Gossypium barbadense*. Cotton Sci.

[CR10] Guo LX, Shi YZ, Gong JW, Liu AY, Tan YN, Gong WK, Li JW, Chen TT, Shang HH, Ge Q, Lu QW, Sun J, Yuan YL (2018). Genetic analysis of the fiber quality and yield traits in *G*-*hirsutum* background using chromosome segments substitution lines (CSSLs) from *Gossypium barbadense*. Euphytica.

[CR11] He DH, Lin ZX, Zhang XL, Nie YC, Guo XP, Zhang YX, Li W (2007). QTL mapping for economic traits based on a dense genetic map of cotton with PCR-based markers using the interspecific cross of *Gossypium hirsutum* × *Gossypium barbadense*. Euphytica.

[CR12] Hu Y, Chen J, Fang L (2019). *Gossypium barbadense* and *Gossypium hirsutum* genomes provide insights into the origin and evolution of allotetraploid cotton. Nat Genet.

[CR13] Huang C, Shen C, Wen T, Gao B, Zhu Li X, Ahmed MM, Li D, Lin Z (2018). SSR-based association mapping of fiber quality in upland cotton using an eight-way MAGIC population. Mol Genet Genom.

[CR14] Islam MS, Fang DD, Thyssen GN, Delhom CD, Liu YL, Kim HJ (2016). Comparative fiber property and transcriptome analyses reveal key genes potentially related to high fiber strength in cotton (*Gossypium hirsutum* L.) line MD52ne. BMC Plant Biol.

[CR15] Jamshed M, Jia F, Gong J (2016). Identification of stable quantitative trait loci (QTLs) for fiber quality traits across multiple environments in *Gossypium hirsutum* recombinant inbred line population. BMC Genom.

[CR16] Jenkins JN, Wu JX, McCarty JC, Saha S, Gutierrez O, Hayes R, Stelly DM (2006). Genetic effects of thirteen *Gossypium barbadense* L. chromosome substitution lines in topcrosses with upland cotton cultivars: I.Yield and yield components. Crop Sci.

[CR17] Jiang C, Wright RJ, El-Zik KM, Paterson AH (1998). Polyploid formation created unique avenues for response to selection in Gossypium (cotton). Proc Natl Acad Sci USA.

[CR18] Kohel RJ, Yu J, Park YH, Lazo GR (2001). Molecular mapping and characterization of traits controlling fiber quality in cotton. Euphytica.

[CR19] Lacape JM, Nguyen TB, Courtois B, Belot JL, Giband M, Gourlot JP, Gawryziak G, Roques S, Hau B (2005). QTL analysis of cotton fiber quality using multiple *Gossypium hirsutum* × *Gossypium barbadense* backcross generations. Crop Sci.

[CR20] Lacape JM, Jacobs J, Arioli T, Derijcker R, Forestier-Chiron N, Llewellyn D, Jean J, Thomas E, Viot C (2009). A new interspecific, *Gossypium hirsutum* × *G. barbadense*, RIL population: towards a unified consensus linkage map of tetraploid cotton. Theor Appl Genet.

[CR21] Lacape JM, Llewellyn D, Jacobs J, Arioli T, Becker D, Calhoun S, Al-Ghazi Y, Liu S, Palai O, Georges S, Giband M, de Assuncao H, Barroso PA, Claverie M, Gawryziak G, Jean J, Vialle M, Viot C (2010). Meta-analysis of cotton fiber quality QTLs across diverse environments in a *Gossypium hirsutum* × *G. barbadense* RIL population. BMC Plant Biol.

[CR22] Li H, Ye G, Wang J (2007). A modified algorithm for the improvement of composite interval mapping. Genetics.

[CR100] Li FG, Fan GY, Wang KB, Sun FM (2014). Genome sequence of the cultivated cotton Gossypium arboreum. Nat Genet.

[CR23] Li F, Fan G, Lu C, Xiao G (2015). Genome sequence of cultivated Upland cotton (*Gossypium hirsutum* TM-1) provides insights into genome evolution. Nat Biotechnol.

[CR24] Li B, Shi Y, Gong J, Li J, Liu A, Shang H, Gong W, Chen T, Ge Q, Jia C, Lei Y, Hu Y, Yuan Y (2016). Genetic effects and heterosis of yield and yield component traits based on *Gossypium barbadense* chromosome segment substitution lines in two *Gossypium hirsutum* backgrounds. PLoS ONE.

[CR25] Li PT, Wang M, Lu QW (2017). Comparative transcriptome analysis of cotton fiber development of Upland cotton (*Gossypium hirsutum*) and chromosome segment substitution lines from *G. hirsutum* × *G. barbadense*. BMC Genom.

[CR26] Li PT, Rashid MHO, Chen TT (2019). Transcriptomic and biochemical analysis of upland cotton (*Gossypium hirsutum*) and a chromosome segment substitution line from *G. hirsutum* × *G. barbadense* in response to *Verticillium dahliae* infection. BMC Plant Biol.

[CR27] Li SQ, Liu AY, Kong LL, Gong JW, Li JW, Gong WK, Lu QW, Li PT, Ge Q, Shang HH, Xiao XH, Liu RX, Zhang Q, Shi YZ, Yuan YL (2019). QTL mapping and genetic effect of chromosome segment substitution lines with excellent fiber quality from *Gossypium hirsutum* × *Gossypium barbadense*. Mol Genet Genom.

[CR28] Lin Z, He D, Zhang X, Nie Y, Guo X, Feng C, Stewart JM (2005). Linkage map construction and mapping QTL for cotton fibre quality using SRAP, SSR and RAPD. Plant Breed.

[CR29] Lu Q, Shi Y, Xiao X, Li P, Gong J, Gong W, Liu A, Shang H, Li J, Ge Q, Song W, Li S, Zhang Z, Rashid MHO, Peng R, Yuan Y, Huang J (2017). Transcriptome analysis suggests that chromosome introgression fragments from Sea Island cotton (*Gossypium barbadense*) increase fiber strength in Upland cotton (*Gossypium hirsutum*). G3 (Bethesda).

[CR30] Ma L, Shi Y, Lan M, Yang Z, Zhang J, Zhang B, Li J, Wang T, Gong J, Liu A, Shang H, Gong W, Yuan Y (2013). Evaluation of chromosome segment substitution lines related to fiber yield and quality traits from *Gossypium hirsutum *× *Gossypium Barbadense*. Cotton Sci.

[CR31] Ma LL, Zhao YP, Wang YM, Shang LG, Hua JP (2017). QTLs analysis and validation for fiber quality traits using maternal backcross population in Upland cotton. Front Plant Sci.

[CR32] Mei M, Syed NH, Gao W, Thaxton PM, Smith CW, Stelly DM, Chen ZJ (2004). Genetic mapping and QTL analysis of fiber-related traits in cotton (*Gossypium*). Theor Appl Genet.

[CR33] Monforte AJ, Tanksley SD (2000). Development of a set of near isogenic and backcross recombinant inbred lines containing most of the Lycopersicon *hirsutum* genome in a *L. esculentum* genetic background: a tool for gene mapping and gene discovery. Genome.

[CR34] Nie X, Tu J, Wang B, Zhou X, Lin Z (2015). A BIL population derived from *G. hirsutum* and *G. barbadense* provides a resource for cotton genetics and breeding. PLoS ONE.

[CR35] Ning ZY, Chen H, Mei HX, Zhang TZ (2014). Molecular tagging of QTLs for fiber quality and yield in the upland cotton cultivar Acala-Prema. Euphytica.

[CR36] Okada S, Onogi A, Iijima K, Hori K, Iwata H, Yokoyama W, Suehiro M, Yamasaki M (2018). Identification of QTLs for rice grain size using a novel set of chromosomal segment substitution lines derived from Yamadanishiki in the genetic background of Koshihikari. Breed Sci.

[CR37] Paterson AH, Brubaker CL, Wendel JF (1993). A rapid method for extraction of cotton (*Gossypium* spp.) genomic DNA suitable for RFLP or PCR analysis. Plant Mol Biol Rep.

[CR38] Paterson AH, Saranga Y, Menz M, Jiang CX, Wright RJ (2003). QTL analysis of genotype× environment interactions affecting cotton fiber quality. Theor Appl Genet.

[CR39] Qi H, Huang J, Zheng Q, Huang Y, Shao R, Zhu L, Zhang Z, Qiu F, Zhou G, Zheng Y, Yue B (2013). Identification of combining ability loci for five yield-related traits in maize using a set of testcrosses with introgression lines. Theor Appl Genet.

[CR40] Qin H, Guo W, Zhang YM, Zhang T (2008). QTL mapping of yield and fiber traits based on a four-way cross population in *Gossypium hirsutum* L. Theor Appl Genet.

[CR41] Qiu X, Chen K, Lv W, Ou X, Zhu Y, Xing D, Yang L, Fan F, Yang J, Xu J, Zheng T, Li Z (2017). Examining two sets of introgression lines reveals background-independent and stably expressed QTL that improve grain appearance quality in rice (*Oryza sativa* L.). Theor Appl Genet.

[CR42] Saha S, Wu J, Jenkins JN, McCarty JC, Stelly DM (2013). Interspecific chromosomal effects on agronomic traits in *Gossypium hirsutum* by AD analysis using intermated *G. barbadense* chromosome substitution lines. Theor Appl Genet.

[CR43] Said JI, Lin Z, Zhang X, Song M, Zhang J (2013). A comprehensive meta QTL analysis for fiber quality, yield, yield related and morphological traits, drought tolerance, and disease resistance in tetraploid cotton. BMC Genom.

[CR44] Said JI, Knapka JA, Song M, Zhang J (2015). Cotton QTLdb: a cotton QTL database for QTL analysis, visualization, and comparison between *Gossypium hirsutum* and *G. hirsutum* × *G. barbadense* populations. Mol Genet Genom.

[CR45] Said JI, Song M, Wang H, Lin Z, Zhang X, Fang DD, Zhang J (2015). A comparative meta-analysis of QTL between intraspecific *Gossypium hirsutum* and interspecific *G. hirsutum* × *G. barbadense* populations. Mol Genet Genom.

[CR46] Shao QS, Zhang FJ, Tang SY, Liu Y, Fang XM, Liu DX, Liu DJ, Zhang J, Teng ZH, Paterson AH, Zhang ZS (2014). Identifying QTL for fiber quality traits with three upland cotton (*Gossypium hirsutum* L.) populations. Euphytica.

[CR47] Shen XL, Guo WZ, Zhu XF, Yuan YL, Yu JZ, Kohel RJ, Zhang TZ (2005). Molecular mapping of QTLs for fiber qualities in three diverse lines in Upland cotton using SSR markers. Mol Breed.

[CR48] Shi Y, Li W, Li A (2015). Constructing a high-density linkage map for *Gossypium hirsutum *× *Gossypium barbadense* and identifying QTLs for lint percentage. J Integr Plant Biol.

[CR49] Shi Y, Zhang B, Liu A, Li W, Li J, Lu Q, Zhang Z, Li S, Gong W, Shang H, Gong J, Chen T, Ge Q, Wang T, Zhu H, Liu Z, Yuan Y (2016). Quantitative trait loci analysis of *Verticillium wilt* resistance in interspecific backcross populations of *Gossypium hirsutum *× *Gossypium barbadense*. BMC Genom.

[CR50] Shi Y, Liu A, Li J, Zhang J, Zhang B, Ge Q, Jamshed M, Lu Q, Li S, Xiang X, Gong J, Gong W, Shang H, Deng X, Pan J, Yuan Y (2019). Dissecting the genetic basis of fiber quality and yield traits in interspecific backcross populations of *Gossypium hirsutum* × *Gossypium barbadense*. Mol Genet Genom.

[CR51] Song W, Wang M, Su W, Lu Q, Xiao X, Cai J, Zhang Z, Li S, Li P, Gong J, Gong W, Shang H, Liu A, Li J, Chen T, Ge Q, Shi Y, Yuan Y (2017). Genetic and phenotypic effects of chromosome segments introgressed from *Gossypium barbadense* into *Gossypium hirsutum*. PLoS ONE.

[CR52] Stelly DM, Saha S, Raska DA, Jenkins JN, McCarty JC, Gutierrez OA (2005). Registration of 17 upland (*Gossypium hirsutum*) cotton germplasm lines disomic for different *G. barbadense* chromosome or arm substitutions. Crop Sci.

[CR53] Su CF, Wang W, Qiu XM, Yang L, Li S, Wang MX, Pan QB (2013). Fine-mapping a fibre strength QTL *qFS*-*D11*-*1* on cotton chromosome 21 using introgressed lines. Plant Breed.

[CR54] Sun FD, Zhang JH, Wang SF, Gong WK, Shi YZ, Liu AY, Li JW, Gong JW, Shang HH, Yuan YL (2012). QTL mapping for fiber quality traits across multiple generations and environments in upland cotton. Mol Breed.

[CR55] Sun Z, Wang X, Liu Z, Gu Q, Zhang Y, Li Z, Ke H, Yang J, Wu J, Wu L, Zhang G, Zhang C, Ma Z (2017). Genome-wide association study discovered genetic variation and candidate genes of fibre quality traits in *Gossypium hirsutum* L. Plant Biotechnol J.

[CR56] Tan ZY, Zhang ZQ, Sun XJ, Li QQ, Sun Y, Yang P, Wang WW, Liu XY, Chen CL, Liu DX, Teng ZH, Guo K, Zhang J, Liu DJ, Zhang ZS (2018). Genetic map construction and fiber quality QTL mapping using the CottonSNP80K array in Upland cotton. Front Plant Sci.

[CR57] Tang SY, Teng ZH, Zhai TF, Fang XM, Liu F, Liu DJ, Zhang J, Liu DX, Wang SF, Zhang K, Shao QS, Tan ZY, Paterson AH, Zhang ZS (2015). Construction of genetic map and QTL analysis of fiber quality traits for Upland cotton (*Gossypium hirsutum* L.). Euphytica.

[CR58] van Berloo R (2008). GGT 2.0: versatile software for visualization and analysis of genetic data. J Hered.

[CR59] Voorrips RE (2002). MapChart: software for the graphical presentation of linkage maps and QTLs. J Hered.

[CR60] Wan XY, Wan JM, Weng JF, Jiang L, Bi JC, Wang CM, Zhai HQ (2005). Stability of QTLs for rice grain dimension and endosperm chalkiness characteristics across eight environments. Theor Appl Genet.

[CR61] Wang P, Ding YZ, Lu QX, Guo WZ, Zhang TZ (2008). Development of *Gossypium barbadense* chromosome segment substitution lines in the genetic standard line TM-1 of *Gossypium hirsutum*. Chin Sci Bull.

[CR62] Wang FR, Gong YC, Zhang CY, Liu GD, Wang LM, Xu ZZ, Zhang J (2011). Genetic effects of introgression genomic components from Sea Island cotton (*Gossypium barbadense* L.) on fiber related traits in upland cotton (*G. hirsutum* L.). Euphytica.

[CR63] Wang P, Zhu Y, Song X, Cao Z, Ding Y, Liu B, Zhu X, Wang S, Guo W, Zhang T (2012). Inheritance of long staple fiber quality traits of *Gossypium barbadense* in *G. hirsutum* background using CSILs. Theor Appl Genet.

[CR64] Wang XQ, Yu Y, Li W, Guo HL, Lin ZX, Zhang XL (2013). Association analysis of yield and fiber quality traits in *Gossypium barbadense* with SSRs and SRAPs. Genet Mol Res.

[CR65] Wang Y, Zang JP, Sun Y, Ali J, Xu JL, Li ZK (2013). Background-independent quantitative trait loci for drought tolerance identified using advanced backcross introgression lines in rice. Crop Sci.

[CR66] Wang Y, Ning Z, Hu Y, Chen J, Zhao R, Chen H, Ai N, Guo W, Zhang T (2015). Molecular mapping of restriction-site associated DNA markers in allotetraploid Upland cotton. PLoS One.

[CR67] Wang FR, Zhang CY, Liu GD, Chen Y, Zhang JX, Qiao QH, Yuan ZC, Fan SJ, Zhang J (2016). Phenotypic variation analysis and QTL mapping for cotton (*Gossypium hirsutum* L.) fiber quality grown in different cotton-producing regions. Euphytica.

[CR68] Wang BH, Draye X, Zhuang ZM, Zhang ZS, Liu M, Lubbers EL, Jones D, May OL, Paterson AH, Chee PW (2017). QTL analysis of cotton fiber length in advanced backcross populations derived from a cross between *Gossypium hirsutum* and *G*-*mustelinum*. Theor Appl Genet.

[CR69] Wang BH, Zhuang ZM, Zhang ZS, Draye X, Shuang LS, Shehzad T, Lubbers EL, Jones D, May OL, Paterson AH, Chee PW (2017). Advanced backcross QTL analysis of fiber strength and fineness in a cross between *Gossypium hirsutum* and *G. mustelinum*. Front Plant Sci.

[CR70] Wang M, Tu L, Yuan D (2019). Reference genome sequences of two cultivated allotetraploid cottons, *Gossypium hirsutum* and *Gossypium barbadense*. Nat Genet.

[CR71] Wu J, Jenkins JN, McCarty JC, Saha S, Stelly DM (2006). An additive-dominance model to determine chromosomal effects in chromosome substitution lines and other gemplasms. Theor Appl Genet.

[CR72] Xu P, Gao J, Cao Z, Chee PW, Guo Q, Xu Z, Paterson AH, Zhang X, Shen X (2017). Fine mapping and candidate gene analysis of *qFL*-*chr1*, a fiber length QTL in cotton. Theor Appl Genet.

[CR73] Yang Z, Li J, Li A, Zhang B, Liu G, Li J, Shi Y, Liu A, Jiang J, Tao Wang, Yuan Y (2009). Developing chromosome segment substitution lines (CSSLs) in cotton (*Gossypium*) using advanced backcross and MAS. Mol Plant Breed.

[CR74] Yang XL, Zhou XD, Wang XF, Li ZK, Zhang Y, Liu HW, Wu LQ, Zhang GY, Yan GJ, Ma ZY (2015). Mapping QTL for cotton fiber quality traits using simple sequence repeat markers, conserved intron-scanning primers, and transcript-derived fragments. Euphytica.

[CR75] Yu JW, Zhang K, Li SY, Yu SX, Zhai HH, Wu M, Li XL, Fan SL, Song MZ, Yang DG, Li YH, Zhang JF (2013). Mapping quantitative trait loci for lint yield and fiber quality across environments in a *Gossypium hirsutum* × *Gossypium barbadense* backcross inbred line population. Theor Appl Genet.

[CR76] Zhai H, Gong W, Tan Y, Liu A, Song W, Li J, Deng Z, Kong L, Gong J, Shang H, Chen T, Ge Q, Shi Y, Yuan Y (2016). Identification of chromosome segment substitution lines of *Gossypium barbadense* introgressed in *G. hirsutum* and quantitative trait locus mapping for fiber quality and yield traits. PLoS One.

[CR77] Zhang K, Zhang J, Ma J, Tang SY, Liu DJ, Teng ZH, Liu DX, Zhang ZS (2012). Genetic mapping and quantitative trait locus analysis of fiber quality traits using a three-parent composite population in upland cotton (*Gossypium hirsutum* L.). Mol Breed.

[CR78] Zhang SW, Zhu XF, Feng LC, Gao X, Yang B, Zhang TZ, Zhou BL (2016). Mapping of fiber quality QTLs reveals useful variation and footprints of cotton domestication using introgression lines. Sci Rep.

[CR79] Zhao X, Daygon VD, McNally KL, Hamilton RS, Xie F, Reinke RF, Fitzgerald MA (2016). Identification of stable QTLs causing chalk in rice grains in nine environments. Theor Appl Genet.

[CR80] Zheng TQ, Wang Y, Ali AJ, Zhu LH, Sun Y, Zhai HQ, Mei HW, Xu ZJ, Xu JL, Li ZK (2011). Genetic effects of background-independent loci for grain weight and shape identified using advanced reciprocal introgression lines from Lemont × Teqing in rice. Crop Sci.

