# Supplementary figures and images for: Examining two sets of introgression lines across multiple environments reveals background-independent and stably expressed quantitative trait loci of fiber quality in cotton
Source: Theor Appl Genet. 2020 Mar 17;133(7):2075–93. doi: 10.1007/s00122-020-03578-0 (PMC7311500; doi:10.1007/s00122-020-03578-0)

36Pop

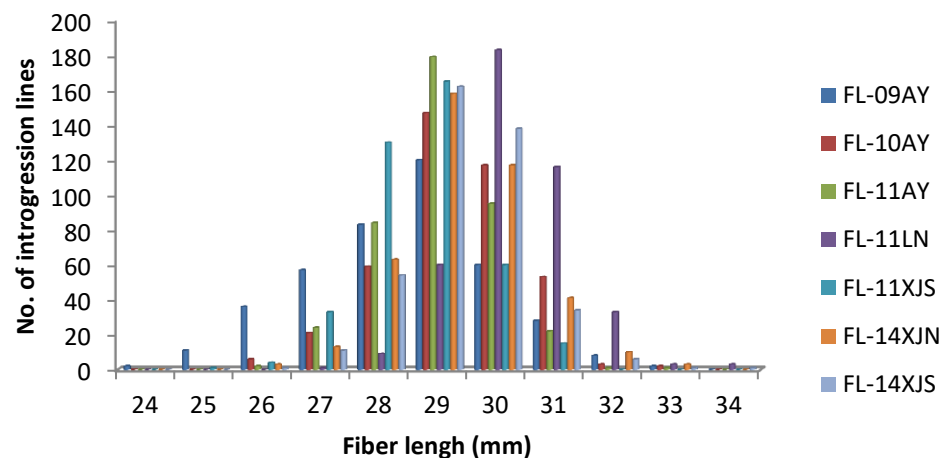

45Pop

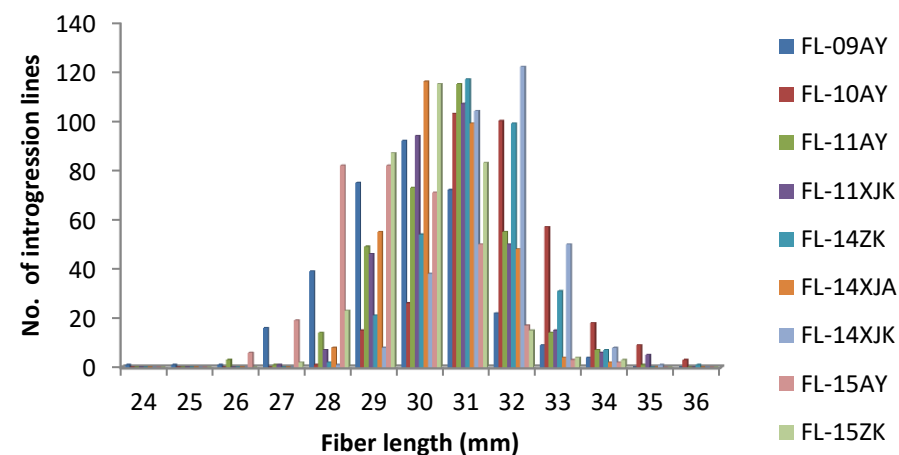

36Pop

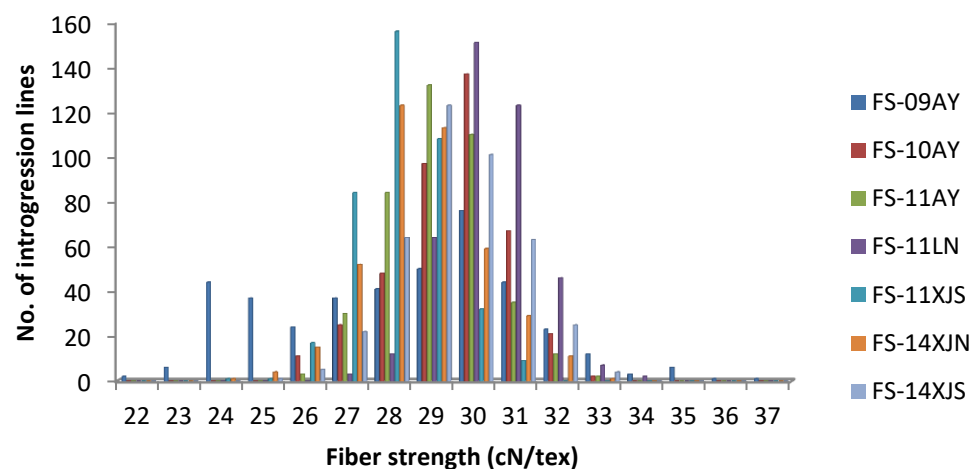

45Pop

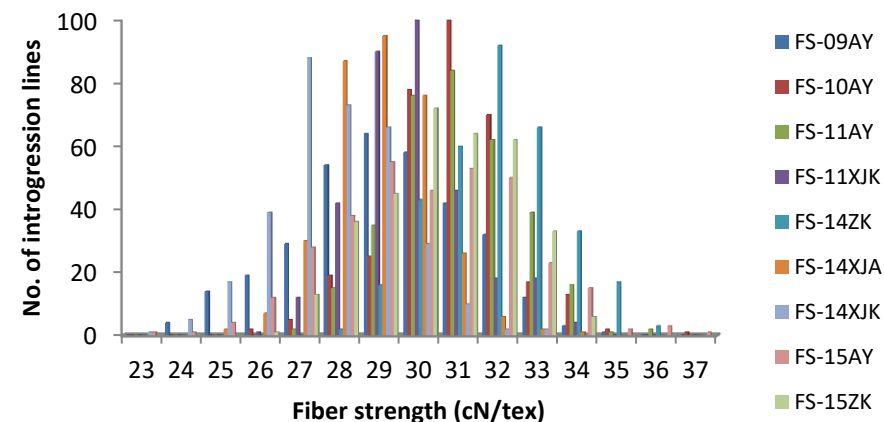

36Pop

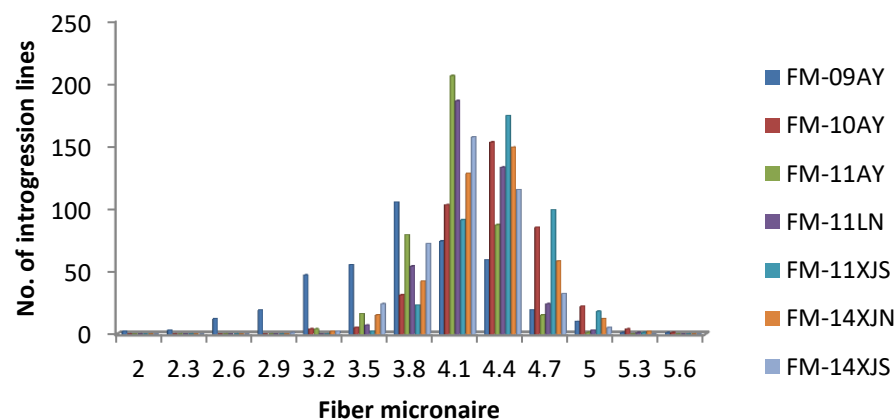

45Pop

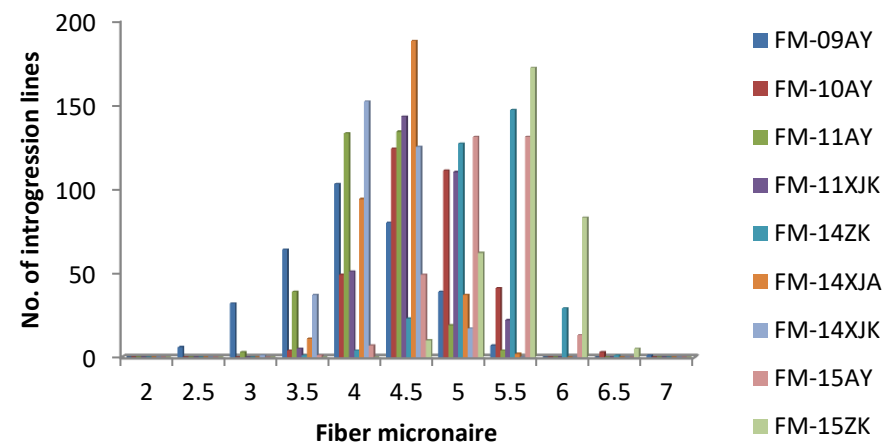

Supplement: Supplementary file 1 — Fig. S1 Frequency distribution of fiber quality traits in the two CSSL populations. (PDF 393 kb) [file 122_2020_3578_MOESM1_ESM.pdf]

C1

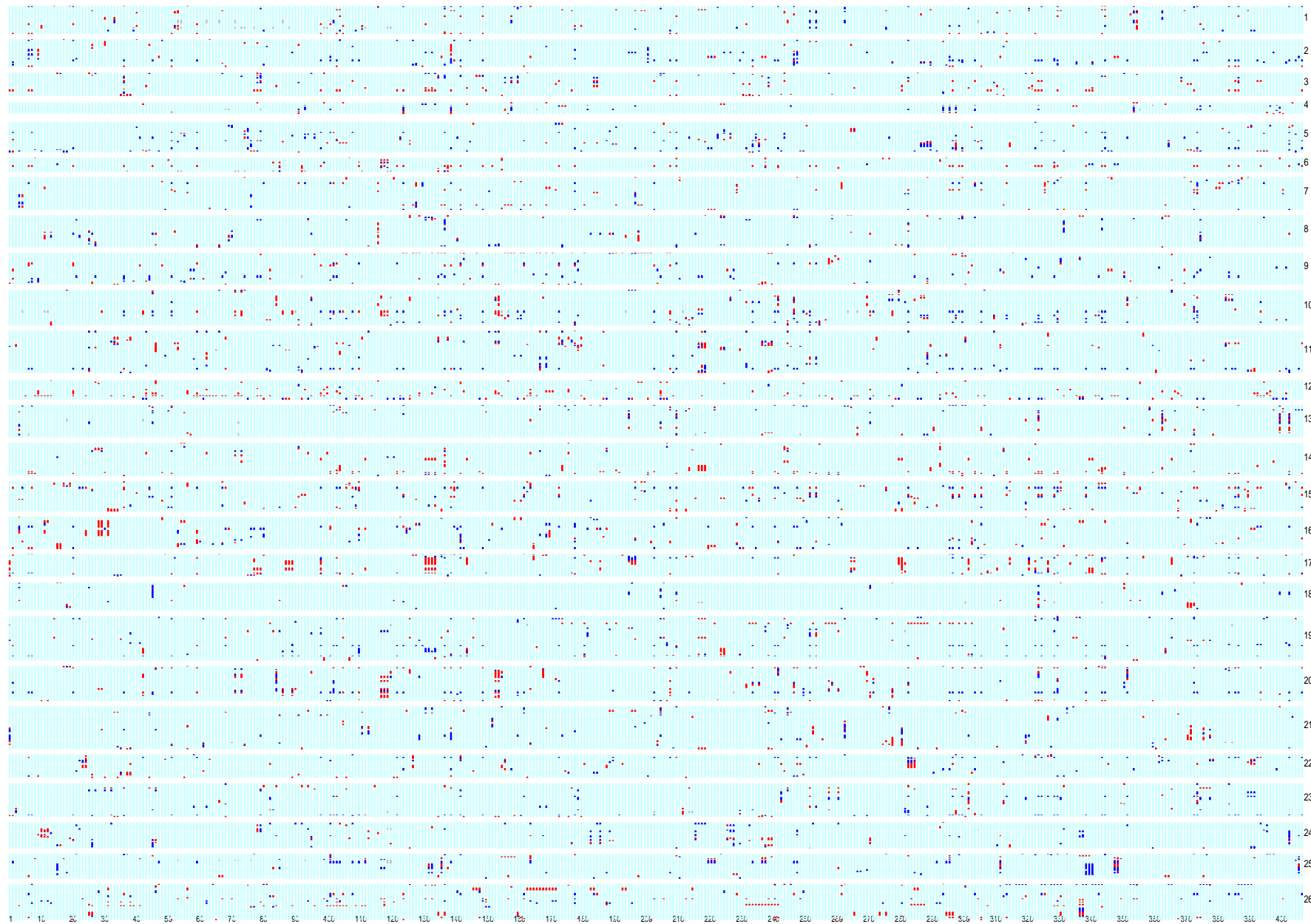

C26

1

→ 408 (lines)

Legend  A  B  H  U

Supplement: Supplementary file 2 — Fig. S2 Introgressed Hai1 chromosome segments in the 36Pop population of CSSLs with the CCRI36 genetic background. (PDF 905 kb) [file 122_2020_3578_MOESM2_ESM.pdf]

C1

C26

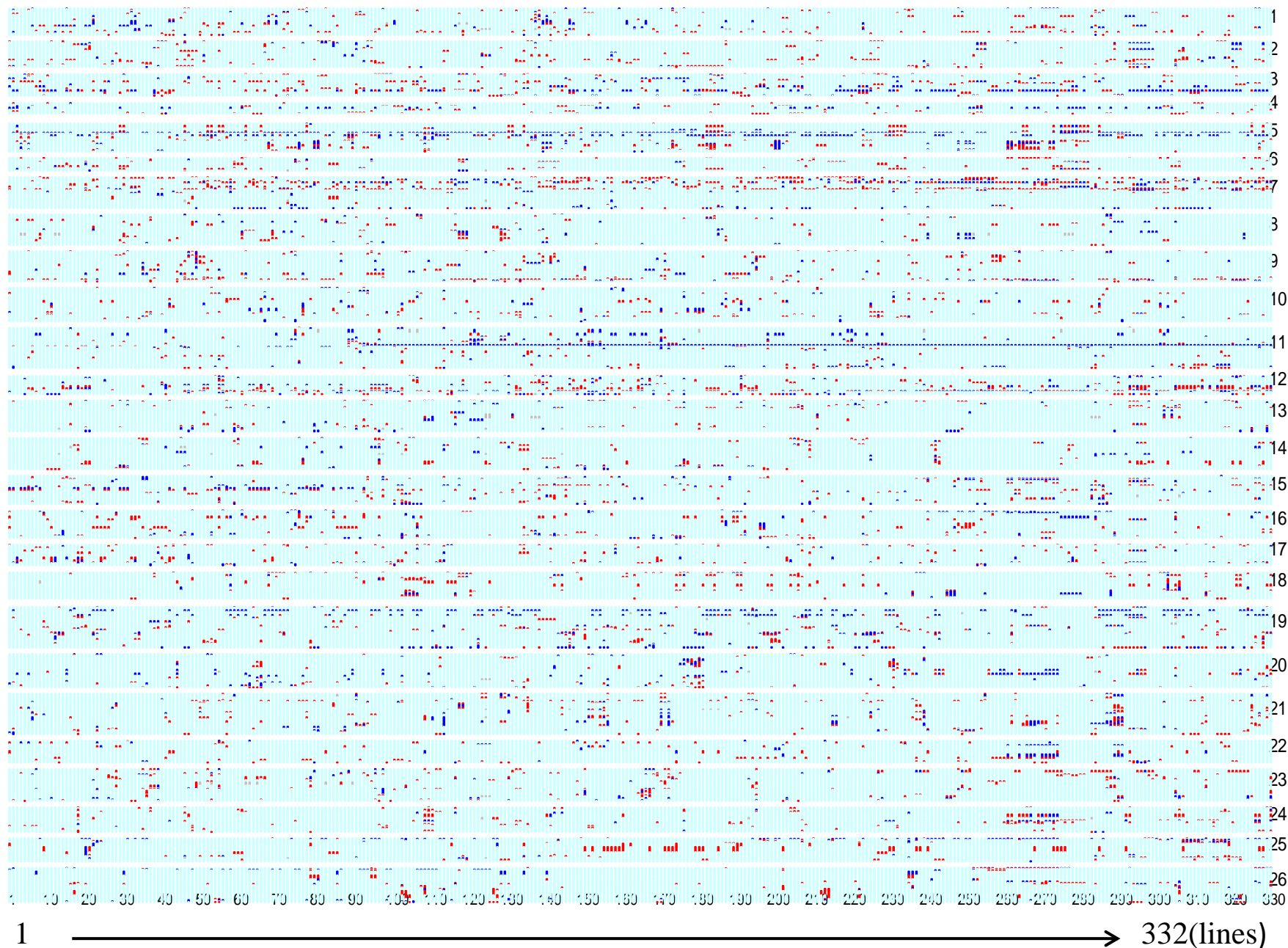

Legend  A  B  H  U

Supplement: Supplementary file 3 — Fig. S3 Introgressed Hai1 chromosome segments in the 45Pop population of CSSLs with the CCRI45 genetic background. (PDF 758 kb) [file 122_2020_3578_MOESM3_ESM.pdf]
