# Supplementary material for: Examining two sets of introgression lines across multiple environments reveals background-independent and stably expressed quantitative trait loci of fiber quality in cotton
Source: Theor Appl Genet. 2020 Mar 17;133(7):2075–93. doi: 10.1007/s00122-020-03578-0 (PMC7311500; doi:10.1007/s00122-020-03578-0)
Supplement: Supplementary file 4 — Table S1 Details of selected markers from the genetic linkage map of the CCRI36 × Hai1 BC1F1 population. (PDF 754 kb) [file 122_2020_3578_MOESM4_ESM.pdf]

Table S1 Details of selected markers on the genetic linkage map of CCRI36 ×Hai1 BC_1F_1 population

marker loci	Chromosome	Position	Selected marker
HAU0887	1	0.00	Yes
DPL0833	1	2.71	
HAU1619	1	4.84	Yes
CGR5524	1	6.97	
CGR5417	1	10.76	Yes
HAU0282	1	13.60	
Gh075	1	14.11	
CGR5995	1	16.82	Yes
NAU5100	1	24.25	
HAU0606	1	26.38	
NAU4074	1	27.95	Yes
DC20076	1	29.53	
HAU1229	1	30.03	
TMB0119a	1	31.07	Yes
TMB1224a	1	31.68	
TMB2544	1	34.29	
BNL3888	1	34.29	
MUSS310	1	37.29	Yes
CGR5853	1	40.30	
DPL0094	1	40.30	
DPL0692	1	42.73	
NAU3744b	1	45.15	Yes
PGML00665	1	47.28	
DPL0023b	1	47.79	
DPL0109a	1	48.04	
DPL0386	1	51.06	
CICR0256	1	53.48	
DPL0513	1	53.48	
JESPR056b	1	55.33	Yes
Gh649b	1	55.58	
PGML00698	1	58.89	
MUCS084	1	70.29	Yes
Gh216	1	77.38	
BNL2921	1	77.98	Yes
CGR6870	1	90.23	Yes
TMB1421	1	107.29	
CGR6378	1	107.57	Yes
PGML01081	1	110.57	
CGR6356	1	118.79	
TMB1152b	1	122.66	Yes
CICR0894	1	125.98	
NAU5411	1	126.49	
NAU5163	1	126.74	
CGR5056b	1	131.66	Yes
DPL0752	1	136.57	
PGML0280	1	145.63	Yes
CGR6803a	1	150.21	
CGR6803c	1	152.93	
CICR0105	1	153.69	
CICR0179	1	153.69	
NAU2095	1	154.73	Yes
CGR5326b	1	155.76	
DPL0346b	1	162.13	Yes
SWU0280a	1	170.93	
BNL2440	1	175.54	Yes
CGR5020	1	178.29	
NAU3901	1	182.22	Yes
NAU3535	2	0.00	Yes

PGML02861a	2	8.99	Yes
DPL0450	2	12.08	
PGML04760	2	14.80	
BNL1434	2	15.12	Yes
CGR5250	2	16.79	
PGML01061	2	18.10	
CER0058	2	23.53	Yes
TMB2386	2	29.79	
BNL1897	2	39.33	Yes
DC40265	2	43.60	
CGR5876a	2	44.92	
CICR0930	2	47.64	
TMB1578	2	50.01	Yes
DPL0315	2	51.32	
HAU1980b	2	54.78	Yes
Gh669	2	54.78	
SHIN-1481a	2	58.62	
CGR6716a	2	58.95	
CGR6729a	2	59.27	
DPL0004	2	61.63	
DPL0539	2	64.35	
MUSS294	2	65.33	
NAU5134	2	67.34	
CICR0239	2	69.35	
BNL3590	2	72.44	Yes
JESPR101a	2	72.44	
BNL3971	2	74.10	
NAU2501	2	74.10	
PGML03028	2	78.33	
NAU3875	2	79.99	Yes
DPL0041	2	84.88	Yes
TMB0471	2	92.83	
HAU2132	2	100.06	Yes
Gh198a	2	103.15	
TMB0514	2	103.15	
PGML03818	2	105.15	
NAU1072	2	110.58	
CGR5201b	2	118.16	Yes
CGR5385	2	127.16	
SHIN-1584	2	129.18	
CICR0800	2	129.83	Yes
CICR0489	2	130.81	
HAU1155	2	132.47	
HAU0880	2	137.50	Yes
HAU2690	2	145.56	
DPL0245a	2	149.78	
HAU1475	2	154.42	Yes
TMB1580	2	159.08	Yes
HAU2001	2	164.53	Yes
HAU0800b	2	165.85	
NAU3775	2	166.83	
HAU3182	2	169.92	Yes
NAU1246	2	171.24	
HAU0877	2	172.56	
NAU2277	2	178.82	Yes
CICR0764	3	0.00	
HAU0883b	3	2.70	
NAU0833	3	3.42	
NAU5289	3	3.56	Yes
DPL0502b	3	4.42	
HAU0263	3	4.85	

CIR084	3	5.39	
HAU2024	3	5.55	
NAU3464	3	8.54	Yes
DC40396	3	11.35	
DPL0921	3	13.60	
HAU0857	3	17.01	Yes
HAU0875	3	17.87	
HAU1322	3	18.59	
HAU0203	3	19.76	
CGR5996	3	19.76	
Gh210	3	19.76	
TMB1989	3	20.48	Yes
PGML04299	3	22.47	
CGR6874	3	23.92	
DPL0195a	3	24.53	
HAU2511	3	25.10	
CICR0417	3	25.97	
DPL0268	3	26.54	Yes
SHIN-0659a	3	27.56	
HAU3185	3	29.90	
MGHES69	3	32.25	
NAU5469	3	33.27	
CICR0376	3	34.93	
DC40402	3	35.79	
DC20082	3	36.81	
DC40064	3	37.24	
MUSS424	3	37.52	Yes
PGML01520	3	39.23	
DPL0038	3	41.51	
CGR5466	3	42.38	
BNL0542	3	44.64	
PGML04787	3	47.65	Yes
DPL0680	3	53.63	
CICR0437	3	56.90	
NAU1190	3	57.62	Yes
CICR0222	3	58.80	
Gh129	3	60.13	
DPL0170	3	63.02	
CGR6728b	3	66.88	
DPL0427b	3	67.80	
DC20155	3	69.45	
TMB0564	3	71.56	Yes
DPL0506	3	72.80	
NAU0856	3	74.79	Yes
SWU1214	3	76.79	
DPL0842	3	78.78	
NAU2639	3	80.77	
HAU0604	3	80.91	
DPL0555	3	81.48	
DPL0901	3	85.36	
CICR0121	3	90.73	
MUSB0087	3	93.70	Yes
PGML03765	3	95.19	
NAU1294	3	96.06	
HAU1286	3	96.48	
BNL3441	3	96.97	
CER0028	3	97.48	
DPL0232	3	99.83	
PGML03195	3	103.51	
TMB1898	3	105.01	
HAU1022	3	105.29	Yes

BNL2443	3	106.78	
DPL0224	3	107.21	
CICR0034	3	108.54	
NAU3479	3	111.06	
HAU0912	3	111.78	
NAU2297	3	112.35	Yes
HAU1396	3	113.84	
PGML02906	3	113.84	
DPL0321	3	114.12	
HAU0195b	3	114.40	Yes
CGR6873	3	114.83	
CGR6528	3	115.11	
DPL0291	3	118.25	
HAU3403	3	128.02	Yes
PGML00950b	3	134.54	
NAU3016a	3	141.35	Yes
PGML03307	3	145.85	
HAU0292	3	146.63	Yes
HAU0759	3	148.57	Yes
CGR5620	3	150.74	
CGR5729	3	151.03	
NAU3083	3	153.72	Yes
MUCS547	3	166.15	
DPL0573	4	0.00	
CICR0640	4	5.73	
MUSB0264	4	6.40	Yes
NAU2701	4	7.22	
HAU3050b	4	14.75	
DPL0137a	4	23.61	Yes
NAU1158	4	26.12	Yes
CICR0020	4	26.83	
HAU1444	4	27.23	
NAU0786	4	28.82	Yes
CGR5812	4	31.37	
TMB0809	4	32.18	
NAU3825	4	32.18	
CGR5252	4	32.18	
DC40436	4	32.45	
NAU5180	4	33.41	
DC40149	4	36.16	
DPL0685	4	44.93	Yes
PGML02232	4	55.27	Yes
DPL0107a	4	61.61	Yes
HAU3228	4	63.09	
DPL0704	4	64.35	
HAU1300	4	65.45	
DPL0085	4	66.71	Yes
NAU2363	4	69.09	
HAU1215	4	70.05	
Gh117	4	73.91	Yes
NAU3093	4	77.37	Yes
HAU2016	4	77.50	
DPL0451	4	78.46	
HAU0101	4	80.03	Yes
CICR0345	4	81.28	
BNL2572	4	81.68	
CICR0267	4	83.09	
MUCS570	4	85.82	
NAU5236	4	90.32	
CER0139a	4	90.72	
DPL0014	5	0.00	

PGML04317	5	9.85	
PGML04350a	5	15.15	
COT130	5	18.71	Yes
DPL0247a	5	25.61	Yes
CIR224b	5	30.46	Yes
PGML03048	5	31.31	
CIR102	5	32.29	
DC20067	5	32.43	
NAU4034	5	32.43	
BNL1042	5	32.56	
DPL0063	5	35.37	
HAU0746	5	38.17	
TMB1296	5	38.17	
CGR6708a	5	38.58	
DPL0724	5	38.99	
TMB1120	5	39.12	
CGR5590	5	39.39	
PGML02063	5	39.80	
CGR5025	5	40.21	
DPL0022	5	41.66	
HAU1712	5	43.10	
MGHES06	5	43.94	
DPL0838	5	44.64	
NAU2494	5	44.77	
DPL0138	5	45.04	
DPL0241	5	47.41	Yes
NAU1372	5	49.00	
CGR6247	5	50.21	
HAU1030	5	50.34	
DC30048	5	50.48	
HAU0871	5	50.75	
NAU1109	5	50.88	
NAU0922	5	50.88	
CICR0065b	5	51.58	
DPL0368	5	53.18	Yes
DPL0554	5	58.99	
DC40122	5	60.77	
CIR139b	5	61.37	
MUCS530	5	64.32	Yes
HAU1034	5	67.28	
NAU5160	5	67.71	
HAU0869	5	69.48	Yes
NAU3325	5	70.46	
CGR5582	5	72.39	
DPL0210b	5	75.61	
NAU3416	5	82.36	Yes
MUSS317	5	89.92	Yes
MUSS118	5	94.09	Yes
MUCS108	5	94.36	
NAU0826	5	94.36	
NAU1015	5	94.49	
HAU2785	5	96.76	
DPL0040b	5	97.03	
NAU1339	5	97.44	
CICR0111	5	98.28	
BNL2448	5	100.04	Yes
DPL0155a	5	100.04	
CGR5135	5	100.88	
BNL3992	5	102.48	
HAU1500	5	104.52	
TMB0131b	5	105.87	

DPL0246	5	107.16	
HAU1384	5	107.86	Yes
HAU1385a	5	109.15	
Gh083	5	112.70	
HAU3395	5	119.29	
DPL0384	5	123.29	Yes
MGHES59	5	126.55	
BNL0226	5	130.70	
DPL0174b	5	135.59	
HAU1976	5	139.21	
HAU0506	5	139.35	Yes
NAU2957	5	141.28	Yes
HAU1957	5	141.55	
SWU0916	5	145.30	
DC20163	5	147.92	
Gh594	5	149.21	Yes
Gh211	5	149.76	
CGR6733	5	151.05	
HAU1404	5	152.03	
DPL0908a	5	153.80	
HAU0215	5	156.79	
NAU3824a	5	158.09	Yes
BNL3995	5	159.84	
NAU1127	5	159.98	
DPL0637	5	160.25	
Gh166b	5	160.66	
DPL0327	5	161.54	
CIR301	5	164.47	
DPL0276	5	165.02	
NAU2001	5	165.30	Yes
CICR0496	5	165.71	
NAU1200	5	166.40	
COT010	5	167.39	
HAU3350b	5	167.94	
CGR5925a	5	168.63	
DPL0622	5	169.18	
NAU2296b	5	172.74	Yes
NAU2296a	5	174.50	Yes
NAU3138	5	181.66	
HAU1496	5	196.86	
NAU3036	5	197.41	Yes
HAU1050	5	197.41	
PGML00939	5	199.34	
CIR253	5	201.60	
TMB0770a	5	202.44	Yes
TMB0770b	5	202.44	
NAU5400	5	203.43	
SWU1250	5	204.57	
NAU3402	5	205.12	Yes
CGR5553	5	209.95	Yes
PGML00120	5	209.95	
NAU2948	5	218.04	Yes
NAU5038	6	0.00	Yes
DPL0328	6	1.19	Yes
NAU3173	6	1.76	
CGR5271	6	2.37	
CGR5128b	6	3.92	
NAU2967	6	4.03	
TMB0853	6	4.37	
TMB0154	6	4.37	
PGML04263	6	4.94	

TMB1538	6	5.45	
Gh032	6	5.62	
NAU0796	6	5.62	
DPL0222	6	11.53	
CGR6932b	6	14.62	
NAU4969	6	14.73	
CGR5525b	6	15.31	
HAU0964	6	16.00	
DC40067	6	17.07	
COT002	6	18.26	Yes
DPL0918a	6	18.71	
DPL0166b	6	20.87	
DPL0847	6	22.33	Yes
DPL0427a	6	22.90	
CGR6749	6	23.47	
HAU3308	6	24.04	
PGML04892	6	24.86	
DPL0153	6	27.95	
Gh082	6	29.82	Yes
BNL1440b	6	31.10	Yes
HAU2447	6	31.85	
HAU3350a	6	36.62	
DPL0142	6	38.35	
DPL0697a	6	40.80	
BNL4108	6	42.26	
TMB2959	6	42.26	
CER0093	6	42.26	
TMB1277	6	42.26	
HAU1488	6	42.26	
NAU2838	6	42.26	
HAU0091	6	42.49	
NAU0905a	6	42.82	Yes
HAU1293	6	43.05	
DPL0392	6	43.27	
CGR5562	6	43.27	
CGR6814	6	43.27	
CER0086a	6	44.46	
PGML00996	6	45.92	
C2-0009B	6	46.61	
CGR6019	6	49.27	
PGML01861	6	54.15	Yes
DC40417	6	55.00	
CGR5355	6	55.22	
CICR0461b	6	57.59	
CGR5883	6	61.14	
CICR0824	6	64.58	
HAU2822	6	65.27	
NAU5433	6	66.09	Yes
PGML03066	6	66.66	
HAU0210	6	67.98	
NAU2968	6	68.79	
NAU3803b	6	69.02	
NAU4946	6	69.47	Yes
BNL3650	6	70.32	
DPL0257b	6	72.60	
PGML03094	6	73.06	
C2-0139	6	73.40	
DPL0613	6	77.19	Yes
DPL0705a	6	90.01	Yes
SWU0473	7	0.00	Yes
DPL0364b	7	5.34	

CGR5119	7	8.61	Yes
PGML04198	7	22.72	Yes
NAU4030	7	29.18	Yes
NAU3581	7	30.79	
HAU1279	7	31.01	
SHIN-1585	7	33.38	
NAU5303	7	33.82	
HAU1367	7	38.10	Yes
NAU3654	7	38.32	
NAU5152	7	43.82	
SHIN-1405a	7	46.44	
SWU1338	7	49.87	
SHIN-0376a	7	52.71	
PGML01950	7	53.19	Yes
CGR5001a	7	53.86	
HAU3319	7	54.08	
CGR6512	7	55.73	
NAU2886	7	65.15	
COT048b	7	70.92	
CGR5372	7	71.87	
MUSS063	7	72.31	
NAU5120b	7	73.69	Yes
DPL0136	7	74.82	
CGR6894b	7	75.49	
NAU1048	7	77.11	Yes
CICR0375	7	80.82	
CICR0226	7	83.44	
NAU2002	7	85.06	Yes
COT096a	7	87.42	
CGR6381	7	88.09	
NAU3028	7	88.54	
NAU1085	7	92.24	Yes
HAU2346	7	92.46	
CICR0651	7	92.68	
HAU1763	7	92.90	
HAU3273	7	93.13	
HAU1483	7	93.35	
DC40253	7	102.67	Yes
HAU1754	7	109.34	Yes
CGR5691	7	111.21	
CGR6061	7	111.21	
HAU1780	7	113.32	
CIR141	7	115.18	Yes
CICR0224	7	115.86	
CICR0287	7	117.23	
BNL1694	7	117.90	
CM060	7	118.12	
CICR0680	7	118.34	
CGR5080	7	119.71	
TMB2844	7	120.16	
MUSB0691	7	121.06	
PGML04271a	7	124.21	
TMB0561	7	129.14	Yes
PGML03933	7	148.04	Yes
DPL0661	7	165.08	Yes
DC20152	7	174.65	Yes
CGR6815	7	175.79	
NAU6202	7	175.79	
NAU5458	7	176.46	
HAU3298	7	178.08	
MUSB0441	7	184.91	Yes

Gh506	7	190.40	Yes
CICR0399	7	190.85	
PGML03165a	7	191.38	
DPL0652	7	192.19	
HAU0597	7	193.56	
DPL0920b	7	194.23	
HAU1172	7	196.09	Yes
DC30150	7	196.54	
Gh548	7	197.91	
CGR5138	7	206.15	
NAU4082	7	207.31	
NAU2308	7	208.21	Yes
PGML03114	7	209.58	
PGML01916	7	212.76	Yes
NAU3324a	8	0.00	Yes
HAU1899	8	5.63	Yes
DPL0749	8	6.73	
SHIN-0153	8	13.94	
HAU1782a	8	16.29	Yes
CGR5759	8	17.85	
NAU1209	8	23.49	Yes
PGML01348	8	23.71	
HAU1308	8	23.71	
CICR0492	8	24.14	
NAU2293	8	24.14	
NAU0839	8	24.14	
HAU1607	8	25.01	
BNL3627b	8	25.92	
DPL0841a	8	30.90	Yes
DPL0111	8	36.14	
CGR5145	8	38.52	
HAU1390	8	38.94	Yes
PGML03354	8	39.59	
CGR6748	8	40.46	
DPL0154	8	41.79	
CGR5359	8	45.10	
DPL0862	8	49.54	
HAU1749	8	54.56	
CGR5537	8	56.12	
PGML0270	8	56.99	
TMB0029	8	57.53	
HAU1547	8	58.11	Yes
DPL0589	8	61.91	Yes
CGR5161	8	65.23	
HAU0709	8	65.23	
HAU1632	8	65.23	
PGML01704	8	66.10	
HAU3346a	8	67.42	
MUCS148	8	67.42	
NAU6213	8	67.85	
BNL3257	8	68.50	
BNL3792	8	68.71	
Gh198b	8	69.81	
CER0152b	8	71.64	Yes
CER0152c	8	77.66	
HAU2086a	8	92.72	Yes
NAU4978	8	105.83	
Gh634	8	107.16	
HAU1773	8	108.96	Yes
TMB1190	8	110.71	Yes
CER0099	8	111.43	

CICR0542	8	112.75	
NAU1164	8	112.75	
TMB2107	8	112.75	
CGR5363	8	112.97	
CGR5311	8	113.61	
HAU2832	8	114.71	
HAU3211	8	116.51	
MUSB0780	8	117.16	
DPL0861	8	118.26	
HAU2015a	8	120.55	Yes
STV028	8	139.83	Yes
HAU2319	8	152.12	
NAU4934	8	155.17	
DC40028	8	157.60	
CER0029	8	158.80	Yes
DPL0214a	8	160.84	Yes
HAU0520	8	163.63	
CICR0953b	8	164.96	
NAU3201b	8	166.52	
BNL3255	8	167.17	
COT065	8	167.17	
HAU3003	8	167.17	
HAU1739	8	167.81	
PGML04154b	8	169.14	
JESPR092	8	178.30	
CGR5172	8	184.75	Yes
NAU0779	8	186.49	
NAU2407	8	186.97	
NAU1017	8	187.84	
CGR5130	8	189.88	Yes
PGML03602	8	193.46	
JESPR232	8	195.51	
HAU1181	8	195.94	Yes
PGML00102	8	196.59	
DPL0353	8	203.52	Yes
SHIN-1494a	8	209.79	Yes
CGR5521	8	210.43	
DPL0152b	8	216.70	
DPL0238	9	0.00	Yes
NAU5468	9	5.14	Yes
BNL0686b	9	6.05	
HAU3052	9	6.62	
HAU2496	9	8.59	
NAU3888b	9	9.22	Yes
NAU3538	9	10.06	
Gh486	9	12.04	
CGR6170	9	19.08	
NAU6418	9	21.31	
PGML02974	9	24.03	Yes
NAU3101	9	26.24	
DPL0679	9	29.19	Yes
PGML03817	9	34.70	
BNL1162	9	37.35	
PGML04210	9	38.64	
NAU2575	9	39.26	Yes
Gh098	9	39.26	
DPL0697b	9	41.29	
DPL0783	9	49.20	
MUSS432	9	57.94	Yes
MUSB0009	9	61.14	
Gh584	9	61.77	

Gh111	9	61.97	
NAU5017	9	61.97	
Gh112	9	69.55	Yes
DPL0171	9	85.29	Yes
NAU0817	9	95.73	Yes
TMB0184	9	97.18	
HAU0792	9	98.60	
HAU2810	9	104.35	
BNL3779	9	109.40	
HAU2730b	9	109.89	
BNL1672	9	112.56	
BNL3031	9	113.18	
HAU1966	9	114.52	Yes
CICR0334	9	116.26	
STV177	9	117.10	
HAU0361	9	130.53	
PGML01868c	9	132.79	Yes
NAU0805	9	140.49	Yes
HAU3218b	9	157.89	Yes
Gh495	9	167.15	
CGR6252a	9	169.60	
CGR6921	9	171.25	
CGR6072	9	171.75	
COT151	9	172.16	
NAU2211	9	173.67	
BNL3582	9	174.41	Yes
DPL0850	9	174.93	
CGR5128a	9	175.77	
CGR5110	9	176.83	
CGR6719	9	177.46	
MUCS426	9	180.32	Yes
MUSS397	9	181.94	
Gh247	9	182.78	
BNL1317	9	183.04	
CGR5707	9	184.04	
NAU0882	9	184.04	
SHIN-0347	9	184.46	
NAU0877	9	185.31	
HAU3150	9	185.94	
CM071	9	187.45	
BNL1414	9	187.86	Yes
SWU0124	9	188.07	
BNL1030	9	188.41	
NAU3365	9	189.11	
HAU1617	9	189.53	
CGR5426	9	190.59	
BNL2590	9	192.06	
HAU1683	9	193.17	Yes
HAU0455	9	193.17	
CGR5292	9	194.04	
HAU0085	9	199.14	
DPL0524a	9	200.88	
PGML02558	9	203.58	
NAU2354	9	203.99	Yes
DPL0514b	9	206.96	Yes
DPL0317	10	0.00	Yes
HAU0955	10	2.21	
PGML04510	10	5.63	
CICR0688	10	9.69	
CICR0002	10	9.69	
DPL0533	10	10.16	

STV031	10	10.79	Yes
CGR5565b	10	10.94	
CGR5399	10	10.94	
SWU1312	10	14.34	
DPL0297	10	24.36	
NAU5166	10	27.98	Yes
CICR0886a	10	31.82	
CGR5406	10	31.97	
CICR0194	10	32.13	
NAU1066a	10	32.78	Yes
HAU2873b	10	35.95	Yes
DC20161	10	36.42	
NAU2271	10	36.42	
HAU0635	10	36.57	
PGML03513	10	37.53	
BNL1160	10	38.49	
PGML00058	10	39.28	
SHIN-0087a	10	40.41	Yes
HAU1423a	10	40.71	
JESPR056a	10	41.14	
DPL0485	10	41.84	
DPL0149	10	50.67	Yes
NAU1066b	10	64.57	Yes
Gh236	10	73.00	
DPL0310	10	81.52	
TMB1288	10	85.09	Yes
BNL1665	10	85.27	
PGML04611b	10	85.59	
DC40119	10	86.06	
CGR5717	10	86.21	
DPL0553	10	86.84	
DC40188	10	88.67	
DPL0468	10	91.66	Yes
NAU6393	10	92.62	
NAU3682	10	94.09	
CM027	10	95.39	
TMB0380	10	96.09	
DPL0108	10	102.57	Yes
SHIN-0783	10	111.24	
CGR5624	10	118.35	
CGR6839	10	119.48	
NAU0921	10	121.31	Yes
HAU2009	10	122.11	
BNL1161	10	122.42	
CGR5873	10	122.42	
TMB0307	10	122.49	
SWU1259b	10	122.57	
DPL0165	10	125.76	Yes
DPL0116a	10	132.16	
NAU2869	10	146.68	Yes
HAU0230a	10	154.52	
PGML00696b	10	157.72	Yes
NAU4910	10	159.55	
CGR6818	10	161.03	
CIR166	10	164.49	
HAU1516b	10	169.85	Yes
C2-0133a	10	176.46	
BNL3563	10	178.11	Yes
HAU0949	10	178.42	
NAU1182	10	180.82	
NAU4008	10	181.77	

NAU2082	10	182.24	
PGML04401	10	182.71	
MUSB0698	10	183.02	
NAU2911	10	183.49	Yes
NAU2991	10	188.25	Yes
PGML03148	10	194.88	
HAU1701	10	199.66	Yes
Gh283	10	200.13	
COT119	10	200.44	
NAU5323	10	201.91	
CGR5349b	10	202.38	
Gh058	10	203.51	Yes
HAU0218b	10	205.92	
CICR0943b	10	213.73	
CGR5040	10	214.56	Yes
PGML01392	10	218.37	
PGML04583	10	218.86	
CICR0897	10	221.25	Yes
CICR0943a	10	223.45	
HAU2824	10	224.41	Yes
CICR0577	10	224.88	
SHIN-0613	10	224.88	
SWU0159	10	224.88	
HAU2147	10	225.34	
NAU2508	10	231.96	Yes
DPL0617	11	0.00	Yes
Gh433	11	3.17	Yes
PGML04039a	11	12.78	
HAU2974	11	21.99	Yes
CICR0499	11	24.02	
NAU3074	11	32.85	Yes
MUSS281	11	56.31	Yes
CGR5113b	11	60.74	
DPL0715	11	60.74	
NAU2257	11	60.74	
HAU1134	11	60.93	Yes
NAU5461b	11	66.18	
NAU5461a	11	69.59	Yes
CICR0283	11	71.62	
NAU5192	11	71.99	
NAU3621	11	72.76	
HAU3031	11	73.94	
BNL3442	11	77.15	
CICR0044	11	79.60	
CGR5578	11	85.45	
CGR6697b	11	86.66	Yes
HAU0848	11	90.27	Yes
NAU3317	11	92.30	
STV069	11	95.95	
HAU1430	11	97.97	Yes
DPL0472	11	99.57	
PGML00528	11	100.34	
DPL0900	11	101.51	
CGR6476	11	102.36	
NAU3409	11	103.87	Yes
BNL1151	11	108.57	Yes
NAU3265	11	109.95	
CGR5354	11	111.33	
PGML04105a	11	112.51	
DPL0698	11	113.08	
TMB2281	11	113.65	

BNL3431	11	113.92	
TMB0043	11	115.86	
PGML04308	11	117.24	
CICR0602	11	117.62	
CICR0361	11	119.42	
TMB2803	11	122.35	
BNL1404	11	122.54	
TMB1915	11	122.54	
HAU2478	11	122.92	
JESPR135	11	123.17	
BNL3411	11	123.78	
Gh074b	11	124.65	Yes
NAU3234	11	126.68	Yes
MUSS244	11	130.84	Yes
CER0035	11	131.82	
SHIN-0224	11	132.59	
CICR0359	11	133.56	
CICR0951	11	133.94	
CICR0286	11	134.32	
CICR0246	11	134.70	
NAU2651	11	135.27	Yes
CICR0433	11	136.65	
DPL0789	11	138.67	
PGML04039b	11	141.37	
DPL0528	11	146.91	Yes
SHIN-0610	11	147.88	
HAU2837	11	150.34	
NAU3478	11	153.28	Yes
PGML03001	11	154.05	
DPL0065	11	157.71	
CGR6862	11	162.15	
DPL0851	11	163.33	
HAU2836b	11	164.92	
BNL0625	11	166.09	Yes
TMB2453	11	166.86	
C2-0078	11	167.24	
DC40196	11	167.62	
DPL0253	11	168.59	
SHIN-0601	11	169.68	
DPL0505	11	170.53	
COT091	11	173.23	
TMB0064	11	175.23	Yes
PGML03769	11	181.27	
HAU0639	11	186.52	
CGR5835	11	187.30	
HAU0205	11	187.48	Yes
CGR6385	11	187.48	
TMB0426	11	188.10	
DPL0103	11	193.59	Yes
HAU0217	11	198.77	
DPL0021	11	199.80	
SWU0470	11	203.61	
NAU3227	11	207.68	Yes
CGR5112	11	208.25	
BNL1408	11	208.44	
MUCS507	11	210.25	
MUSB0827	11	211.02	
DPL0338	11	211.20	
CICR0280	11	213.01	
NAU0891	11	216.13	
NAU3703	11	216.74	

DPL0325	11	217.12	Yes
HAU0512	11	217.12	
DPL0571	11	223.32	
JESPR296	11	231.97	Yes
CGR6270	11	236.59	
NAU4086	11	237.97	Yes
NAU4962	11	238.74	
DPL0270	11	239.12	
HAU3288	11	248.24	
DPL0209	11	252.99	Yes
CM140	11	258.82	
MUCS379	11	259.99	Yes
CER0153b	11	261.58	
CGR5428	11	262.96	
C2-0120	11	264.99	
MUCS609	11	266.37	Yes
HAU1283b	11	275.58	Yes
HAU1809b	11	275.77	
CER0098	11	275.96	
CM050	12	0.00	
TMB0799	12	12.62	Yes
NAU5252	12	13.95	
NAU3561	12	15.03	
C2-0111	12	15.64	
NAU3862	12	16.72	Yes
HAU3127	12	20.20	
DC40186	12	23.28	
HAU2934	12	23.58	
NAU4926	12	24.34	
CGR5111	12	25.93	
CGR5658	12	26.85	
CICR0393	12	27.46	
DPL0248	12	28.38	Yes
BNL3599a	12	31.45	Yes
CGR6698a	12	31.45	
DPL0036a	12	31.75	
DC20022	12	32.87	
PGML00640	12	35.98	
PGML04005	12	42.81	
CGR6826	12	44.77	
NAU0884	12	44.82	Yes
DPL0575	12	45.22	
HAU3323	12	46.98	
PGML01594b	12	49.81	
MUCS363	12	51.10	
CGR6844	12	52.52	
PGML02980	12	53.77	
DPL0796a	12	54.22	
HAU1313	12	54.52	
CGR6764	12	54.82	
DPL0866a	12	56.58	
PMGL00420	12	59.66	
HAU2881	12	63.56	Yes
CICR0413	12	65.33	
CGR5517	12	67.63	
CGR6056	12	67.78	
CGR6825	12	67.78	
NAU2176	12	68.38	Yes
CICR0469	12	68.53	
HAU1434b	12	69.58	
NAU5164b	12	69.91	

HAU1666	12	70.11	
COT135	12	70.51	
NAU5139	12	72.93	Yes
CGR6742a	12	74.34	
CICR0324	12	75.44	
Gh243b	12	78.01	
TMB0263	12	79.95	
BNL1673	12	80.71	
DPL0303	12	80.71	
DPL0379	12	81.72	
DPL0380a	12	82.88	
CICR0199	12	83.70	
HAU1361	12	84.57	Yes
NAU0943	12	85.02	
HAU1874	12	85.62	
CGR5158	12	86.07	
DPL0491	12	93.32	
HAU0734	12	101.52	Yes
DPL0400	12	105.65	
HAU0107	12	107.77	
Gh188	12	108.37	
NAU4889	12	110.86	Yes
NAU3713	12	111.47	
PGML01637	12	113.41	
CGR5151	12	114.66	
HAU1454	12	114.96	Yes
NAU2672	12	118.24	
HAU0989	12	119.69	Yes
CGR5102	12	123.55	
CICR0389	12	123.85	
HAU1321	12	124.30	
HAU0295	12	125.39	
NAU1301	12	126.54	
DPL0917a	12	128.23	Yes
BNL4059	12	130.17	
DPL0443	12	130.94	
NAU3778	12	132.91	
DC40230	12	133.46	
DPL0343	12	137.96	Yes
HAU0371	13	0.00	Yes
CGR6812	13	1.77	
NAU0803	13	2.00	Yes
HAU1577	13	2.49	
HAU0250	13	8.31	
BNL1707	13	8.68	Yes
CIR054	13	13.94	Yes
PGML02505a	13	19.76	
PGML03773	13	27.46	Yes
NAU2730	13	32.18	Yes
DPL0639	13	34.10	
CICR0628b	13	36.45	
NAU3948	13	40.66	Yes
BNL1495	13	41.07	
NAU5279	13	44.19	
BNL2652a	13	45.13	Yes
DPL0754	13	45.35	
CICR0382	13	47.28	
BNL2449	13	48.39	
CGR5786b	13	49.12	
DPL0274	13	49.71	
Gh092a	13	52.01	Yes

Gh092b	13	53.52	
Gh678	13	58.51	Yes
BNL1438	13	62.47	
PGML03194b	13	65.71	
DC40441	13	68.76	
CGR5925b	13	69.60	
PGML03887b	13	70.57	
CM063a	13	71.49	
TMB0403	13	71.49	
DPL0460b	13	71.89	
NAU3989	13	73.36	Yes
HAU1091	13	73.36	
DC20120	13	74.09	
MUSB0421	13	76.65	
PGML04464	13	79.00	
NAU1201	13	82.25	Yes
MGHES38b	13	83.26	
HAU2456	13	84.69	
HAU1513b	13	86.20	
DPL0635	13	86.38	
CGR6231b	13	90.10	
Gh215	13	103.25	Yes
PGML02035	13	115.68	Yes
HAU1908	13	131.76	
NAU3398	13	137.04	Yes
NAU3522	13	151.30	Yes
PGML00806	13	158.73	
CGR5242	13	164.25	Yes
CER0005	13	165.56	
CGR5827	13	165.56	
DPL0083	13	165.56	
Gh034	13	165.56	
Gh592	13	165.92	
PGML01230	13	166.65	
HAU2089	13	170.86	
PGML04131a	13	179.61	Yes
NAU3468	13	184.33	Yes
CICR0182	13	185.06	
SHIN-0412	13	185.24	
CGR5021b	13	185.60	
MUCS145	13	186.52	Yes
HAU1195	13	187.64	
HAU1997	13	188.00	
CGR5331	13	188.74	
CICR0273	13	188.74	
SHIN-1462	13	188.74	
NAU2300	13	189.10	
CGR5554	13	192.88	Yes
SHIN-1512	13	206.74	
CICR0675	13	211.73	
CGR5005	13	211.91	
HAU2836a	13	212.46	Yes
CGR5544	14	0.00	Yes
CGR5668	14	8.43	Yes
PGML03546	14	14.15	
DPL0919	14	14.54	
HAU3071	14	17.07	
NAU3733	14	18.27	
NAU3585	14	18.46	Yes
PGML02861b	14	18.46	
HAU0517	14	20.31	

NAU5047	14	22.15	
HAU3236	14	22.35	
NAU5467	14	22.54	
HAU1485	14	23.53	Yes
NAU5490	14	24.95	
PGML01915	14	31.95	
NAU3648	14	40.73	Yes
PGML03485	14	43.27	
CGR5876b	14	44.47	
NAU5421	14	46.54	Yes
CICR0140	14	47.13	
PGML03232	14	48.54	
HAU1980a	14	50.61	Yes
PGML02878	14	50.61	
NAU3485	14	50.61	
NAU4024	14	52.24	
CGR5448	14	55.01	
CICR0842	14	58.16	
HAU1049	14	60.10	
PGML02670	14	60.49	
NAU5250	14	61.28	
TMB1348	14	62.37	
CGR6683	14	63.48	
CGR6707	14	68.60	Yes
NAU3499	14	77.67	
C2-0079	14	81.62	
BNL1059	14	83.01	Yes
CGR6550	14	86.36	
PGML02953b	14	89.61	
CICR0377	14	94.42	Yes
CICR0213	14	96.94	
CICR0135	14	99.47	
CGR6729b	14	101.32	
NAU2154	14	101.32	
NAU4025	14	101.32	
DPL0235	14	101.71	
CGR6716b	14	102.09	
CICR0592	14	102.09	
NAU2155	14	102.09	
NAU3312	14	102.09	
NAU3816b	14	102.09	
SHIN-1481b	14	102.29	
Gh462	14	102.48	
BNL3145	14	104.33	Yes
CGR5818	14	106.41	
PGML03980	14	106.79	
CGR6383	14	113.15	Yes
NAU4929	14	126.96	Yes
Gh051	14	131.79	
BNL2882	14	132.38	
CGR5581	14	132.97	
TMB0803	14	133.36	
CGR5030	14	133.94	
NAU2987	14	135.57	Yes
HAU1888	14	135.76	
PGML04271b	14	136.15	
CGR6948	14	138.24	
PGML04653	14	139.87	
DPL0638	14	140.46	
PGML03030	14	141.45	
HAU0438	14	143.08	Yes

PGML01010	14	146.50	
PGML01521	14	148.19	Yes
BNL3034	14	150.04	
MGHES73	14	150.43	
NAU2336	14	150.43	
NAU3913	14	150.43	
HAU1741	14	150.62	
NAU3242	14	155.17	
PGML02865	14	160.56	
PGML03864	14	161.76	
DPL0354	14	164.76	
DPL0390	14	168.83	
NAU3225	14	171.34	Yes
PGML02080	14	172.13	
CGR5258	14	172.52	
PGML00357	14	174.81	
SHIN-0659b	14	178.14	
PGML01568	14	179.93	
NAU3214	14	181.56	Yes
NAU5465	14	184.56	
HAU1720	14	189.65	
CICR0052	14	190.85	
HAU1123	14	190.85	
NAU3820	14	191.24	Yes
DPL0502a	14	194.00	Yes
CER0138	14	195.42	
CER0101	14	199.96	
HAU0883a	14	202.97	Yes
DC40046	14	205.50	
DPL0473	14	206.50	Yes
HAU1219a	14	206.88	
BNL3932	14	206.88	
DPL0521	14	207.27	
HAU1219b	14	215.94	Yes
NAU4073	15	0.00	Yes
NAU3102	15	4.06	
CICR0815	15	16.32	Yes
HAU1045	15	19.51	Yes
NAU3347	15	24.51	Yes
HAU1058a	15	26.90	Yes
CGR5106a	15	27.06	
CGR6129	15	33.97	
PGML00834	15	39.76	
NAU3177	15	43.61	Yes
CGR5834	15	43.91	
HAU0489	15	44.22	
HAU3050a	15	47.35	
PGML03798	15	51.56	
DC40217	15	52.69	
HAU1001	15	53.64	
SWU1087	15	54.27	
HAU1354	15	54.58	
NAU3714	15	55.21	Yes
NAU3057	15	58.40	
PGML00799	15	59.03	
PGML02554	15	60.42	
TMB0119b	15	61.46	
TMB1224b	15	61.46	
BNL0786	15	62.25	Yes
HAU0670	15	66.78	Yes
DPL0023a	15	68.98	

HAU1427	15	70.28	
PGML03654	15	70.28	
NAU3744a	15	70.44	
HAU0309	15	71.06	
DPL0326	15	72.19	
DPL0109b	15	72.82	
CICR0455	15	73.45	
CGR5236	15	74.92	
HAU0271	15	76.22	
Gh649a	15	77.34	Yes
DPL0425	15	80.96	
DPL0428	15	84.16	
NAU2985	15	88.22	Yes
MUSS422	15	97.84	
TMB0375	15	101.01	
CGR6726	15	101.18	
HAU3351	15	101.55	
MUSB0440	15	102.28	
PGML04991a	15	102.43	
BNL4082	15	103.08	Yes
DPL0509b	15	103.24	
MUSB1130	15	107.96	
DPL0264	15	111.57	
PGML02703	15	111.57	
DC30026	15	112.04	Yes
PGML04427	15	112.35	
TMB0585	15	112.98	
PGML04032	15	116.40	
MUSB1081	15	120.04	Yes
NAU3486	15	123.88	Yes
DPL0300	15	130.78	
DPL0187	15	136.86	
DC40014	15	140.07	
JESPR180	15	140.23	
JESPR205	15	140.23	
JESPR298	15	140.23	
DPL0509a	15	140.39	
CICR0665	15	140.54	
CICR0805	15	140.54	
NAU5230	15	140.69	
BNL1666	15	140.85	
NAU3680	15	140.85	
NAU0861	15	142.15	
PGML02493	15	142.77	
DPL0437	15	142.93	
NAU3690	15	143.08	Yes
PGML01203	15	143.08	
PGML02985	15	143.08	
C2-0022C	15	143.55	
TMB0201	15	145.02	
NAU3736	15	145.49	
TMB1660	15	145.64	
TMB1152a	15	145.80	
COT059	15	148.19	
TMB1664	15	151.49	
PGML02824b	15	154.45	
CGR5056a	15	160.73	Yes
CGR6803b	15	162.43	
HAU2129	15	163.06	
CER0013	15	165.84	
CGR5326a	15	165.99	

CGR5663	15	167.12	
DPL0346a	15	170.58	
DPL0402	15	173.82	
JESPR152	15	174.48	Yes
MUSS085	15	177.43	Yes
HAU3132	15	179.08	
SWU0280b	15	179.87	
MUCS141	15	182.27	
MUCS422	15	183.40	
MUCS410	15	184.36	
HAU0059	15	184.83	Yes
HAU0077	15	185.46	
NAU5138	15	187.60	
DPL0302	15	188.87	
MUSS563	15	195.09	Yes
NAU3496	16	0.00	Yes
NAU3459	16	7.10	Yes
PGML04451	16	8.81	
CGR5679	16	10.52	
CIR175	16	11.15	Yes
DPL0364a	16	12.27	
CER0112	16	14.31	
CGR5149	16	16.79	Yes
CGR5396	16	19.04	
DPL0829	16	22.22	
NAU2239	16	27.21	
PGML02837	16	28.80	
HAU0120	16	29.18	Yes
SHIN-1405b	16	29.95	
DPL0501	16	30.53	
CICR0442	16	31.50	
HAU0732	16	32.89	
CGR5001b	16	34.44	
SHIN-0376b	16	36.31	
PGML02869	16	38.79	
PGML04674	16	44.95	
NAU5120a	16	52.72	Yes
CGR5621	16	58.09	
CGR6894a	16	59.27	
COT048a	16	59.74	
NAU4956	16	61.45	
PGML01349	16	62.23	
HAU1836	16	63.61	
Gh056	16	64.59	
BNL2634	16	65.97	Yes
CICR0587	16	66.35	
PGML02608	16	66.54	
HAU1201	16	67.51	
HAU2555	16	68.09	
PGML04077	16	69.06	
DC40065	16	69.64	
DPL0511	16	70.82	
C2-0011B	16	71.79	
PGML03471	16	73.60	
NAU2749	16	77.27	
NAU5408	16	79.08	Yes
NAU3594	16	79.85	
PGML02570	16	81.03	
BNL3008	16	81.41	
JESPR102	16	81.60	
NAU6259	16	82.99	

DPL0432	16	85.94	
CICR0084	16	88.82	
COT096b	16	90.29	
NAU2186	16	90.29	
CIR100	16	90.67	
CGR6280	16	92.06	
PGML01011	16	93.87	
PGML02429	16	95.68	
BNL2986	16	95.91	Yes
STV023	16	98.11	
PGML02824a	16	102.48	
DPL0167	16	111.73	Yes
PGML00586	16	124.36	
DPL0342	16	127.78	Yes
CGR6686	16	127.97	
TMB0837	16	129.04	
NAU2995	16	130.13	Yes
PGML01330	16	135.13	
DPL0294	16	140.17	
CGR5139	16	141.14	
NAU6664	16	143.17	
NAU6375	16	143.94	
DPL0048	16	145.75	Yes
Gh002	16	148.00	
TMB0180	16	148.00	
DPL0897	16	148.98	
PGML03165b	16	150.36	
JESPR297	16	151.75	Yes
PGML01309	16	151.75	
PGML00820	16	151.98	
DPL0920a	16	152.91	
NAU0797	16	153.09	
CGR5018	16	154.07	
BNL1604	16	158.26	
CGR5880	16	158.26	
HAU1399	16	165.37	Yes
HAU1559	16	171.52	
CICR0774	16	172.70	
NAU2680	16	172.70	
NAU2733	16	172.89	Yes
NAU2734	16	173.08	
CICR0452	16	174.05	
NAU2556	16	175.65	
BNL3065	16	176.71	Yes
DPL0283	16	182.47	Yes
CGR6680	16	188.28	
PGML00300	16	189.28	
NAU3906	16	189.66	
HAU2060	16	190.04	
CICR0027	16	191.43	Yes
NAU5325	16	192.00	
NAU3678	16	192.38	
DPL0492a	16	192.76	
DPL0492c	16	197.75	
DPL0603	16	210.52	Yes
CICR0447	17	0.00	Yes
NAU2898	17	2.04	Yes
MGHES17	17	2.04	
CGR6905	17	2.68	
PGML03126	17	4.45	
HAU0800a	17	5.31	Yes

DC40292	17	12.81	
PGML02308	17	13.27	
CGR6834	17	13.91	
DPL0017	17	14.12	
TMB1268	17	14.79	Yes
DPL0245b	17	17.76	
CICR0004	17	21.56	
HAU2014	17	23.34	Yes
HAU3318	17	23.76	
NAU3349	17	27.84	
DPL0281	17	32.48	Yes
PGML02856	17	37.13	
JESPR101b	17	38.43	
PGML04991b	17	39.29	
PGML04550	17	40.15	
BNL3955	17	40.90	
HAU1024	17	41.65	
HAU1513c	17	42.51	
DPL0200	17	45.01	
NAU2909	17	47.26	Yes
CGR6695	17	49.52	
PGML01532	17	51.77	
PGML01494	17	52.19	
COT064	17	52.83	
NAU2325	17	56.36	Yes
NAU6634	17	67.53	
NAU3309	17	82.35	Yes
NAU6542	17	90.26	
HAU0764	17	93.28	Yes
CICR0331	17	94.13	
JESPR195	17	94.88	
DPL0279	17	97.01	
PGML04756	17	106.45	Yes
NAU4052	17	114.34	
DPL0529	17	114.55	
PGML04142	17	114.55	
HAU0863	17	115.63	
NAU2150	17	117.64	
CGR6185	17	119.41	
NAU3800	17	119.83	
HAU0630	17	120.04	Yes
HAU0195a	17	122.79	Yes
HAU2786	17	127.44	
HAU1413	17	128.08	Yes
NAU3995	17	128.08	
NAU1167	17	129.39	
NAU3839	17	131.40	
NAU2742	17	132.94	
PGML00950a	17	134.71	
NAU3016b	17	136.96	
TMB0874	17	138.89	Yes
BNL2496	17	139.83	
SHIN-1343	17	140.69	
CGR6830	17	141.11	
CGR5300	17	142.19	
NAU3797	17	143.73	
NAU2691	17	143.94	Yes
PGML00880	17	145.04	
NAU6173	18	0.00	
NAU3685	18	5.04	Yes
PGML02505b	18	9.99	

HAU0595	18	10.85	Yes
BNL2544	18	11.37	
DPL0286	18	11.70	
CER0122	18	11.70	
HAU1880	18	12.22	
NAU3203	18	13.83	Yes
PGML02477	18	18.12	
Gh092c	18	28.97	Yes
NAU4103	18	38.67	Yes
CGR6787	18	42.63	
DPL0421	18	44.24	
BNL2652b	18	44.93	
DPL0033	18	45.79	
CGR5786a	18	46.30	Yes
PGML01649	18	47.50	
SHIN-1530	18	47.70	
DC20021	18	47.70	
CM063b	18	47.70	
DPL0460a	18	48.04	
PGML03887a	18	50.04	
BNL1079	18	51.10	Yes
BNL1721	18	51.79	
NAU3816a	18	52.13	
CGR5856	18	53.74	
HAU3061	18	55.94	
PGML00374	18	66.07	Yes
NAU1141	18	77.58	Yes
PGML02492	18	89.51	
PGML04767	18	95.53	
NAU3211	18	95.87	Yes
CGR6231a	18	96.04	
JESPR153	18	96.04	
BNL3479	18	96.56	
DC40426	18	97.25	
COT114	18	98.48	
PMGL00427	18	100.09	
DPL0161	18	103.58	
DPL0660	18	105.99	
DPL0420	18	107.04	
CGR6522a	18	107.21	
NAU2443	18	107.90	
SHIN-1346	18	108.58	
TMB1767	18	113.02	
SHIN-1451	18	117.46	Yes
DPL0339	18	118.52	
DC40005	18	120.52	
CER0145	18	121.03	
HAU1036	18	121.54	
JESPR056c	18	122.96	
Gh055	18	123.13	
NAU3017	18	123.64	Yes
NAU3589	18	125.45	
HAU1381	18	126.50	
SHIN-1403	18	126.50	
PGML04131b	18	127.73	
CGR5021a	18	129.73	
NAU5074	18	130.96	Yes
SWU0126	18	132.19	
HAU1212	18	132.36	
HAU1989	18	132.36	
C2-0104	18	132.53	

HAU3406	18	134.54	
DPL0795	18	137.38	Yes
DPL0348	18	138.80	Yes
PGML04888	18	138.96	
DPL0375	18	141.37	
NAU3161	18	143.37	
HAU1167	18	143.54	
NAU3080	18	144.05	
NAU2488	18	144.22	
HAU0899	18	144.56	
CGR6750	18	144.73	
HAU0209	18	145.24	
CICR0223	18	146.29	
HAU2550	18	148.90	
NAU4860	18	152.52	Yes
NAU3843	18	156.62	
PGML01307	18	161.83	
NAU3991	18	164.45	
HAU1922	18	166.64	
NAU3447	18	166.82	
HAU1782b	18	167.68	
PGML03537	18	168.55	
NAU3827	18	168.72	Yes
DPL0910	18	169.23	
CICR0227	18	170.59	
NAU2863	18	175.10	Yes
PGML02956	18	186.95	Yes
CICR0851	19	0.00	Yes
HAU0112	19	4.14	
CICR0547	19	7.36	
PGML02014	19	11.50	
Gh182	19	13.57	Yes
NAU3631	19	14.32	
DPL0247b	19	14.32	
NAU3609	19	14.32	
HAU0139	19	14.32	
HAU3114	19	16.12	
NAU3405	19	17.39	Yes
PGML01008	19	19.74	
CGR6151	19	21.78	
DPL0140	19	25.66	
CGR6708b	19	26.66	
BNL3452	19	27.41	Yes
PGML04350b	19	29.77	
NAU2894	19	33.60	
HAU3069	19	39.04	
SHIN-1398	19	39.79	
NAU4884	19	41.59	Yes
CGR5799	19	44.52	
CGR5803	19	45.01	
DPL0076	19	45.51	
DPL0870	19	45.75	
CGR5814	19	45.75	
NAU3372	19	48.39	Yes
TMB1599	19	50.42	
NAU3343	19	54.93	
COT037	19	60.61	Yes
CIR212	19	63.64	
HAU0216	19	66.28	
Gh071	19	67.28	
CGR5845	19	68.55	

DPL0556	19	69.82	
CGR5510	19	70.57	
DC40130	19	70.57	
PGML04278	19	72.10	
DPL0169	19	73.37	Yes
BNL1690	19	74.52	
MUCS517	19	75.38	
HAU1653	19	77.46	
Gh229	19	79.42	
BNL0285	19	80.80	Yes
SWU0091	19	82.07	
CICR0065a	19	83.60	
HAU2783	19	84.10	
PGML04402	19	84.34	
NAU2380	19	85.88	
DPL0444	19	88.23	Yes
PGML03051	19	89.24	
PGML03375	19	89.24	
DPL0145	19	91.59	
BNL1064	19	99.97	Yes
CER0148	19	106.24	
BNL0852	19	106.48	
CIR139a	19	106.98	
CICR0183	19	107.47	
CICR0274	19	107.47	
CICR0609	19	107.47	
HAU0878	19	107.47	
MGHES70	19	107.47	
CICR0654	19	107.72	
NAU1221	19	108.21	
CGR5732	19	108.46	
CICR0203	19	108.46	
CICR0262	19	108.46	
CICR0292	19	108.46	
CICR0515	19	108.46	
NAU0792	19	108.46	
NAU0828	19	108.56	
CICR0710	19	108.70	
CICR0734	19	110.24	
CICR0616	19	111.77	
CICR0826	19	113.85	
DPL0540	19	116.48	Yes
Gh109	19	117.28	
NAU5330	19	123.02	
NAU3497	19	131.02	Yes
DPL0210a	19	137.52	
CGR6128	19	142.63	
CICR0906	19	142.88	
PGML00845	19	143.89	
BNL3903	19	145.03	
NAU5475	19	145.90	Yes
HAU1952	19	146.15	
NAU2959	19	146.15	
PGML03255	19	148.23	
HAU2616	19	149.23	
DPL0064	19	150.24	
NAU5299	19	152.88	
CIR062	19	153.37	
HAU1071b	19	153.62	Yes
NAU5005	19	154.62	
NAU6406	19	155.89	

NAU6619	19	156.14	
CICR0333	19	156.88	
DPL0215	19	161.66	Yes
CGR6250	19	166.63	
DPL0040a	19	169.97	Yes
PGML02574	19	170.22	
DPL0155b	19	173.75	
TMB0131a	19	178.16	
NAU3698	19	178.69	
CER0090	19	178.94	
DPL0192	19	179.43	
BNL1878	19	179.68	Yes
DPL0143	19	179.68	
TMB1282	19	179.68	
CGR6088	19	182.32	
HAU1385b	19	185.54	
NAU5489	19	189.99	Yes
DPL0174a	19	192.07	
NAU0986	19	196.20	
HAU0470	19	196.45	
NAU2274	19	196.95	Yes
CGR6835	19	196.95	
NAU3096	19	196.95	
Gh354	19	202.73	Yes
PGML00967	19	204.53	
PGML01057	19	205.80	
PGML03194a	19	211.58	
DPL0908b	19	219.12	
NAU3652	19	221.30	Yes
SHIN-0685	19	222.31	
NAU2650	19	223.84	
PGML03762	19	225.64	
NAU6318	19	226.14	
DPL0163	19	226.38	
PGML04339	19	226.63	
NAU6466	19	230.45	
DPL0137b	19	235.23	
PGML04363	19	235.98	
Gh447	19	237.78	Yes
PGML04977	19	241.30	
BNL3535	19	242.78	Yes
BNL3347	19	245.20	
CM042	19	245.20	
DPL0788	19	245.20	
JESPR236	19	245.20	
DPL0271	19	248.13	
MUSS138	19	253.25	
NAU3110	19	253.50	
NAU1102	19	253.74	
PGML03410	19	256.10	
COT125	19	256.59	
HAU1785	19	257.09	Yes
CGR5126	19	259.73	Yes
PGML01289	19	261.26	
SHIN-0256	19	264.19	
TMB1600	19	264.69	Yes
TMB1437	19	264.69	
PGML00973	19	267.33	Yes
PGML03369b	19	269.40	
PGML03369a	19	278.22	Yes
DC40425	19	290.77	

BNL3089	19	292.56	Yes
HAU1400	19	294.12	
CGR5106b	20	0.00	Yes
HAU1058b	20	5.86	Yes
CGR5565a	20	12.33	Yes
HAU0156	20	14.69	
HAU1348	20	15.13	
PGML01062	20	15.35	
CIR094	20	16.10	
NAU5307	20	20.08	Yes
CICR0254	20	22.19	
NAU4071	20	23.56	Yes
HAU0773	20	26.18	Yes
CGR6040	20	26.18	
CICR0886b	20	28.28	
DPL0486	20	32.27	
PGML01943	20	34.04	
PGML03124	20	35.03	
DPL0394	20	37.91	
SWU1259a	20	38.36	
HAU2700	20	38.36	
CGR5237	20	41.78	Yes
HAU1969	20	46.35	
NAU4918	20	48.21	
HAU1423b	20	53.07	
SHIN-0087b	20	57.92	
NAU2698	20	58.38	
NAU3434	20	60.49	Yes
NAU4973	20	61.86	
HAU0019	20	62.31	
CICR0319	20	64.16	
DPL0037	20	66.75	
HAU0175	20	67.22	
HAU2753	20	68.12	
NAU5013	20	68.34	Yes
CICR1000	20	68.79	
COT027	20	71.15	
NAU6234	20	80.62	
PGML01142	20	88.43	
CGR5508	20	90.34	
NAU1005	20	90.34	
BNL3948	20	91.15	
CGR5740	20	91.68	Yes
Gh119	20	96.25	Yes
PGML03483	20	97.15	
NAU6365	20	97.82	
HAU2178	20	98.04	
MUCS511	20	98.26	
JESPR171	20	98.57	
PGML04611a	20	99.15	
PGML02370	20	99.37	
PGML04313	20	100.27	
NAU3574	20	104.25	
HAU1491b	20	113.72	Yes
HAU3201	20	121.61	Yes
HAU1491a	20	139.90	Yes
NAU3813b	20	152.59	Yes
TMB1831	20	159.69	
TMB1629	20	161.61	
CICR0068	20	162.31	
TMB1125	20	162.96	Yes

MUSS347	20	163.65	
NAU4928	20	164.32	
CGR6522b	20	164.54	
NAU3813a	20	164.76	
DPL0361	20	165.90	
PGML00696a	20	166.34	
PGML02892	20	170.33	
NAU3665	20	175.52	Yes
NAU6215	20	176.42	
BNL0119	20	176.64	
HAU0230b	20	180.06	
NAU2893	20	187.60	Yes
Gh048	20	194.00	Yes
C2-0133b	20	196.69	
DPL0442	20	200.39	Yes
HAU1670	20	202.50	
HAU1516a	20	202.72	
CGR6930b	20	204.33	
PGML03605	20	212.22	
HAU0218a	20	214.33	
NAU6448	20	215.46	
NAU6305	20	215.68	
NAU6449	20	215.68	
CGR5349a	20	220.86	
HAU2543	20	221.53	Yes
CGR6701	20	223.15	Yes
DC40266	20	227.72	Yes
CGR6110	20	228.16	
DPL0320	21	0.00	Yes
CICR0187	21	13.24	
CGR5113a	21	14.89	
NAU3377	21	14.89	
HAU1484	21	15.12	Yes
DPL0717	21	19.48	
CGR5747b	21	31.48	
CGR5747a	21	33.13	
SHIN-1579	21	33.82	Yes
CICR0717	21	40.37	Yes
HAU1018	21	43.68	Yes
DPL0522	21	44.60	
PGML01055	21	45.52	
CGR5097	21	48.74	
NAU4003	21	51.42	Yes
DPL0131	21	54.10	
CGR5217	21	57.89	Yes
DPL0582	21	60.04	
CGR6697a	21	60.73	
NAU3731	21	62.63	Yes
HAU1703	21	65.85	
NAU3156	21	66.77	Yes
NAU3381	21	75.62	
BNL3147	21	83.25	
HAU0720	21	84.95	
BNL3449	21	86.60	
HAU0423	21	86.60	
BNL3171	21	86.86	Yes
MUSS245	21	90.05	
PGML04105b	21	96.66	
PGML04104	21	97.35	
NAU4014	21	100.57	
NAU2141	21	102.72	Yes

CICR0046	21	103.67	
Gh074a	21	111.31	Yes
DC40250	21	120.05	Yes
HAU0200	21	121.53	
NAU6222	21	122.22	
CGR5602	21	123.37	
CGR5085	21	125.27	
TMB2493	21	128.49	
CM023	21	128.95	Yes
HAU1124	21	130.85	
HAU2026	21	131.53	
PGML00302	21	132.45	
TMB1264	21	133.14	
NAU4855	21	133.64	Yes
PGML01618	21	135.23	
DPL0867	21	137.13	
CGR5800	21	140.92	
PGML01505	21	145.59	
CGR5233	21	146.99	
NAU5217	21	147.91	Yes
TMB2038	21	147.91	
HAU3303	21	148.59	
CGR6799a	21	149.28	
NAU6178	21	149.28	
DPL0500	21	151.69	
TMB1976	21	153.70	Yes
HAU1805	21	158.12	
DC40025	21	164.37	Yes
NAU3354a	21	178.96	Yes
NAU4026	21	194.22	Yes
DPL0212b	21	203.48	
DPL0212a	21	204.40	
TMB0400	21	208.55	
NAU6282	21	209.20	
NAU6224	21	209.65	
CIR068	21	209.88	Yes
HAU3041	21	209.88	
HAU3207	21	209.88	
NAU4004	21	209.88	
HAU3138	21	210.10	
DPL0474	21	210.78	
SWU0304	21	211.47	
PGML03706a	21	213.88	Yes
PGML01089	21	218.55	
CICR0383	21	235.09	Yes
PGML00972	21	238.32	Yes
CGR5866	21	239.96	
Gh132	21	241.43	Yes
NAU0862	21	246.17	
NAU6315	21	250.41	
BNL3279	21	252.43	Yes
NAU6431	21	255.53	
NAU6530	21	257.43	Yes
NAU6697	21	258.11	
NAU6520	21	258.57	
CER0153a	21	262.66	
HAU1660	21	266.75	Yes
DPL0904	21	267.43	
CGR5739	21	268.12	
CGR5400	21	271.67	
HAU1283a	21	278.28	Yes

HAU1809a	21	278.73	Yes
CICR0282	21	278.73	
DC40316	21	279.95	
PGML01580	22	0.00	Yes
PGML03193	22	3.39	
PGML03923	22	11.32	
BNL0448	22	14.20	Yes
TMB1958	22	14.20	
Gh641	22	15.23	
PGML04380	22	18.10	
CGR6410	22	21.23	
NAU2026	22	21.84	Yes
NAU4900	22	22.66	
PGML01941	22	24.13	
NAU3781	22	25.60	
DPL0055	22	25.80	
CICR0482	22	26.00	
Gh022	22	26.00	
Gh200	22	26.20	Yes
Gh591b	22	27.67	
CGR5150	22	32.68	
NAU2302	22	33.93	
NAU4062	22	33.93	
NAU3824b	22	34.13	Yes
NAU5099	22	40.06	Yes
NAU6578	22	49.11	
NAU2376	22	52.74	Yes
NAU6237	22	52.74	
PGML00787	22	53.57	
Gh166a	22	54.39	
PGML01939	22	54.40	
CGR5463	22	54.79	
Gh052	22	54.99	
HAU3012	22	55.81	
DC20081	22	57.06	
NAU3323	22	58.09	
PGML03345	22	58.70	
NAU3942	22	59.52	
NAU2945	22	59.93	
JESPR230	22	62.52	Yes
STV191	22	64.49	
DPL0489	22	66.18	
MUSS066	22	77.76	Yes
PGML02811	22	96.80	Yes
DPL0107b	22	105.37	Yes
PGML04355	22	105.99	
CGR5806	22	106.60	
NAU2783	22	107.63	
CIR224a	22	108.24	Yes
NAU6240	22	109.93	
PGML04045	22	111.18	
PGML00767	22	111.59	
HAU3083	22	113.98	
HAU0747	22	115.01	
PGML04137	22	116.70	
CICR0887	22	118.40	
DPL0515	22	119.43	
DC40071	22	120.04	
CGR5566	22	120.45	
HAU1832	22	121.70	Yes
CGR6791	22	124.96	

NAU2932	22	136.29	Yes
NAU5294	22	141.00	
NAU2977	22	142.70	Yes
HAU1071a	22	143.95	
TMB0206	22	144.59	
NAU3791	22	148.48	
CER0139b	22	149.73	
PGML00036	22	150.55	
CICR0438	22	151.58	
NAU1325	22	152.64	Yes
PGML01868b	23	0.00	Yes
MUSB0442	23	23.95	Yes
PGML04758	23	31.42	Yes
CGR5626	23	38.56	
NAU3888a	23	39.84	Yes
BNL0686a	23	40.92	
DC20058	23	41.09	
HAU2382	23	42.18	Yes
CICR0425	23	56.61	
TMB2354	23	61.72	
TMB2862	23	65.09	Yes
CGR6840	23	65.34	
CGR6122	23	65.61	
CIR060	23	65.61	
DPL0079	23	66.14	
CIR383	23	66.49	
PGML01317	23	66.66	
NAU6279	23	67.37	
NAU6701	23	68.08	
NAU6564	23	68.61	
NAU6508	23	68.96	
JESPR151	23	70.02	Yes
HAU2067	23	71.52	
PGML03706b	23	73.38	
PGML00689	23	78.76	Yes
Gh499	23	86.90	Yes
STV188	23	94.71	Yes
PGML01040	23	111.81	Yes
CICR0237	23	122.69	
PGML00410b	23	125.62	
PGML01044	23	126.70	
TMB2943	23	127.05	
CGR6087	23	127.57	
TMB2901	23	127.92	Yes
NAU3986	23	128.10	
NAU4079	23	128.10	
PGML04407	23	128.10	
PGML02507	23	128.63	
CGR5552	23	131.55	Yes
NAU5508	23	135.39	
CICR0516	23	136.10	
NAU5472	23	136.45	
PGML01868a	23	136.65	Yes
DPL0498	23	137.52	
DPL0378	23	140.23	Yes
PGML03013	23	140.75	
CGR6252b	23	141.46	
TMB0764	23	142.36	
TMB1425	23	142.36	
CGR6576	23	143.63	
CGR5218	23	143.98	

CGR6714	23	144.88	
CICR0508	23	146.94	
SHIN-0154	23	148.02	
CGR6876	23	148.73	
CICR0230	23	149.44	
CGR6943	23	154.82	
BNL3140	23	155.21	Yes
PGML02830	23	164.03	
DC40085	23	168.32	
CGR5494	23	169.65	
NAU0936	23	170.54	Yes
PGML02989	23	170.89	
HAU1085	23	171.60	
HAU0261	23	171.77	
SHIN-0444	23	172.86	
MUCS133	23	175.56	
NAU0859	23	175.78	Yes
CGR5392	23	176.63	
CICR0850	23	176.63	
DPL0044	23	176.63	
MUSS050	23	179.11	Yes
MUSS068	23	183.20	
DPL0262	23	188.04	Yes
NAU3414	23	197.66	
HAU1028	23	203.32	
DPL0627c	23	204.59	
NAU3829	23	205.48	
CGR5862	23	205.66	Yes
NAU5189	23	208.14	Yes
CGR6806	23	208.68	
PGML01613	23	210.15	
DPL0524b	23	212.63	Yes
DPL0514a	23	219.41	Yes
HAU1162	23	220.72	
DPL0031	24	0.00	Yes
HAU1432	24	6.95	Yes
PGML02369	24	7.40	
CGR6147	24	10.42	
PGML04429	24	18.47	
DPL0841b	24	22.75	
NAU2230	24	23.97	Yes
CICR0038	24	24.12	
MUSB0175	24	24.87	
PGML01280	24	26.41	
MUSS021	24	26.57	
BNL3627a	24	26.57	
HAU0755	24	26.57	
PGML04400	24	27.32	
PGML04398	24	27.32	
BNL1646b	24	28.01	Yes
BNL1646a	24	31.57	Yes
NAU3324b	24	38.12	Yes
NAU5031	24	48.39	Yes
CGR5695	24	51.60	Yes
Gh573	24	54.24	
NAU6389	24	55.47	
CGR5503	24	56.21	
DPL0251	24	57.12	
CGR5433	24	57.56	
MUSB0255	24	58.00	Yes
DPL0627b	24	59.91	

PGML02987	24	61.30	
NAU6183	24	61.49	
Gh465	24	61.89	
JESPR127	24	62.03	
CGR5120	24	62.33	
CICR0258	24	62.47	
HAU1905	24	62.47	
CGR5230	24	62.91	
NAU6616	24	63.36	
NAU3988	24	63.65	
NAU2631	24	63.79	
SHIN-1457	24	63.99	
NAU6169	24	64.38	
PGML01755	24	64.82	
NAU6166	24	65.11	
DPL0877	24	65.41	
NAU2619	24	65.55	
NAU5335	24	66.15	
DC40127	24	66.74	
CGR5165	24	67.18	
Gh237	24	67.63	
NAU6235	24	67.63	
PGML02630	24	73.34	Yes
HAU2086b	24	84.08	Yes
Gh171a	24	90.37	Yes
CER0152a	24	93.00	
NAU3207	24	93.15	
NAU2914	24	93.44	Yes
HAU3346b	24	93.58	
PGML01561	24	93.88	
PGML00602	24	94.32	
DPL0133	24	96.05	
DC20017	24	97.02	
TMB0429	24	101.73	
HAU0722	24	104.41	
DPL0534	24	105.35	
PGML03854	24	108.73	Yes
NAU2434	24	113.72	
SHIN-0697	24	115.11	
NAU3804	24	115.70	Yes
NAU3071	24	115.85	
HAU1846	24	116.14	
NAU0858	24	116.14	
MUSS277	24	116.43	
PGML04332	24	118.88	
NAU3721	24	119.63	
HAU3247	24	120.22	
PGML01384	24	120.22	
HAU2015b	24	120.51	
CGR5127	24	120.66	
NAU3562	24	120.95	Yes
MUSS500	24	123.21	
NAU3424	24	128.68	Yes
DPL0214b	24	133.89	
NAU5399	24	134.95	Yes
NAU4099	24	136.51	
NAU3201a	24	136.81	
CGR5813	24	137.40	
NAU1302	24	138.31	Yes
CICR0953a	24	138.90	
HAU0155	24	138.90	

HAU2738	24	138.90	
CICR0628a	24	139.97	
SHIN-0384	24	141.70	
PGML04154a	24	142.92	
DC40229	24	146.49	
NAU2033b	24	148.62	
CGR5447	24	148.77	
HAU1621	24	148.77	
BNL3860	24	152.58	
NAU3158	24	153.52	Yes
PGML01371	24	153.96	
DPL0461	24	154.26	
DPL0068	24	155.01	
NAU2033a	24	155.76	
NAU5282	24	156.70	
PGML04733	24	160.07	
CICR0673	24	160.66	
PGML04093	24	161.89	
NAU3287	24	162.64	Yes
SWU0204	24	162.93	
CGR5423	24	169.19	
DPL0152a	24	169.94	
SHIN-1494b	24	170.53	Yes
DPL0465	25	0.00	Yes
CGR6679	25	2.36	
PGML03690	25	3.63	
BNL0827	25	4.94	Yes
BNL1047	25	13.16	Yes
CICR0507	25	15.45	
CGR5525a	25	17.39	
CGR6584	25	19.10	
DPL0702	25	20.14	
CGR6932a	25	20.14	
PGML02296	25	20.34	Yes
TMB0338	25	21.38	
CGR5115	25	22.00	
DPL0918b	25	22.61	
DPL0441	25	22.81	
CGR5643	25	23.22	
DPL0166a	25	24.05	
CICR0701	25	24.45	
Gh537	25	25.50	Yes
Gh591a	25	26.98	
SHIN-0885	25	27.59	
BNL3806	25	28.44	
CGR5201a	25	29.03	Yes
TMB0313	25	30.74	
SHIN-1131	25	32.96	
SWU0903	25	34.00	Yes
DPL0387	25	34.62	
CER0027	25	36.32	
DC40429	25	36.32	
HAU2759	25	36.52	
BNL1440a	25	37.14	Yes
Gh220	25	37.97	
JESPR215	25	40.33	
Gh224	25	41.45	
CGR5100	25	43.23	Yes
PGML04648	25	49.10	Yes
DPL0124	25	59.41	Yes
HAU1783	25	79.16	Yes

DPL0243b	25	84.26	
CER0086b	25	94.57	Yes
MUSS275	25	104.13	
NAU0905b	25	108.38	Yes
NAU6661	25	109.21	
DPL0075	25	110.92	
Gh515	25	111.85	
NAU6398	25	113.00	
DPL0519	25	113.62	
PGML01304	25	114.66	Yes
PGML03353	25	115.92	
TMB0436	25	115.92	
PGML04301	25	118.82	
HAU1948	25	120.76	
NAU2104	25	120.76	
NAU2378	25	120.76	
NAU2581a	25	120.76	
NAU2581b	25	120.76	
NAU2963	25	120.76	
DPL0290	25	122.46	
DPL0244	25	124.40	
NAU2565	25	125.01	Yes
HAU1931	25	126.38	
CICR0461a	25	127.51	
PGML04555	25	127.73	
NAU3588	25	127.94	
CGR6479	25	128.55	
CGR6799b	25	129.17	
NAU2397	25	132.06	Yes
HAU3258	25	132.06	
NAU3803a	25	132.26	
DPL0705b	25	136.47	Yes
CGR6866	25	142.42	
DPL0257a	25	144.83	
PGML02460	25	145.45	
NAU1204	25	149.37	Yes
DPL0282	25	151.31	
NAU4959	25	159.26	
TMB1448	25	159.36	
COT070	25	159.46	
DC40140	25	160.80	Yes
NAU1100	26	0.00	Yes
HAU1845	26	4.47	
NAU2920	26	4.62	Yes
NAU4925	26	8.20	Yes
DC40080	26	13.15	
CICR0323	26	13.45	
HAU1942	26	13.76	Yes
CGR6772	26	14.87	
DC20086	26	15.82	
NAU4090	26	16.13	Yes
PGML00199	26	17.84	
CGR5733	26	20.51	
NAU0837	26	20.51	
DPL0243a	26	23.46	
DPL0569	26	27.93	
DPL0888	26	29.57	
PGML00811	26	31.38	
NAU2356	26	33.19	Yes
MUSS303	26	33.97	
NAU6161	26	34.44	

DC40116	26	36.99	
HAU0677	26	38.99	
HAU2322	26	39.14	
NAU5425	26	39.29	
BNL0840	26	39.53	
CICR0371	26	39.75	
HAU2730a	26	40.53	
DPL0817	26	42.52	
PGML01594a	26	44.70	
CGR5678	26	45.65	
CGR5527	26	45.80	
CIR039	26	45.86	
DPL0866b	26	45.95	
CGR5991	26	46.41	
PGML02408	26	46.87	
HAU1855	26	46.87	
DPL0796b	26	47.34	
CGR6872	26	47.96	
CGR6728a	26	49.07	
CGR6698b	26	51.83	Yes
DPL0391	26	56.53	
BNL3599b	26	62.45	
JESPR066	26	68.16	Yes
DPL0036b	26	72.67	
CGR5802	26	78.13	
NAU2442	26	82.84	Yes
DPL0190	26	85.20	
PGML03877	26	87.20	
PGML02953a	26	89.56	
SWU1245	26	91.19	
SWU1244	26	92.14	Yes
CICR0669	26	93.26	
HAU1513a	26	95.82	
PGML04053	26	97.45	
PGML01944	26	98.40	
PGML04604a	26	99.86	Yes
Gh171b	26	102.04	Yes
PGML0416	26	105.20	
PGML02879	26	108.56	
HAU1452	26	109.43	
NAU5043	26	110.56	
CICR0876	26	113.00	
NAU5164a	26	114.81	Yes
HAU1434a	26	115.11	
DC40260	26	116.06	
HAU1535	26	116.68	
HAU1292	26	116.99	
CGR6742b	26	117.91	
CGR6702	26	118.09	Yes
HAU1800	26	120.08	
Gh243a	26	125.53	Yes
CGR6880	26	132.07	
CGR6012	26	133.70	
MUSS402	26	138.90	Yes
NAU4912	26	139.52	
MUCS064	26	139.98	
DPL0380b	26	140.77	
NAU2913	26	141.88	
BNL0341	26	143.28	
MUSS439	26	143.65	Yes
HAU0723	26	144.43	

NAU0989	26	144.73	
NAU3293	26	145.68	
BNL3482	26	146.89	
CGR6329	26	148.29	
BNL2495	26	149.57	Yes
CGR6471	26	150.09	
SHIN-0053	26	151.31	
PGML04562	26	156.78	
Gh629	26	161.99	Yes
DPL0915	26	165.80	
NAU4914	26	168.76	
CICR0721	26	169.38	
NAU3905	26	169.38	
NAU5462	26	169.53	
PGML04182	26	170.99	Yes
DPL0890	26	171.61	
CGR5699	26	171.92	
CGR6759	26	172.87	
PGML02118	26	177.36	
NAU2715	26	185.82	Yes
PGML04604b	26	201.83	
BNL3537	26	206.56	Yes
NAU3860	26	208.60	
CICR0266	26	209.06	
DPL0404	26	209.85	
NAU3305	26	210.96	
DPL0226	26	212.42	
HAU1568	26	213.04	
NAU5414	26	213.34	
HAU1830	26	213.97	Yes
COT100	26	216.34	
DPL0917b	26	221.55	Yes
DPL0363	26	226.84	
NAU3084	26	229.82	
MUSS413	26	230.60	
HAU0908	26	231.39	Yes
C2-0055	26	232.33	
CGR5152	26	233.99	Yes

Note:These information from the map reported by Shi et al. 2015
