# Supplementary material for: Examining two sets of introgression lines across multiple environments reveals background-independent and stably expressed quantitative trait loci of fiber quality in cotton
Source: Theor Appl Genet. 2020 Mar 17;133(7):2075–93. doi: 10.1007/s00122-020-03578-0 (PMC7311500; doi:10.1007/s00122-020-03578-0)
Supplement: Supplementary file 5 — Table S2 Correlation analysis of three traits in two populations of two sets of CSSLs with different genetic backgrounds across multiple environments. (PDF 55 kb) [file 122_2020_3578_MOESM5_ESM.pdf]

Table S2 Correlation analysis of three traits in two populations of CSSLs across multi enviro

**Trait**	**Pop**	**Env.**	**09HNA**	**10HNA**	**11HNA**	**11LN**	**11XJS**	**14XJS**	14XJN	
FL	36Pop	10HNA	0.4561***							
FM	36Pop	10HNA	0.3865***							
FS	36Pop	10HNA	0.3668***							
FL	36Pop	11HNA	0.5014***	0.6879***						
FM	36Pop	11HNA	0.3746***	0.7334***						
FS	36Pop	11HNA	0.4543***	0.5885***						
FL	36Pop	11LN	0.5032***	0.6581***	0.7188***					
FM	36Pop	11LN	0.3065***	0.7647***	0.6406***					
FS	36Pop	11LN	0.4449***	0.5913***	0.6115***					
FL	36Pop	11XJS	0.4931***	0.6564***	0.7200***	0.7279***				
FM	36Pop	11XJS	0.3531***	0.6957***	0.6538***	0.6198***				
FS	36Pop	11XJS	0.4408***	0.6196***	0.6658***	0.6513***				
FL	36Pop	14XJS	0.4078***	0.5524***	0.6293***	0.6244***	0.6633***			
FM	36Pop	14XJS	0.2734***	0.6591***	0.6536***	0.5836***	0.6190***			
FS	36Pop	14XJS	0.2712***	0.4383***	0.5163***	0.4887***	0.5337***			
FL	36Pop	14XJN	0.3647***	0.5287***	0.5261***	0.5489***	0.5798***	0.6056***		
FM	36Pop	14XJN	0.2864***	0.6404***	0.6109***	0.5798***	0.5902***	0.7229***		
FS	36Pop	14XJN	0.2871***	0.4296***	0.4139***	0.4382***	0.4856***	0.5508***		
**Trait**	**Pop**	**Env.**	**09HNA**	**10HNA**	**11HNA**	**11XJA**	**14XJA**	**14XJK**	**14HNZ**	**15HNZ**
FL	45Pop	10HNA	0.4943***							
FM	45Pop	10HNA	0.3093***							
FS	45Pop	10HNA	0.2368***							
FL	45Pop	11HNA	0.4473***	0.6156***						
FM	45Pop	11HNA	0.2295***	0.7535***						
FS	45Pop	11HNA	0.4414***	0.4358***						
FL	45Pop	11XJA	0.4645***	0.6893***	0.5474***					
FM	45Pop	11XJA	0.1889***	0.5534***	0.5547***					
FS	45Pop	11XJA	0.5085***	0.3807***	0.6083***					
FL	45Pop	14XJA	0.3768***	0.5121***	0.4404***	0.5625***				
FM	45Pop	14XJA	0.2158***	0.4895***	0.5106***	0.3677***				
FS	45Pop	14XJA	0.4424***	0.2903***	0.4979***	0.5698***				
FL	45Pop	14XJK	0.4285***	0.6190***	0.5790***	0.6722***	0.6357***			
FM	45Pop	14XJK	0.2227***	0.5261***	0.5666***	0.4534***	0.4558***			
FS	45Pop	14XJK	0.3762***	0.2479***	0.4014***	0.4581***	0.5107***			
FL	45Pop	14HNZ	0.3977***	0.6261***	0.5577***	0.5697***	0.4097***	0.5385***		
FM	45Pop	14HNZ	0.3369***	0.6257***	0.7229***	0.5420***	0.4312***	0.5056***		
FS	45Pop	14HNZ	0.3932***	0.3017***	0.5229***	0.5567***	0.4291***	0.3900***		
FL	45Pop	15HNZ	0.3839***	0.5704***	0.5091***	0.5861***	0.3795***	0.5284***	0.6698***	
FM	45Pop	15HNZ	0.2171***	0.5168***	0.5446***	0.3693***	0.3525***	0.3723***	0.6807***	
FS	45Pop	15HNZ	0.3483***	0.2726***	0.4256***	0.4964***	0.3771***	0.3193***	0.5924***	
FL	45Pop	15HNA	0.3078***	0.4936***	0.4605***	0.3983***	0.2699***	0.4046***	0.5110***	0.4031***
FM	45Pop	15HNA	0.2257***	0.4679***	0.5055***	0.4124***	0.3291***	0.3445***	0.5565***	0.4558***
FS	45Pop	15HNA	0.3429***	0.3247***	0.4810***	0.5034***	0.3490***	0.3226***	0.4168***	0.3718***

*, ** and ***, Significant at P ≤ 0.05, 0.01 and 0.001, respectively; Env. Environment
