# Supplementary material for: Examining two sets of introgression lines across multiple environments reveals background-independent and stably expressed quantitative trait loci of fiber quality in cotton
Source: Theor Appl Genet. 2020 Mar 17;133(7):2075–93. doi: 10.1007/s00122-020-03578-0 (PMC7311500; doi:10.1007/s00122-020-03578-0)
Supplement: Supplementary file 6 — Table S3 Details of all QTLs for three traits of fiber quality in CSSL populations. (PDF 468 kb) [file 122_2020_3578_MOESM6_ESM.pdf]

Table S3 Details of all QTLs for three traits of fiber quality in CSSL populations

QTL name	Genetic backgound	Env.	Chr	Position	Nearest marker	LOD	PVE(%)	Add	No. of EE	BI-QTL	previous reports
qFL-C1-1	CCRI36	11LNL	1	10.76	CGR5417	2.68	2.94	-1.61	2		Fang et al., 2014
	CCRI36	11XJS	1	10.76	CGR5417	3.43	3.78	-1.81	2		
qFL-C2-2	CCRI36	14XJN	2	50.01	TMB1578	2.98	3.34	1.98	1		
qFL-C2-4	CCRI36	11HNA	2	137.50	HAU0880	6.82	7.41	0.43	4		Shi et al., 2019
	CCRI36	11LNL	2	137.50	HAU0880	7.03	7.62	0.43	4		
	CCRI36	11XJS	2	137.50	HAU0880	2.53	2.83	0.26	4		
	CCRI36	14XJS	2	137.50	HAU0880	3.21	3.55	0.30	4		
qFL-C2-6	CCRI36	11LNL	2	178.82	NAU2277	5.20	5.68	0.56	1	BI-QTL	
qFL-C3-1	CCRI36	11HNA	3	57.62	NAU1190	3.28	3.62	0.41	2		
	CCRI36	11LNL	3	57.62	NAU1190	3.71	4.09	0.43	2		
qFL-C3-2	CCRI36	11HNA	3	114.40	HAU0195b	7.46	8.03	0.49	4		
	CCRI36	11LNL	3	114.40	HAU0195b	5.67	6.18	0.42	4		
	CCRI36	11XJS	3	114.40	HAU0195b	3.35	3.69	0.32	4		
	CCRI36	14XJS	3	114.40	HAU0195b	3.99	4.38	0.36	4		
qFL-C5-1	CCRI36	11HNA	5	89.92	MUSS317	6.21	6.79	0.59	5		
	CCRI36	11LNL	5	89.92	MUSS317	4.36	4.83	0.49	5		
	CCRI36	11XJS	5	89.92	MUSS317	5.60	6.15	0.54	5		
	CCRI36	14XJN	5	89.92	MUSS317	2.73	3.05	0.44	5		
	CCRI36	14XJS	5	89.92	MUSS317	2.78	3.09	0.41	5		
qFL-C5-2	CCRI36	11HNA	5	141.28	NAU2957	4.35	4.84	0.51	4		Zhai et al., 2016
	CCRI36	11LNL	5	141.28	NAU2957	5.33	5.92	0.55	4		
	CCRI36	11XJS	5	141.28	NAU2957	4.45	4.91	0.50	4		
	CCRI36	14XJN	5	141.28	NAU2957	2.58	2.89	0.44	4		
qFL-C6-3	CCRI36	11HNA	6	54.15	PGML01801	8.18	8.77	0.43	4		
	CCRI36	11LNL	6	54.15	PGML01801	7.11	7.70	0.39	4		
	CCRI36	11XJS	6	54.15	PGML01801	3.38	3.73	0.27	4		
	CCRI36	14XJS	6	54.15	PGML01801	3.34	3.68	0.28	4		
qFL-C7-1	CCRI36	11LNL	7	38.10	HAU1367	3.06	3.36	0.39	1		
qFL-C7-3	CCRI36	10HNA	7	92.24	NAU1085	3.62	3.98	1.13	6	BI-QTL	Sun et al., 2012; Song et al., 2017; Li et al., 2019b; Jamshed et al., 2016; Deng et al., 2019
	CCRI36	11HNA	7	92.24	NAU1085	3.93	4.32	1.01	6		
	CCRI36	11LNL	7	92.24	NAU1085	3.07	3.39	0.87	7		
	CCRI36	11XJS	7	92.24	NAU1085	2.63	2.91	0.80	7		
	CCRI36	14XJN	7	92.24	NAU1085	2.84	3.14	0.97	8		
	CCRI36	14XJS	7	92.24	NAU1085	2.97	3.27	0.89	8		
qFL-C11-1	CCRI36	11HNA	11	252.99	DPL0209	5.52	6.11	0.36	3		
	CCRI36	11LNL	11	252.99	DPL0209	4.82	5.23	0.33	3		
	CCRI36	11XJS	11	252.99	DPL0209	3.80	4.15	0.29	3		
qFL-C14-6	CCRI36	11HNA	14	191.24	NAU3820	3.06	3.38	0.35	3		
	CCRI36	11LNL	14	191.24	NAU3820	4.08	4.48	0.39	3		
	CCRI36	11XJS	14	191.24	NAU3820	3.10	3.41	0.34	3		
qFL-C15-1	CCRI36	10HNA	15	43.61	NAU3177	3.29	3.71	0.33	5	BI-QTL	
	CCRI36	11HNA	15	43.61	NAU3177	4.26	5.36	0.42	5		
	CCRI36	11LNL	15	43.61	NAU3177	4.00	4.89	0.46	5		
	CCRI36	11XJS	15	43.61	NAU3177	4.43	5.18	0.41	5		

	CCRI36	14XJS	15	43.61	NAU3177	6.22	7.11	0.46	5		
qFL-C15-3	CCRI36	14XJN	15	174.48	JESPR152	3.40	3.77	0.64	2		
	CCRI36	14XJS	15	174.48	JESPR152	2.78	3.11	0.64	2		
qFL-C16-1	CCRI36	11XJS	16	29.18	HAU0120	3.16	3.50	0.51	1		
qFL-C16-2	CCRI36	14XJN	16	65.97	BNL2634	3.39	3.86	0.94	1	BI-QTL	Shen et al., 2005; Li et al., 2019b
qFL-C16-3	CCRI36	10HNA	16	176.71	BNL3065	3.10	3.50	-0.63	3		Shi et al., 2019
	CCRI36	11HNA	16	176.71	BNL3065	2.75	3.10	-0.70	3		
	CCRI36	11LNL	16	176.71	BNL3065	3.05	3.44	-0.64	3		
qFL-C17-1	CCRI36	14XJN	17	0.00	CICR0447	3.74	4.19	1.26	2		
	CCRI36	14XJS	17	0.00	CICR0447	3.05	3.52	1.05	2		
qFL-C17-5	CCRI36	11HNA	17	122.79	HAU0195a	3.08	3.42	0.36	4		
	CCRI36	11LNL	17	122.79	HAU0195a	3.84	4.36	0.43	4		
	CCRI36	11XJS	17	122.79	HAU0195a	6.91	7.72	0.56	4		
	CCRI36	14XJS	17	122.79	HAU0195a	6.54	7.31	0.53	4		
qFL-C19-5	CCRI36	11HNA	19	196.95	NAU2274	3.67	3.87	0.31	4		Shao et al., 2014; Wang et al., 2017a
	CCRI36	11LNL	19	196.95	NAU2274	6.40	6.77	0.40	4		
	CCRI36	11XJS	19	196.95	NAU2274	4.47	4.86	0.33	4		
	CCRI36	14XJS	19	196.95	NAU2274	5.92	6.28	0.39	4		
qFL-C20-1	CCRI36	11HNA	20	12.33	CGR5565a	7.66	8.25	0.44	4		Zhai et al., 2016; Shi et al., 2019; Deng et al., 2019
	CCRI36	11LNL	20	12.33	CGR5565a	3.31	3.65	0.30	4		
	CCRI36	11XJS	20	12.33	CGR5565a	4.94	5.39	0.35	4		
	CCRI36	14XJS	20	12.33	CGR5565a	7.85	8.43	0.45	4		
qFL-C20-4	CCRI36	10HNA	20	175.52	NAU3665	6.57	7.16	0.36	5		Zhang et al., 2012; Song et al., 2017
	CCRI36	11HNA	20	175.52	NAU3665	6.18	6.97	0.38	5		
	CCRI36	11LNL	20	175.52	NAU3665	3.48	3.97	0.33	5		
	CCRI36	11XJS	20	175.52	NAU3665	10.58	11.49	0.47	5		
	CCRI36	14XJS	20	175.52	NAU3665	9.07	10.15	0.45	5		
qFL-C21-3	CCRI36	14XJN	21	213.88	PGML03706a	2.79	3.12	0.73	1		Zhang et al., 2016
qFL-C22-1	CCRI36	11HNA	22	21.84	NAU2026	3.51	3.91	0.33	2		Song et al., 2017
	CCRI36	11LNL	22	21.84	NAU2026	3.60	4.03	0.34	2		
qFL-C23-2	CCRI36	11LNL	23	31.42	PGML04758	3.62	3.98	0.48	1		
qFL-C23-3	CCRI36	11HNA	23	208.14	NAU5189	4.87	5.41	0.35	4		
	CCRI36	11LNL	23	208.14	NAU5189	3.05	3.40	0.29	4		
	CCRI36	11XJS	23	208.14	NAU5189	7.37	8.10	0.43	4		
	CCRI36	14XJS	23	208.14	NAU5189	10.05	10.83	0.51	4		
qFL-C24-1	CCRI36	10HNA	24	153.52	NAU3158	3.40	3.71	-0.97	1		
qFL-C26-1	CCRI36	14XJN	26	82.84	NAU2442	3.27	3.63	0.93	1		
qFM-C1-1	CCRI36	10HNA	1	4.84	HAU1619	2.61	2.91	0.12	3		Shao et al., 2014
	CCRI36	11LNL	1	4.84	HAU1619	3.91	4.31	0.11	3		
	CCRI36	11XJS	1	4.84	HAU1619	2.88	3.19	0.11	3		
qFM-C1-2	CCRI36	11XJS	1	27.95	NAU4074	2.85	3.15	-0.16	1		Shi et al., 2019
qFM-C3-1	CCRI36	14XJS	3	105.29	HAU1022	7.12	6.99	-0.15	6		zhai et al., 2016
	CCRI36	11HNA	3	105.29	HAU1022	9.82	9.94	-0.22	6		
	CCRI36	11LNL	3	105.29	HAU1022	8.79	9.98	-0.22	6		
	CCRI36	11XJS	3	105.29	HAU1022	9.81	10.82	-0.19	6		
	CCRI36	10HNA	3	105.29	HAU1022	10.17	10.82	-0.22	6		
	CCRI36	14XJN	3	105.29	HAU1022	10.21	11.90	-0.18	6		
qFM-C3-2	CCRI36	11HNA	3	114.40	HAU0195b	2.76	3.05	-0.08	1		Ma et al., 2017
qFM-C4-2	CCRI36	11LNL	4	73.91	Gh117	2.73	3.03	0.15	1		
qFM-C7-3	CCRI36	11LNL	7	92.24	NAU1085	3.41	3.76	-0.25	2		Sun et al., 2012
	CCRI36	11LNL	7	92.24	NAU1085	3.41	3.76	-0.25	2		
qFM-C12-1	CCRI36	10HNA	12	114.96	HAU1454	5.06	5.52	-0.10	2		Yang et al., 2015
	CCRI36	11LNL	12	114.96	HAU1454	4.17	4.58	-0.07	2		
qFM-C14-1	CCRI36	10HNA	14	104.33	BNL3145	6.63	7.18	-0.21	6		said et al., 2015b
	CCRI36	11HNA	14	104.33	BNL3145	6.71	7.25	-0.24	6		

	CCRI36	11LNL	14	104.33	BNL3145	13.33	13.91	-0.26	6		
	CCRI36	11XJS	14	104.33	BNL3145	4.38	4.80	-0.15	6		
	CCRI36	14XJN	14	104.33	BNL3145	7.64	8.22	-0.24	6		
	CCRI36	14XJS	14	104.33	BNL3145	7.52	8.09	-0.26	6		
qFM-C16-1	CCRI36	10HNA	16	79.08	NAU5408	2.52	2.77	-0.14	2		Li et al., 2019b
	CCRI36	11XJS	16	79.08	NAU5408	3.19	3.47	-0.18	2		
qFM-C17-1	CCRI36	11LNL	17	0.00	CICR0447	3.43	3.76	0.12	1		
qFM-C17-2	CCRI36	10HNA	17	47.26	NAU2909	8.97	9.59	-0.18	6	BI-QTL	Zhai et al., 2016
	CCRI36	11HNA	17	47.26	NAU2909	6.50	7.04	-0.17	6		
	CCRI36	11LNL	17	47.26	NAU2909	9.05	9.67	-0.20	6		
	CCRI36	11XJS	17	47.26	NAU2909	6.46	6.99	-0.18	6		
	CCRI36	14XJN	17	47.26	NAU2909	8.94	9.55	-0.16	6		
	CCRI36	14XJS	17	47.26	NAU2909	4.52	4.95	-0.12	6		
qFM-C17-3	CCRI36	10HNA	17	93.28	HAU0764	10.51	11.14	-0.20	6		
	CCRI36	11HNA	17	93.28	HAU0764	10.92	11.54	-0.23	6		
	CCRI36	11LNL	17	93.28	HAU0764	7.26	7.83	-0.15	6		
	CCRI36	11XJS	17	93.28	HAU0764	11.54	12.15	-0.23	6		
	CCRI36	14XJN	17	93.28	HAU0764	10.80	11.42	-0.24	6		
	CCRI36	14XJS	17	93.28	HAU0764	11.12	11.74	-0.19	6		
qFM-C17-4	CCRI36	10HNA	17	122.79	HAU0195a	5.48	6.10	-0.22	6		Wang et al., 2016
	CCRI36	11HNA	17	122.79	HAU0195a	3.80	4.32	-0.21	6		
	CCRI36	11LNL	17	122.79	HAU0195a	4.82	5.27	-0.24	6		
	CCRI36	11XJS	17	122.79	HAU0195a	5.50	6.11	-0.24	6		
	CCRI36	14XJN	17	122.79	HAU0195a	7.30	8.08	-0.23	6		
	CCRI36	14XJS	17	122.79	HAU0195a	3.57	4.11	-0.16	6		
qFM-C19-1	CCRI36	10HNA	19	0.00	CICR0851	2.58	2.86	-0.41	2		
	CCRI36	14XJN	19	0.00	CICR0851	2.56	2.83	-0.39	2		
qFM-C19-[illegible]	CCRI36	14XJN	19	145.90	NAU5475	2.70	3.02	-0.16	1		
qFM-C19-[illegible]	CCRI36	10HNA	19	237.78	Gh447	3.60	4.00	-0.23	5		
	CCRI36	11HNA	19	237.78	Gh447	2.63	2.93	-0.19	5		
	CCRI36	11XJS	19	237.78	Gh447	4.65	5.12	-0.20	5		
	CCRI36	14XJN	19	237.78	Gh447	3.33	3.69	-0.20	5		
	CCRI36	14XJS	19	237.78	Gh447	3.86	4.28	-0.20	5		
qFM-C20-1	CCRI36	14XJS	20	200.39	DPL0442	2.99	3.30	0.22	1		Fang et al., 2014
qFM-C23-1	CCRI36	11HNA	23	70.02	JESPR151	3.46	3.84	-0.51	1		
qFM-C25-4	CCRI36	14XJN	25	43.23	CGR5100	3.73	4.28	-0.24	1		
qFM-C25-5	CCRI36	10HNA	25	79.16	HAU1783	3.08	3.36	-0.26	2		Li et al., 2019b
	CCRI36	11HNA	25	79.16	HAU1783	3.35	3.52	-0.20	2		
qFM-C25-6	CCRI36	10HNA	25	94.57	CER0086b	4.16	4.60	-0.15	5		
	CCRI36	11HNA	25	94.57	CER0086b	3.30	3.67	-0.17	5		
	CCRI36	11XJS	25	94.57	CER0086b	2.71	2.99	-0.13	5		
	CCRI36	14XJN	25	94.57	CER0086b	2.69	2.99	-0.15	5		
	CCRI36	14XJS	25	94.57	CER0086b	3.43	3.82	-0.18	5		
qFS-C2-2	CCRI36	14XJS	2	154.42	HAU1475	2.56	2.83	0.62	1		
qFS-C3-1	CCRI36	14XJN	3	93.70	MUSB0087	2.59	2.87	0.82	1		
qFS-C3-2	CCRI36	11HNA	3	114.40	HAU0195b	4.29	4.70	0.46	1		
qFS-C5-1	CCRI36	11HNA	5	89.92	MUSS317	2.63	2.93	0.48	1		
qFS-C7-4	CCRI36	10HNA	7	92.24	NAU1085	3.27	3.60	1.29	4	BI-QTL	Sun et al., 2012; Song et al., 2017; Li et al., 2019b; Jamshed et al., 2016
	CCRI36	11HNA	7	92.24	NAU1085	3.16	3.49	1.13	4		
	CCRI36	11LNL	7	92.24	NAU1085	2.95	3.26	0.96	4		
	CCRI36	11XJS	7	92.24	NAU1085	2.88	3.19	0.95	4		
qFS-C11-1	CCRI36	11LNL	11	86.66	CGR6697b	2.80	3.10	0.39	1		Wang et al., 2017b; Zhai et al., 2016; Shi et al., 2019
qFS-C11-2	CCRI36	11LNL	11	90.27	HAU0848	2.94	3.32	0.61	1		
qFS-C11-4	CCRI36	09HNA	11	126.68	NAU3234	10.19	10.81	-7.76	1		

qFS-C11-7[illegible]	CCRI36	11HNA	11	252.99	DPL0209	3.90	4.36	0.38	1		
qFS-C13-3	CCRI36	11LNL	13	184.33	NAU3468	2.61	2.89	0.64	1		Shi et al., 2019
qFS-C15-3	CCRI36	14XJS	15	43.61	NAU3177	2.51	3.17	0.44	1	BI-QTL	Li et al., 2019b
qFS-C15-5	CCRI36	14XJS	15	174.48	JESPR152	2.54	2.83	0.77	1		Guo et al., 2018
qFS-C16-2	CCRI36	11HNA	16	29.18	HAU0120	2.92	3.24	0.63	2		
	CCRI36	11XJS	16	29.18	HAU0120	4.00	4.42	0.65	2		
qFS-C16-3	CCRI36	11HNA	16	65.97	BNL2634	4.05	4.67	1.14	2	BI-QTL	Shen et al., 2005; Li et al., 2019b
	CCRI36	11LNL	16	65.97	BNL2634	4.03	4.61	1.00	2		
qFS-C16-4	CCRI36	10HNA	16	176.71	BNL3065	3.81	4.21	-0.99	4		Shi et al., 2019
	CCRI36	11HNA	16	176.71	BNL3065	3.02	3.39	-0.69	4		
	CCRI36	11LNL	16	176.71	BNL3065	3.32	3.73	-0.82	4		
	CCRI36	11XJS	16	176.71	BNL3065	3.28	3.69	-0.72	4		
qFS-C17-3	CCRI36	11HNA	17	122.79	HAU0195a	2.68	3.03	0.44	2	BI-QTL	Li et al., 2019b
	CCRI36	11LNL	17	122.79	HAU0195a	2.57	2.91	0.38	2		
qFS-C20-1	CCRI36	11HNA	20	12.33	CGR5565a	3.78	4.16	0.40	1		Zhai et al., 2016; Shi et al., 2019; Deng et al., 2019
qFS-C20-2	CCRI36	11HNA	20	96.25	Gh119	2.51	2.81	0.72	1		Song et al., 2017
qFS-C20-4	CCRI36	11HNA	20	152.59	NAU3813b	2.74	3.05	0.59	1		Song et al., 2017
qFS-C20-5	CCRI36	10HNA	20	162.96	TMB1125	3.44	3.79	0.67	2		Song et al., 2017
	CCRI36	11LNL	20	162.96	TMB1125	3.35	3.70	0.52	2		
qFS-C20-6	CCRI36	11HNA	20	175.52	NAU3665	3.66	4.06	0.35	1		
qFS-C21-3	CCRI36	14XJS	21	213.88	PGML03706a	2.68	3.00	0.89	1		
qFS-C23-2	CCRI36	11HNA	23	208.14	NAU5189	3.17	3.57	0.36	1		
qFS-C25-3	CCRI36	14XJS	25	37.14	BNL1440a	2.54	2.81	0.87	1		Sun et al., 2012; Jamshed et al., 2016
qFS-C26-3	CCRI36	14XJS	26	82.84	NAU2442	2.63	2.91	1.04	1		
qFS-C26-4	CCRI36	09HNA	26	233.99	CGR5152	7.13	7.70	-5.94	1		
qFL-C1-2	CCRI45	14HNZ	1	154.73	NAU2095	2.74	3.71	-0.53	2		
	CCRI45	15HNA	1	154.73	NAU2095	2.53	3.43	-0.66	2		
qFL-C2-1	CCRI45	10HNA	2	15.12	BNL1434	11.98	15.51	1.37	8		Fang et al., 2014; Said et al., 2015b
	CCRI45	11XJA	2	15.12	BNL1434	12.49	16.31	1.38	8		
	CCRI45	11HNA	2	15.12	BNL1434	5.92	8.11	1.03	8		
	CCRI45	14XJA	2	15.12	BNL1434	3.44	4.80	0.61	8		
	CCRI45	14XJK	2	15.12	BNL1434	3.28	4.58	0.76	8		
	CCRI45	14HNZ	2	15.12	BNL1434	7.54	10.05	0.93	8		
	CCRI45	15HNA	2	15.12	BNL1434	4.70	6.39	0.96	8		
	CCRI45	15HNZ	2	15.12	BNL1434	7.63	9.91	0.91	8		
qFL-C2-3	CCRI45	10HNA	2	54.78	HAU1980b	10.65	13.68	1.34	8		Zhai et al., 2016
	CCRI45	11XJA	2	54.78	HAU1980b	11.96	15.33	1.40	8		
	CCRI45	11HNA	2	54.78	HAU1980b	7.11	9.67	1.17	8		
	CCRI45	14XJA	2	54.78	HAU1980b	4.50	6.21	0.72	8		
	CCRI45	14XJK	2	54.78	HAU1980b	3.43	4.80	0.81	8		
	CCRI45	14HNZ	2	54.78	HAU1980b	8.60	11.40	1.03	8		
	CCRI45	15HNA	2	54.78	HAU1980b	6.42	8.66	1.16	8		
	CCRI45	15HNZ	2	54.78	HAU1980b	7.53	9.81	0.95	8		
qFL-C2-5	CCRI45	09HNA	2	159.08	TMB1580	2.85	3.87	0.62	6		
	CCRI45	10HNA	2	159.08	TMB1580	4.43	5.94	0.69	6		
	CCRI45	11XJA	2	159.08	TMB1580	4.30	5.79	0.67	6		
	CCRI45	11HNA	2	159.08	TMB1580	9.85	12.73	1.05	6		
	CCRI45	14XJK	2	159.08	TMB1580	3.28	4.44	0.61	6		

	CCRI45	15HNA	2	159.08	TMB1580	3.46	4.66	0.66	6		
qFL-C2-6	CCRI45	10HNA	2	178.82	NAU2277	4.42	5.92	0.71	4	BI-QTL	
	CCRI45	11XJA	2	178.82	NAU2277	4.56	6.13	0.71	4		
	CCRI45	11HNA	2	178.82	NAU2277	2.56	3.48	0.56	4		
	CCRI45	14HNZ	2	178.82	NAU2277	3.19	4.32	0.51	4		
qFL-C5-3	CCRI45	11HNA	5	172.74	NAU2296b	2.91	4.32	-1.24	1		
qFL-C6-1	CCRI45	10HNA	6	29.82	Gh082	3.59	3.91	0.45	1		
qFL-C6-2	CCRI45	14XJK	6	31.00	BNL1440b	5.78	7.68	-0.92	1		
qFL-C7-2	CCRI45	11XJA	7	53.19	PGML01958	4.27	5.75	0.62	3		
	CCRI45	11HNA	7	53.19	PGML01958	6.81	8.99	0.82	3		
	CCRI45	14XJK	7	53.19	PGML01958	2.98	4.03	0.54	3		
qFL-C7-3	CCRI45	10HNA	7	92.24	NAU1085	10.65	13.64	1.34	5	BI-QTL	Sun et al., 2012; Song et al., 2017; Li et al., 2019b; Jamshed et al., 2016; Deng et al., 2019
	CCRI45	11HNA	7	92.24	NAU1085	7.11	9.64	1.17	5		
	CCRI45	14XJA	7	92.24	NAU1085	4.50	6.19	0.72	5		
	CCRI45	14XJK	7	92.24	NAU1085	3.43	4.78	0.81	5		
	CCRI45	14HNZ	7	92.24	NAU1085	8.60	11.38	1.03	5		
qFL-C10-1	CCRI45	11XJA	10	91.66	DPL0468	2.70	3.12	0.40	1		Li et al., 2019b
qFL-C10-2	CCRI45	11XJA	10	178.11	BNL3563	5.48	7.32	0.75	3		Shi et al., 2019
	CCRI45	11HNA	10	178.11	BNL3563	7.96	10.42	0.95	3		
	CCRI45	14XJK	10	178.11	BNL3563	3.02	4.09	0.58	3		
qFL-C12-1	CCRI45	10HNA	12	16.72	NAU3862	4.67	6.25	0.77	4		
	CCRI45	11XJA	12	16.72	NAU3862	5.92	7.88	0.85	4		
	CCRI45	14HNZ	12	16.72	NAU3862	2.68	3.63	0.49	4		
	CCRI45	15HNZ	12	16.72	NAU3862	3.87	5.19	0.58	4		
qFL-C12-2	CCRI45	11XJA	12	72.93	NAU5139	3.78	3.59	0.42	1		
qFL-C12-3	CCRI45	11XJA	12	84.57	HAU1361	3.23	4.40	0.57	2		
	CCRI45	15HNZ	12	84.57	HAU1361	3.17	4.28	0.31	2		
qFL-C12-4	CCRI45	09HNA	12	110.86	NAU4889	12.12	15.42	1.29	7		
	CCRI45	10HNA	12	110.86	NAU4889	3.57	4.81	0.71	7		
	CCRI45	11XJA	12	110.86	NAU4889	5.99	7.95	0.89	7		
	CCRI45	11HNA	12	110.86	NAU4889	3.41	4.61	0.57	7		
	CCRI45	14XJK	12	110.86	NAU4889	3.17	4.28	0.55	7		
	CCRI45	14HNZ	12	110.86	NAU4889	5.54	7.40	0.85	7		
	CCRI45	15HNZ	12	110.86	NAU4889	3.61	4.87	0.78	7		
qFL-C12-5	CCRI45	11XJA	12	114.96	HAU1454	2.74	4.17	0.29	1		
qFL-C13-1	CCRI45	10HNA	13	8.68	BNL1707	3.07	4.17	0.60	2		
	CCRI45	11XJA	13	8.68	BNL1707	2.76	3.75	0.58	2		
qFL-C13-2	CCRI45	10HNA	13	40.66	NAU3948	2.54	3.44	0.52	4		
	CCRI45	11XJA	13	40.66	NAU3948	3.11	4.21	0.70	4		
	CCRI45	11HNA	13	40.66	NAU3948	2.93	3.98	0.66	4		
	CCRI45	15HNZ	13	40.66	NAU3948	6.05	8.03	1.00	4		
qFL-C13-3	CCRI45	10HNA	13	164.25	CGR5242	2.74	3.71	0.61	4		
	CCRI45	11XJA	13	164.25	CGR5242	3.26	4.40	0.68	4		
	CCRI45	11HNA	13	164.25	CGR5242	5.36	7.14	0.88	4		
	CCRI45	14XJK	13	164.25	CGR5242	4.37	5.88	0.75	4		
qFL-C14-1	CCRI45	11XJA	14	40.73	NAU3648	7.92	10.41	1.04	2		Zhai et al., 2016
	CCRI45	14XJA	14	40.73	NAU3648	2.81	3.81	0.51	2		
qFL-C14-2	CCRI45	14XJK	14	46.54	NAU5421	2.68	3.64	-0.83	1		Zhai et al., 2016
qFL-C14-3	CCRI45	11HNA	14	83.01	BNL1059	3.83	5.16	-0.91	1		He et al., 2007; Lin et al., 2005
qFL-C14-4	CCRI45	10HNA	14	171.34	NAU3225	3.99	5.37	0.45	3		
	CCRI45	11XJA	14	171.34	NAU3225	6.80	8.98	0.73	3		
	CCRI45	14XJA	14	171.34	NAU3225	5.44	7.26	0.65	3		
qFL-C14-5	CCRI45	10HNA	14	181.56	NAU3214	5.48	7.30	0.67	2		Said et al., 2015b
	CCRI45	11XJA	14	181.56	NAU3214	3.41	4.62	0.53	2		
qFL-C14-7	CCRI45	11HNA	14	206.88	HAU1219a	3.48	4.70	-0.79	2		

	CCRI45	15HNZ	14	206.88	HAU1219a	2.92	3.95	-0.58	2		
qFL-C15-1	CCRI45	09HNA	15	43.61	NAU3177	7.98	10.00	1.75	8	BI-QTL	
	CCRI45	10HNA	15	43.61	NAU3177	2.98	4.08	1.10	8		
	CCRI45	11XJA	15	43.61	NAU3177	4.09	5.44	1.05	8		
	CCRI45	11HNA	15	43.61	NAU3177	2.93	4.04	1.20	8		
	CCRI45	14XJK	15	43.61	NAU3177	3.52	4.81	1.27	8		
	CCRI45	14HNZ	15	43.61	NAU3177	4.63	6.15	1.32	8		
	CCRI45	15HNA	15	43.61	NAU3177	3.17	4.15	0.90	8		
	CCRI45	15HNZ	15	43.61	NAU3177	6.39	8.41	1.52	8		
qFL-C15-2	CCRI45	10HNA	15	66.78	HAU0670	3.25	4.38	0.33	4		
	CCRI45	11XJA	15	66.78	HAU0670	5.41	7.21	0.52	4		
	CCRI45	14XJA	15	66.78	HAU0670	3.90	5.27	0.43	4		
	CCRI45	15HNZ	15	66.78	HAU0670	2.61	3.54	0.29	4		
qFL-C15-4	CCRI45	11HNA	15	177.43	MUSS085	5.01	6.70	-0.78	1		Li et al., 2019b
qFL-C16-2	CCRI45	09HNA	16	65.97	BNL2634	13.43	16.95	1.24	8	BI-QTL	Shen et al., 2005; Li et al., 2019b
	CCRI45	10HNA	16	65.97	BNL2634	4.96	6.63	0.76	8		
	CCRI45	11XJA	16	65.97	BNL2634	4.25	5.71	0.78	8		
	CCRI45	11HNA	16	65.97	BNL2634	4.36	5.86	0.59	8		
	CCRI45	14XJK	16	65.97	BNL2634	4.47	5.98	0.77	8		
	CCRI45	14HNZ	16	65.97	BNL2634	6.33	8.38	0.84	8		
	CCRI45	15HNA	16	65.97	BNL2634	6.43	8.54	0.83	8		
	CCRI45	15HNZ	16	65.97	BNL2634	3.93	5.27	0.56	8		
qFL-C17-2	CCRI45	11XJA	17	23.34	HAU2014	4.76	6.39	-0.76	1		
qFL-C17-3	CCRI45	09HNA	17	47.26	NAU2909	10.78	13.85	1.18	7		
	CCRI45	10HNA	17	47.26	NAU2909	2.78	3.77	0.61	7		
	CCRI45	11XJA	17	47.26	NAU2909	2.56	3.48	0.64	7		
	CCRI45	11HNA	17	47.26	NAU2909	5.22	6.96	0.81	7		
	CCRI45	14XJK	17	47.26	NAU2909	2.65	3.60	0.49	7		
	CCRI45	14HNZ	17	47.26	NAU2909	5.21	6.93	0.88	7		
	CCRI45	15HNA	17	47.26	NAU2909	4.63	6.22	0.75	7		
qFL-C17-4	CCRI45	11HNA	17	56.36	NAU2325	4.36	5.85	-0.77	1		Shen et al., 2005
qFL-C19-1	CCRI45	11XJA	19	17.39	NAU3405	4.28	5.76	0.70	3		Wang et al., 2017a
	CCRI45	11HNA	19	17.39	NAU3405	7.37	9.70	0.96	3		
	CCRI45	14XJK	19	17.39	NAU3405	2.51	3.41	0.56	3		
qFL-C19-2	CCRI45	10HNA	19	88.23	DPL0444	3.89	5.23	0.66	4		Wang et al., 2017a
	CCRI45	11XJA	19	88.23	DPL0444	3.04	4.11	0.64	4		
	CCRI45	11HNA	19	88.23	DPL0444	2.63	3.59	0.54	4		
	CCRI45	15HNA	19	88.23	DPL0444	6.07	8.05	0.85	4		
qFL-C19-3	CCRI45	14XJA	19	99.97	BNL1064	2.83	3.86	0.84	1		
qFL-C19-4	CCRI45	11HNA	19	161.66	DPL0215	3.87	5.21	-0.83	1		
qFL-C20-2	CCRI45	09HNA	20	113.72	HAU1491b	4.13	5.55	0.65	3		
	CCRI45	11XJA	20	113.72	HAU1491b	3.77	5.08	0.57	3		
	CCRI45	11HNA	20	113.72	HAU1491b	3.65	4.94	0.53	3		
qFL-C20-3	CCRI45	11XJA	20	139.90	HAU1491a	7.79	10.25	0.59	4		
	CCRI45	14XJA	20	139.90	HAU1491a	3.66	4.93	0.33	4		
	CCRI45	14XJK	20	139.90	HAU1491a	3.78	5.07	0.35	4		
	CCRI45	15HNZ	20	139.90	HAU1491a	3.94	5.30	0.44	4		
qFL-C21-1	CCRI45	11HNA	21	57.89	CGR5217	2.77	3.76	-0.68	2		
	CCRI45	14XJA	21	57.89	CGR5217	2.97	4.02	-0.54	2		
qFL-C21-2	CCRI45	10HNA	21	62.63	NAU3731	2.75	3.73	-0.65	1		
qFL-C21-4	CCRI45	10HNA	21	238.32	PGML00972	3.31	4.31	1.80	5		
	CCRI45	11XJA	21	238.32	PGML00972	2.65	3.60	0.56	5		
	CCRI45	11HNA	21	238.32	PGML00972	7.61	9.99	1.15	5		
	CCRI45	14HNZ	21	238.32	PGML00972	3.21	4.34	0.73	5		
	CCRI45	15HNZ	21	238.32	PGML00972	2.95	4.01	0.69	5		
qFL-C21-5	CCRI45	10HNA	21	241.43	Gh132	3.32	4.53	1.43	8		
	CCRI45	11XJA	21	241.43	Gh132	2.70	3.66	0.62	8		

	CCRI45	11HNA	21	241.43	Gh132	2.50	3.40	0.49	8		
	CCRI45	14XJK	21	241.43	Gh132	3.42	4.59	1.46	8		
	CCRI45	14HNZ	21	241.43	Gh132	4.31	5.77	1.38	8		
	CCRI45	14HNZ	21	241.43	Gh132	5.64	7.20	1.90	8		
	CCRI45	15HNA	21	241.43	Gh132	3.45	4.64	0.74	8		
	CCRI45	15HNZ	21	241.43	Gh132	3.47	4.67	0.57	8		
qFL-C22-2	CCRI45	10HNA	22	142.70	NAU2977	3.50	4.73	0.53	4		
	CCRI45	11XJA	22	142.70	NAU2977	4.08	5.49	0.68	4		
	CCRI45	11HNA	22	142.70	NAU2977	4.16	5.60	0.67	4		
	CCRI45	14HNZ	22	142.70	NAU2977	2.71	3.69	0.58	4		
qFL-C22-3	CCRI45	11XJA	22	152.64	NAU1325	7.55	9.91	0.92	3		
	CCRI45	11HNA	22	152.64	NAU1325	2.58	3.51	0.54	3		
	CCRI45	14XJK	22	152.64	NAU1325	4.44	5.98	0.68	3		
qFL-C23-1	CCRI45	11XJA	23	23.95	MUSB0442	3.84	5.18	0.45	1		
qFL-C25-1	CCRI45	14XJK	25	29.03	CGR5201a	3.94	5.30	-0.70	1		Sun et al., 2012; Li et al., 2019b; Jamshed et al., 2016
qFL-C26-2	CCRI45	14XJK	26	143.65	MUSS439	4.23	5.68	-0.93	1		
qFL-C26-3	CCRI45	14XJK	26	149.57	BNL2495	9.31	12.36	-2.47	1		Said et al., 2015b
qFM-C2-1	CCRI45	14HNZ	2	118.16	CGR5201b	5.04	6.67	-0.45	1		
qFM-C4-1	CCRI45	14HNZ	4	28.82	NAU0786	4.32	6.00	-0.84	1		
qFM-C5-1	CCRI45	14HNZ	5	89.92	MUSS317	2.58	3.51	-0.08	1		
qFM-C5-2	CCRI45	11HNA	5	123.29	DPL0384	2.76	3.82	0.32	1		Tang et al., 2015
qFM-C5-3	CCRI45	10HNA	5	158.09	NAU3824a	5.76	7.66	0.27	3		Wang et al., 2017b
	CCRI45	11HNA	5	158.09	NAU3824a	3.02	4.09	0.17	3		
	CCRI45	15HNA	5	158.09	NAU3824a	2.54	3.45	0.16	3		
qFM-C5-4	CCRI45	10HNA	5	205.12	NAU3402	4.01	5.40	0.24	1		
qFM-C7-1	CCRI45	14XJK	7	38.10	HAU1367	2.89	5.63	-0.10	1		Shao et al., 2014
qFM-C7-2	CCRI45	09HNA	7	85.06	NAU2002	3.14	7.37	0.87	2		Li et al., 2019b
	CCRI45	10HNA	7	85.06	NAU2002	2.87	6.25	0.58	2		
qFM-C11-1	CCRI45	10HNA	11	21.99	HAU2974	2.55	3.61	0.26	1		
qFM-C13-1	CCRI45	11HNA	13	0.00	HAU0371	3.34	4.51	0.28	1		
qFM-C15-1	CCRI45	10HNA	15	26.90	HAU1058a	2.91	3.99	0.19	2		Chen et al., 2018; Wang et al., 2016
	CCRI45	11HNA	15	26.90	HAU1058a	2.62	3.59	0.15	2		
qFM-C16-1	CCRI45	09HNA	16	16.79	CGR5149	2.52	3.43	0.24	2		
	CCRI45	10HNA	16	16.79	CGR5149	3.34	4.52	0.20	2		
qFM-C17-2	CCRI45	15HNA	17	47.26	NAU2909	2.72	3.68	-0.18	2	BI-QTL	Zhai et al., 2016
	CCRI45	15HNZ	17	47.26	NAU2909	4.30	5.76	-0.20	2		
qFM-C21-1	CCRI45	14XJA	21	235.09	CICR0383	2.76	3.75	0.23	1		
qFM-C24-1	CCRI45	11HNA	24	115.70	NAU3804	3.25	4.39	0.22	1		
qFM-C24-2	CCRI45	09HNA	24	134.95	NAU5399	2.54	3.49	0.69	3		Shi et al., 2019
	CCRI45	10HNA	24	134.95	NAU5399	3.94	5.36	0.99	3		
	CCRI45	11HNA	24	134.95	NAU5399	3.88	5.26	1.36	3		
qFM-C25-1	CCRI45	10HNA	25	4.94	BNL0827	3.45	4.69	0.10	2		Chen et al., 2018; Wang et al., 2011
	CCRI45	11HNA	25	4.94	BNL0827	2.78	3.79	0.11	2		
qFM-C25-2	CCRI45	14HNZ	25	25.50	Gh537	6.27	8.31	-0.27	2		Li et al., 2019b; Jamshed et al., 2016
	CCRI45	15HNZ	25	25.50	Gh537	3.27	4.40	-0.18	2		
qFM-C25-3	CCRI45	14HNZ	25	29.03	CGR5201a	3.11	4.20	-0.17	2		Sun et al., 2012; Shen et al., 2005; Li et al., 2019b
	CCRI45	15HNZ	25	29.03	CGR5201a	6.23	8.37	-0.45	2		
qFS-C2-1	CCRI45	09HNA	2	54.78	HAU1980b	2.66	3.61	0.85	7		
	CCRI45	11XJA	2	54.78	HAU1980b	7.59	9.88	1.27	7		

	CCRI45	11HNA	2	54.78	HAU1980b	6.13	8.37	1.23	7		
	CCRI45	14XJA	2	54.78	HAU1980b	3.72	5.18	0.84	7		
	CCRI45	14HNZ	2	54.78	HAU1980b	4.83	6.61	1.10	7		
	CCRI45	15HNA	2	54.78	HAU1980b	6.35	8.50	1.83	7		
	CCRI45	15HNZ	2	54.78	HAU1980b	2.56	3.47	0.68	7		
qFS-C2-2	CCRI45	11XJA	2	178.82	NAU2277	3.97	5.36	0.75	3		
	CCRI45	11HNA	2	178.82	NAU2277	4.57	6.12	0.84	3		
	CCRI45	15HNA	2	178.82	NAU2277	2.66	3.60	0.96	3		
qFS-C6-1	CCRI45	11XJA	6	0.00	NAU5038	3.51	4.76	0.58	4		
	CCRI45	14XJA	6	0.00	NAU5038	3.11	4.21	0.50	4		
	CCRI45	14XJK	6	0.00	NAU5038	2.54	3.46	0.59	4		
	CCRI45	15HNA	6	0.00	NAU5038	3.27	4.41	0.88	4		
qFS-C7-1	CCRI45	11XJA	7	38.10	HAU1367	2.71	3.69	0.28	1		
qFS-C7-2	CCRI45	10HNA	7	53.19	PGML0193 0	3.21	4.34	0.63	3		
	CCRI45	11XJA	7	53.19	PGML0193 0	5.84	7.78	0.82	3		
	CCRI45	11HNA	7	53.19	PGML0193 0	16.07	19.93	1.38	3		
qFS-C7-3	CCRI45	09HNA	7	77.11	NAU1048	4.36	5.85	0.65	4		Sun et al., 2012; Li et al., 2019b; Jamshed et al., 2016; Deng et al., 2019
	CCRI45	11XJA	7	77.11	NAU1048	3.46	5.11	0.57	4		
	CCRI45	11HNA	7	77.11	NAU1048	4.57	6.68	0.68	4		
	CCRI45	15HNA	7	77.11	NAU1048	3.17	4.28	0.61	4		
qFS-C7-4	CCRI45	11HNA	7	92.24	NAU1085	6.13	8.34	1.23	3	BI-QTL	Sun et al., 2012; Song et al., 2017; Li et al., 2019b; Jamshed et al., 2016
	CCRI45	14XJA	7	92.24	NAU1085	3.72	5.17	0.84	3		
	CCRI45	14HNZ	7	92.24	NAU1085	4.83	6.59	1.10	3		
qFS-C9-1	CCRI45	09HNA	9	203.99	NAU2354	2.94	4.03	0.68	7		Ning et al., 2014; Wang et al., 2015
	CCRI45	11XJA	9	203.99	NAU2354	9.89	12.92	0.86	7		
	CCRI45	14XJA	9	203.99	NAU2354	10.32	13.40	0.80	7		
	CCRI45	14XJK	9	203.99	NAU2354	6.66	8.88	0.85	7		
	CCRI45	14HNZ	9	203.99	NAU2354	4.55	6.16	0.63	7		
	CCRI45	15HNA	9	203.99	NAU2354	4.28	5.73	0.90	7		
	CCRI45	15HNZ	9	203.99	NAU2354	3.14	4.29	0.58	7		
qFS-C10-1	CCRI45	11XJA	10	91.66	DPL0468	3.42	4.64	0.55	1		Li et al., 2019b
qFS-C10-2	CCRI45	14XJA	10	157.72	PGML0069 6b	2.77	3.76	-0.62	1		Zhai et al., 2016
qFS-C10-3	CCRI45	10HNA	10	178.11	BNL3563	3.23	4.38	0.68	3		
	CCRI45	11XJA	10	178.11	BNL3563	8.23	10.79	1.03	3		
	CCRI45	11HNA	10	178.11	BNL3563	18.84	22.94	1.58	3		
qFS-C11-3	CCRI45	09HNA	11	124.65	Gh074b	4.88	6.53	0.59	2		
	CCRI45	11XJA	11	124.65	Gh074b	4.01	5.41	0.38	2		
qFS-C11-5	CCRI45	11HNA	11	146.91	DPL0528	3.54	4.78	-0.74	1		Jamshed et al., 2016
qFS-C11-6	CCRI45	09HNA	11	164.92	HAU2836b	2.55	3.47	0.88	4		Shi et al., 2019; Jamshed et al., 2016
	CCRI45	11XJA	11	164.92	HAU2836b	2.60	3.55	0.62	4		
	CCRI45	11HNA	11	164.92	HAU2836b	2.80	3.80	0.68	4		
	CCRI45	14XJK	11	164.92	HAU2836b	2.82	3.83	0.77	4		
qFS-C12-1	CCRI45	09HNA	12	84.57	HAU1361	3.68	5.46	0.65	5		Wang et al., 2012; Shi et al., 2019
	CCRI45	11XJA	12	84.57	HAU1361	4.26	6.21	1.08	5		
	CCRI45	11HNA	12	84.57	HAU1361	6.15	8.72	0.90	5		
	CCRI45	14XJA	12	84.57	HAU1361	4.33	5.81	0.51	5		
	CCRI45	14HNZ	12	84.57	HAU1361	3.66	4.94	0.47	5		
qFS-C12-2	CCRI45	09HNA	12	110.86	NAU4889	4.58	6.12	1.36	4		
	CCRI45	11XJA	12	110.86	NAU4889	3.37	4.56	1.07	4		
	CCRI45	11HNA	12	110.86	NAU4889	3.75	5.08	0.79	4		
	CCRI45	15HNA	12	110.86	NAU4889	9.47	12.28	1.30	4		

qFS-C12-3	CCRI45	09HNA	12	137.96	DPL0343	4.66	7.35	-0.75	2		
	CCRI45	14XJK	12	137.96	DPL0343	2.78	4.07	-0.67	2		
qFS-C13-1	CCRI45	11XJA	13	8.68	BNL1707	2.53	3.44	0.62	2		
	CCRI45	11HNA	13	8.68	BNL1707	3.90	5.25	0.80	2		
qFS-C13-2	CCRI45	14XJK	13	32.18	NAU2730	4.81	6.72	-2.33	1		
qFS-C13-3	CCRI45	11XJA	13	164.25	CGR5242	4.10	5.53	0.83	2		
	CCRI45	11HNA	13	164.25	CGR5242	3.49	4.42	1.68	2		
qFS-C14-1	CCRI45	09HNA	14	40.73	NAU3648	3.77	5.08	0.75	6		Zhai et al., 2016
	CCRI45	11XJA	14	40.73	NAU3648	12.19	15.55	1.43	6		
	CCRI45	11HNA	14	40.73	NAU3648	2.73	3.69	1.09	6		
	CCRI45	14XJA	14	40.73	NAU3648	4.03	5.42	1.20	6		
	CCRI45	15HNA	14	40.73	NAU3648	3.15	4.25	0.87	6		
	CCRI45	15HNZ	14	40.73	NAU3648	5.57	7.41	1.04	6		
qFS-C14-2	CCRI45	09HNA	14	113.15	CGR6383	3.02	4.16	1.46	1		
qFS-C14-3	CCRI45	11XJA	14	171.34	NAU3225	4.92	6.60	0.70	1		
qFS-C14-4	CCRI45	09HNA	14	206.88	HAU1219a	2.58	3.51	-0.77	3		
	CCRI45	11HNA	14	206.88	HAU1219a	3.22	4.35	-1.16	3		
	CCRI45	15HNZ	14	206.88	HAU1219a	2.51	3.40	-0.84	3		
qFS-C15-1	CCRI45	09HNA	15	19.51	HAU1045	5.17	7.24	1.08	4		
	CCRI45	11XJA	15	19.51	HAU1045	2.95	4.21	0.99	4		
	CCRI45	14XJA	15	19.51	HAU1045	2.81	3.66	0.59	4		
	CCRI45	14XJK	15	19.51	HAU1045	3.41	4.62	0.51	4		
qFS-C15-2	CCRI45	09HNA	15	26.90	HAU1058a	6.72	8.93	1.03	5		Chen et al., 2018
	CCRI45	11XJA	15	26.90	HAU1058a	3.09	4.17	0.88	5		
	CCRI45	14XJA	15	26.90	HAU1058a	3.02	4.10	0.84	5		
	CCRI45	14XJK	15	26.90	HAU1058a	4.82	6.42	0.74	5		
	CCRI45	15HNA	15	26.90	HAU1058a	4.71	6.33	0.67	5		
qFS-C15-3	CCRI45	09HNA	15	43.61	NAU3177	4.53	6.05	1.39	4	BI-QTL	Li et al., 2019b
	CCRI45	11XJA	15	43.61	NAU3177	4.87	6.20	1.55	4		
	CCRI45	11HNA	15	43.61	NAU3177	4.21	5.66	1.23	4		
	CCRI45	15HNA	15	43.61	NAU3177	4.38	5.99	1.45	4		
qFS-C15-4	CCRI45	09HNA	15	55.21	NAU3714	2.91	3.94	1.05	4		
	CCRI45	11XJA	15	55.21	NAU3714	3.54	4.78	1.40	4		
	CCRI45	14XJA	15	55.21	NAU3714	3.01	4.09	0.91	4		
	CCRI45	14XJK	15	55.21	NAU3714	6.12	8.12	1.17	4		
qFS-C15-6	CCRI45	11HNA	15	177.43	MUSS085	5.93	7.88	-0.95	1		Li et al., 2019b
qFS-C16-1	CCRI45	09HNA	16	16.79	CGR5149	3.57	4.82	0.76	5		
	CCRI45	11XJA	16	16.79	CGR5149	2.66	3.61	0.79	5		
	CCRI45	14XJA	16	16.79	CGR5149	2.70	3.65	0.65	5		
	CCRI45	14XJK	16	16.79	CGR5149	6.15	8.17	0.84	5		
	CCRI45	15HNZ	16	16.79	CGR5149	6.35	8.40	0.77	5		
qFS-C16-3	CCRI45	11XJA	16	65.97	BNL2634	10.37	13.37	1.24	3	BI-QTL	Shen et al., 2005; Li et al., 2019b
	CCRI45	11HNA	16	65.97	BNL2634	3.09	4.18	1.03	3		
	CCRI45	15HNA	16	65.97	BNL2634	2.81	3.82	0.63	3		
qFS-C17-1	CCRI45	11XJA	17	47.26	NAU2909	2.93	3.99	0.68	3		Ning et al., 2014
	CCRI45	11HNA	17	47.26	NAU2909	8.04	10.52	1.16	3		
	CCRI45	15HNA	17	47.26	NAU2909	3.90	5.23	1.22	3		
qFS-C17-2	CCRI45	11HNA	17	56.36	NAU2325	5.12	6.83	-0.94	1		
qFS-C17-3	CCRI45	11XJA	17	122.79	HAU0195a	2.73	4.42	0.29	2	BI-QTL	Li et al., 2019b
	CCRI45	14XJA	17	122.79	HAU0195a	2.80	4.51	0.32	2		
qFS-C19-1	CCRI45	10HNA	19	17.39	NAU3405	2.62	3.56	0.65	3		
	CCRI45	11XJA	19	17.39	NAU3405	6.80	9.00	0.99	3		
	CCRI45	11HNA	19	17.39	NAU3405	15.48	19.27	1.53	3		
qFS-C19-2	CCRI45	11HNA	19	161.66	DPL0215	2.52	3.40	-1.80	1		Guo et al., 2015
qFS-C20-3	CCRI45	09HNA	20	139.90	HAU1491a	4.66	6.38	0.55	7		

	CCRI45	11XJA	20	139.90	HAU1491a	4.25	5.71	0.52	7		
	CCRI45	11HNA	20	139.90	HAU1491a	2.63	3.55	0.61	7		
	CCRI45	14XJA	20	139.90	HAU1491a	3.34	4.51	0.63	7		
	CCRI45	14HNZ	20	139.90	HAU1491a	2.93	3.95	0.29	7		
	CCRI45	15HNA	20	139.90	HAU1491a	5.72	6.90	0.63	7		
	CCRI45	15HNZ	20	139.90	HAU1491a	2.51	3.41	0.40	7		
qFS-C21-1	CCRI45	11HNA	21	57.89	CGR5217	2.91	3.95	-0.79	1		Shao et al., 2014; Shi et al., 2019
qFS-C21-2	CCRI45	09HNA	21	209.88	CIR068	2.55	3.47	0.67	2		Guo et al., 2015
	CCRI45	14XJA	21	209.88	CIR068	2.91	3.95	0.46	2		
qFS-C21-4	CCRI45	09HNA	21	238.32	PGML00972	2.79	3.78	1.08	2		Wang et al., 2016; Shi et al., 2019
	CCRI45	11HNA	21	238.32	PGML00972	3.73	5.00	2.19	2		
qFS-C21-5	CCRI45	11HNA	21	241.43	Gh132	4.27	5.51	1.87	2		
	CCRI45	15HNA	21	241.43	Gh132	3.03	4.09	1.11	2		
qFS-C22-1	CCRI45	09HNA	22	34.13	NAU3824b	3.78	5.09	0.94	5		Ning et al., 2014; Yang et al., 2015
	CCRI45	11XJA	22	34.13	NAU3824b	3.73	5.01	1.23	5		
	CCRI45	14XJA	22	34.13	NAU3824b	3.63	4.89	1.10	5		
	CCRI45	14XJK	22	34.13	NAU3824b	5.36	7.17	0.97	5		
	CCRI45	15HNA	22	34.13	NAU3824b	5.15	6.87	0.84	5		
qFS-C22-2	CCRI45	09HNA	22	108.24	CIR224a	5.05	6.75	0.86	6		
	CCRI45	11XJA	22	108.24	CIR224a	3.69	4.98	0.96	6		
	CCRI45	11HNA	22	108.24	CIR224a	2.61	3.55	0.72	6		
	CCRI45	14XJA	22	108.24	CIR224a	2.90	3.92	1.12	6		
	CCRI45	14XJK	22	108.24	CIR224a	4.37	5.88	0.91	6		
	CCRI45	15HNA	22	108.24	CIR224a	3.30	4.46	1.09	6		
qFS-C22-3	CCRI45	10HNA	22	152.64	NAU1325	2.79	3.79	0.63	3		
	CCRI45	11XJA	22	152.64	NAU1325	7.24	9.55	0.97	3		
	CCRI45	11HNA	22	152.64	NAU1325	17.15	21.12	1.52	3		
qFS-C23-1	CCRI45	11XJA	23	23.95	MUSB0442	2.53	3.58	0.96	4		
	CCRI45	11HNA	23	23.95	MUSB0442	3.63	4.89	0.77	4		
	CCRI45	14XJA	23	23.95	MUSB0442	7.39	9.74	0.70	4		
	CCRI45	15HNA	23	23.95	MUSB0442	4.22	5.66	0.49	4		
qFS-C24-1	CCRI45	09HNA	24	93.44	NAU2914	3.55	4.94	0.78	4		Zhang et al., 2012; Huang et al., 2018; Li et al., 2019
	CCRI45	11XJA	24	93.44	NAU2914	5.43	7.34	0.73	4		
	CCRI45	14XJA	24	93.44	NAU2914	5.59	7.57	1.16	4		
	CCRI45	14XJK	24	93.44	NAU2914	6.54	8.86	0.89	4		
qFS-C24-2	CCRI45	09HNA	24	108.73	PGML03834	2.67	3.63	0.65	6		
	CCRI45	11XJA	24	108.73	PGML03834	4.85	6.49	1.04	6		
	CCRI45	14XJA	24	108.73	PGML03834	3.21	4.35	0.63	6		
	CCRI45	14XJK	24	108.73	PGML03834	6.74	8.92	0.86	6		
	CCRI45	14HNZ	24	108.73	PGML03834	2.94	3.98	0.89	6		
	CCRI45	15HNA	24	108.73	PGML03834	3.60	4.86	0.58	6		
qFS-C25-1	CCRI45	14XJK	25	4.94	BNL0827	3.04	4.14	0.41	1		Shao et al., 2014
qFS-C25-2	CCRI45	14HNZ	25	29.03	CGR5201a	3.07	4.31	1.29	1		Sun et al., 2012; Li et al., 2019b
qFS-C25-4	CCRI45	11XJA	25	132.06	NAU2397	3.22	4.36	0.61	3		
	CCRI45	11HNA	25	132.06	NAU2397	3.30	4.46	0.65	3		
	CCRI45	14HNZ	25	132.06	NAU2397	2.84	3.85	0.61	3		
qFS-C26-1	CCRI45	09HNA	26	0.00	NAU1100	11.92	15.24	1.00	7		
	CCRI45	11XJA	26	0.00	NAU1100	7.41	9.74	0.94	7		
	CCRI45	14XJA	26	0.00	NAU1100	9.97	12.88	1.30	7		
	CCRI45	14XJK	26	0.00	NAU1100	2.62	3.56	0.51	7		
	CCRI45	14HNZ	26	0.00	NAU1100	2.57	3.48	0.74	7		
	CCRI45	15HNA	26	0.00	NAU1100	2.71	3.67	0.57	7		
	CCRI45	15HNZ	26	0.00	NAU1100	7.22	9.50	0.71	7		
qFS-C26-2	CCRI45	09HNA	26	4.62	NAU2920	6.48	8.57	1.09	4		
	CCRI45	11XJA	26	4.62	NAU2920	6.09	8.08	0.88	4		
	CCRI45	14XJA	26	4.62	NAU2920	9.05	11.80	0.90	4		

	CCRI45	14XJK	26	4.62	NAU2920	5.99	7.95	0.67	4		

Env., Environment; No. of EE, No. of environments of QTL expression; BI-QTL, background-independent QTL
