# Supplementary material for: Examining two sets of introgression lines across multiple environments reveals background-independent and stably expressed quantitative trait loci of fiber quality in cotton
Source: Theor Appl Genet. 2020 Mar 17;133(7):2075–93. doi: 10.1007/s00122-020-03578-0 (PMC7311500; doi:10.1007/s00122-020-03578-0)
Supplement: Supplementary file 7 — Table S4 Details of QTL clusters for fiber quality in CSSL populations. (PDF 171 kb) [file 122_2020_3578_MOESM7_ESM.pdf]

Table S4 Details of QTL clusters for fiber-quality traits in CSSL populations

Cluster name	QTL	Backgound	Env.	Chr	Position	Nearest marker	LOD	PVE(%)	Add	Note	previous reports
BISER-C2-1	qFL-C2-6	CCRI36	11LNL	2	178.82	NAU2277	5.20	5.68	0.56	BI-QTL	
	qFL-C2-6	CCRI45	10HNA	2	178.82	NAU2277	4.42	5.92	0.71	BI-QTL	
		CCRI45	11XJA	2	178.82	NAU2277	4.56	6.13	0.71		
		CCRI45	11HNA	2	178.82	NAU2277	2.56	3.48	0.56		
		CCRI45	14HNZ	2	178.82	NAU2277	3.19	4.32	0.51		
	qFS-C2-2	CCRI45	11XJA	2	178.82	NAU2277	3.97	5.36	0.75		
		CCRI45	11HNA	2	178.82	NAU2277	4.57	6.12	0.84		
		CCRI45	15HNA	2	178.82	NAU2277	2.66	3.60	0.96		
BISER-C7-1	qFL-C7-3	CCRI36	10HNA	7	92.24	NAU1085	3.62	3.98	1.13	BI-QTL	Sun et al., 2012; Song et al., 2017; Li et al., 2019b; Jamshed et al., 2016; Deng et al., 2019
		CCRI36	11HNA	7	92.24	NAU1085	3.93	4.32	1.01		
		CCRI36	11LNL	7	92.24	NAU1085	3.07	3.39	0.87		
		CCRI36	11XJS	7	92.24	NAU1085	2.63	2.91	0.80		
		CCRI36	14XJN	7	92.24	NAU1085	2.84	3.14	0.97		
		CCRI36	14XJS	7	92.24	NAU1085	2.97	3.27	0.89		
	qFL-C7-3	CCRI45	10HNA	7	92.24	NAU1085	10.65	13.64	1.34	BI-QTL	Sun et al., 2012; Song et al., 2017; Li et al., 2019b; Jamshed et al., 2016; Deng et al., 2019
		CCRI45	11HNA	7	92.24	NAU1085	7.11	9.64	1.17		
		CCRI45	14XJA	7	92.24	NAU1085	4.50	6.19	0.72		
		CCRI45	14XJK	7	92.24	NAU1085	3.43	4.78	0.81		
		CCRI45	14HNZ	7	92.24	NAU1085	8.60	11.38	1.03		
	qFM-C7-3	CCRI36	11LNL	7	92.24	NAU1085	3.41	3.76	-0.25		Sun et al., 2012
		CCRI36	11LNL	7	92.24	NAU1085	3.41	3.76	-0.25		
	qFS-C7-4	CCRI36	10HNA	7	92.24	NAU1085	3.27	3.60	1.29	BI-QTL	Sun et al., 2012; Song et al., 2017; Li et al., 2019b; Jamshed et al., 2016
		CCRI36	11HNA	7	92.24	NAU1085	3.16	3.49	1.13		
		CCRI36	11LNL	7	92.24	NAU1085	2.95	3.26	0.96		
		CCRI36	11XJS	7	92.24	NAU1085	2.88	3.19	0.95		
	qFS-C7-4	CCRI45	11HNA	7	92.24	NAU1085	6.13	8.34	1.23	BI-QTL	Sun et al., 2012; Song et al., 2017; Li et al., 2019b; Jamshed et al., 2016
		CCRI45	14XJA	7	92.24	NAU1085	3.72	5.17	0.84		
		CCRI45	14HNZ	7	92.24	NAU1085	4.83	6.59	1.10		
BISER-C15-1	qFL-C15-1	CCRI36	10HNA	15	43.61	NAU3177	3.29	3.71	0.33	BI-QTL	
		CCRI36	11HNA	15	43.61	NAU3177	4.26	5.36	0.42		
		CCRI36	11LNL	15	43.61	NAU3177	4.00	4.89	0.46		
		CCRI36	11XJS	15	43.61	NAU3177	4.43	5.18	0.41		
		CCRI36	14XJS	15	43.61	NAU3177	6.22	7.11	0.46		
	qFL-C15-1	CCRI45	09HNA	15	43.61	NAU3177	7.98	10.00	1.75	BI-QTL	
		CCRI45	10HNA	15	43.61	NAU3177	2.98	4.08	1.10		
		CCRI45	11XJA	15	43.61	NAU3177	4.09	5.44	1.05		
		CCRI45	11HNA	15	43.61	NAU3177	2.93	4.04	1.20		
		CCRI45	14XJK	15	43.61	NAU3177	3.52	4.81	1.27		
		CCRI45	14HNZ	15	43.61	NAU3177	4.63	6.15	1.32		

		CCRI45	15HNA	15	43.61	NAU3177	3.17	4.15	0.90		
		CCRI45	15HNZ	15	43.61	NAU3177	6.39	8.41	1.52		
	qFS-C15-3	CCRI36	14XJS	15	43.61	NAU3177	2.51	3.17	0.44	BI-QTL	Li et al., 2019b
	qFS-C15-3	CCRI45	09HNA	15	43.61	NAU3177	4.53	6.05	1.39	BI-QTL	Li et al., 2019b
		CCRI45	11XJA	15	43.61	NAU3177	4.87	6.20	1.55		
		CCRI45	11HNA	15	43.61	NAU3177	4.21	5.66	1.23		
		CCRI45	15HNA	15	43.61	NAU3177	4.38	5.99	1.45		
BISER-C16-1	qFL-C16-2	CCRI36	14XJN	16	65.97	BNL2634	3.39	3.86	0.94	BI-QTL	Shen et al., 2005; Li et al., 2019b
	qFL-C16-2	CCRI45	09HNA	16	65.97	BNL2634	13.43	16.95	1.24	BI-QTL	Shen et al., 2005; Li et al., 2019b
		CCRI45	10HNA	16	65.97	BNL2634	4.96	6.63	0.76		
		CCRI45	11XJA	16	65.97	BNL2634	4.25	5.71	0.78		
		CCRI45	11HNA	16	65.97	BNL2634	4.36	5.86	0.59		
		CCRI45	14XJK	16	65.97	BNL2634	4.47	5.98	0.77		
		CCRI45	14HNZ	16	65.97	BNL2634	6.33	8.38	0.84		
		CCRI45	15HNA	16	65.97	BNL2634	6.43	8.54	0.83		
		CCRI45	15HNZ	16	65.97	BNL2634	3.93	5.27	0.56		
	qFS-C16-3	CCRI36	11HNA	16	65.97	BNL2634	4.05	4.67	1.14	BI-QTL	Shen et al., 2005; Li et al., 2019b
		CCRI36	11LNL	16	65.97	BNL2634	4.03	4.61	1.00		
	qFS-C16-3	CCRI45	11XJA	16	65.97	BNL2634	10.37	13.37	1.24	BI-QTL	Shen et al., 2005; Li et al., 2019b
		CCRI45	11HNA	16	65.97	BNL2634	3.09	4.18	1.03		
		CCRI45	15HNA	16	65.97	BNL2634	2.81	3.82	0.63		
	qFL-C17-3	CCRI45	09HNA	17	47.26	NAU2909	10.78	13.85	1.18		
		CCRI45	10HNA	17	47.26	NAU2909	2.78	3.77	0.61		
		CCRI45	11XJA	17	47.26	NAU2909	2.56	3.48	0.64		
		CCRI45	11HNA	17	47.26	NAU2909	5.22	6.96	0.81		
		CCRI45	14XJK	17	47.26	NAU2909	2.65	3.60	0.49		
		CCRI45	14HNZ	17	47.26	NAU2909	5.21	6.93	0.88		
		CCRI45	15HNA	17	47.26	NAU2909	4.63	6.22	0.75		
BISER-C17-1	qFM-C17-[illegible]	CCRI36	10HNA	17	47.26	NAU2909	8.97	9.59	-0.18	BI-QTL	Zhai et al., 2016
		CCRI36	11HNA	17	47.26	NAU2909	6.50	7.04	-0.17		
		CCRI36	11LNL	17	47.26	NAU2909	9.05	9.67	-0.20		
		CCRI36	11XJS	17	47.26	NAU2909	6.46	6.99	-0.18		
		CCRI36	14XJN	17	47.26	NAU2909	8.94	9.55	-0.16		
		CCRI36	14XJS	17	47.26	NAU2909	4.52	4.95	-0.12		
	qFM-C17-[illegible]	CCRI45	15HNA	17	47.26	NAU2909	2.72	3.68	-0.18	BI-QTL	Zhai et al., 2016
		CCRI45	15HNZ	17	47.26	NAU2909	4.30	5.76	-0.20		
	qFS-C17-1	CCRI45	11XJA	17	47.26	NAU2909	2.93	3.99	0.68		Ning et al., 2014
		CCRI45	11HNA	17	47.26	NAU2909	8.04	10.52	1.16		
		CCRI45	15HNA	17	47.26	NAU2909	3.90	5.23	1.22		
BISER-C17-2	qFL-C17-5	CCRI36	11HNA	17	122.79	NAU0195[illegible]	3.08	3.42	0.36		
		CCRI36	11LNL	17	122.79	NAU0195[illegible]	3.84	4.36	0.43		
		CCRI36	11XJS	17	122.79	NAU0195[illegible]	6.91	7.72	0.56		
		CCRI36	14XJS	17	122.79	NAU0195[illegible]	6.54	7.31	0.53		
	qFM-C17-[illegible]	CCRI36	10HNA	17	122.79	NAU0195[illegible]	5.48	6.10	-0.22		Wang et al., 2016
		CCRI36	11HNA	17	122.79	NAU0195[illegible]	3.80	4.32	-0.21		
		CCRI36	11LNL	17	122.79	NAU0195[illegible]	4.82	5.27	-0.24		
		CCRI36	11XJS	17	122.79	NAU0195[illegible]	5.50	6.11	-0.24		
		CCRI36	14XJN	17	122.79	NAU0195[illegible]	7.30	8.08	-0.23		
		CCRI36	14XJS	17	122.79	NAU0195[illegible]	3.57	4.11	-0.16		
	qFS-C17-3	CCRI36	11HNA	17	122.79	NAU0195[illegible]	2.68	3.03	0.44	BI-QTL	Li et al., 2019b
		CCRI36	11LNL	17	122.79	NAU0195[illegible]	2.57	2.91	0.38		
	qFS-C17-3	CCRI45	11XJA	17	122.79	NAU0195[illegible]	2.73	4.42	0.29	BI-QTL	Li et al., 2019b
		CCRI45	14XJA	17	122.79	NAU0195[illegible]	2.80	4.51	0.32		

SER-C2-1	qFL-C2-3	CCRI45	10HNA	2	54.78	HAU1980b	10.65	13.68	1.34		Zhai et al., 2016
		CCRI45	11XJA	2	54.78	HAU1980b	11.96	15.33	1.40		
		CCRI45	11HNA	2	54.78	HAU1980b	7.11	9.67	1.17		
		CCRI45	14XJA	2	54.78	HAU1980b	4.50	6.21	0.72		
		CCRI45	14XJK	2	54.78	HAU1980b	3.43	4.80	0.81		
		CCRI45	14HNZ	2	54.78	HAU1980b	8.60	11.40	1.03		
		CCRI45	15HNA	2	54.78	HAU1980b	6.42	8.66	1.16		
		CCRI45	15HNZ	2	54.78	HAU1980b	7.53	9.81	0.95		
	qFS-C2-1	CCRI45	09HNA	2	54.78	HAU1980b	2.66	3.61	0.85		
		CCRI45	11XJA	2	54.78	HAU1980b	7.59	9.88	1.27		
		CCRI45	11HNA	2	54.78	HAU1980b	6.13	8.37	1.23		
		CCRI45	14XJA	2	54.78	HAU1980b	3.72	5.18	0.84		
		CCRI45	14HNZ	2	54.78	HAU1980b	4.83	6.61	1.10		
		CCRI45	15HNA	2	54.78	HAU1980b	6.35	8.50	1.83		
		CCRI45	15HNZ	2	54.78	HAU1980b	2.56	3.47	0.68		
SER-C7-1	qFL-C7-2	CCRI45	11XJA	7	53.19	PGML01950	4.27	5.75	0.62		
		CCRI45	11HNA	7	53.19	PGML01950	6.81	8.99	0.82		
		CCRI45	14XJK	7	53.19	PGML01950	2.98	4.03	0.54		
	qFS-C7-2	CCRI45	10HNA	7	53.19	PGML01950	3.21	4.34	0.63		
		CCRI45	11XJA	7	53.19	PGML01950	5.84	7.78	0.82		
		CCRI45	11HNA	7	53.19	PGML01950	16.07	19.93	1.38		
SER-C10-1	qFL-C10-2	CCRI45	11XJA	10	178.11	BNL3563	5.48	7.32	0.75		Shi et al., 2019
		CCRI45	11HNA	10	178.11	BNL3563	7.96	10.42	0.95		
		CCRI45	14XJK	10	178.11	BNL3563	3.02	4.09	0.58		
	qFS-C10-3	CCRI45	10HNA	10	178.11	BNL3563	3.23	4.38	0.68		
		CCRI45	11XJA	10	178.11	BNL3563	8.23	10.79	1.03		
		CCRI45	11HNA	10	178.11	BNL3563	18.84	22.94	1.58		
SER-C12-1	qFL-C12-3	CCRI45	11XJA	12	84.57	HAU1361	3.23	4.40	0.57		
		CCRI45	15HNZ	12	84.57	HAU1361	3.17	4.28	0.31		
	qFS-C12-1	CCRI45	09HNA	12	84.57	HAU1361	3.68	5.46	0.65		Wang et al., 2012; Shi et al., 2019
		CCRI45	11XJA	12	84.57	HAU1361	4.26	6.21	1.08		
		CCRI45	11HNA	12	84.57	HAU1361	6.15	8.72	0.90		
		CCRI45	14XJA	12	84.57	HAU1361	4.33	5.81	0.51		
		CCRI45	14HNZ	12	84.57	HAU1361	3.66	4.94	0.47		
SER-C12-2	qFL-C12-4	CCRI45	09HNA	12	110.86	NAU4889	12.12	15.42	1.29		
		CCRI45	10HNA	12	110.86	NAU4889	3.57	4.81	0.71		
		CCRI45	11XJA	12	110.86	NAU4889	5.99	7.95	0.89		
		CCRI45	11HNA	12	110.86	NAU4889	3.41	4.61	0.57		
		CCRI45	14XJK	12	110.86	NAU4889	3.17	4.28	0.55		
		CCRI45	14HNZ	12	110.86	NAU4889	5.54	7.40	0.85		
		CCRI45	15HNZ	12	110.86	NAU4889	3.61	4.87	0.78		
	qFS-C12-2	CCRI45	09HNA	12	110.86	NAU4889	4.58	6.12	1.36		
		CCRI45	11XJA	12	110.86	NAU4889	3.37	4.56	1.07		
		CCRI45	11HNA	12	110.86	NAU4889	3.75	5.08	0.79		
		CCRI45	15HNA	12	110.86	NAU4889	9.47	12.28	1.30		
SER-C13-1	qFL-C13-1	CCRI45	10HNA	13	8.68	BNL1707	3.07	4.17	0.60		
		CCRI45	11XJA	13	8.68	BNL1707	2.76	3.75	0.58		
	qFS-C13-1	CCRI45	11XJA	13	8.68	BNL1707	2.53	3.44	0.62		
		CCRI45	11HNA	13	8.68	BNL1707	3.90	5.25	0.80		
SER-C13-2	qFL-C13-3	CCRI45	10HNA	13	164.25	CGR5242	2.74	3.71	0.61		
		CCRI45	11XJA	13	164.25	CGR5242	3.26	4.40	0.68		
		CCRI45	11HNA	13	164.25	CGR5242	5.36	7.14	0.88		

		CCRI45	14XJK	13	164.25	CGR5242	4.37	5.88	0.75		
	qFS-C13-3	CCRI45	11XJA	13	164.25	CGR5242	4.10	5.53	0.83		
		CCRI45	11HNA	13	164.25	CGR5242	3.49	4.42	1.68		
SER-C14-1	qFL-C14-1	CCRI45	11XJA	14	40.73	NAU3648	7.92	10.41	1.04		Zhai et al., 2016
		CCRI45	14XJA	14	40.73	NAU3648	2.81	3.81	0.51		
	qFS-C14-1	CCRI45	09HNA	14	40.73	NAU3648	3.77	5.08	0.75		Zhai et al., 2016
		CCRI45	11XJA	14	40.73	NAU3648	12.19	15.55	1.43		
		CCRI45	11HNA	14	40.73	NAU3648	2.73	3.69	1.09		
		CCRI45	14XJA	14	40.73	NAU3648	4.03	5.42	1.20		
		CCRI45	15HNA	14	40.73	NAU3648	3.15	4.25	0.87		
		CCRI45	15HNZ	14	40.73	NAU3648	5.57	7.41	1.04		
	qFL-C14-2	CCRI45	14XJK	14	46.54	NAU5421	2.68	3.64	-0.83		Zhai et al., 2016
SER-C14-2	qFL-C14-7	CCRI45	11HNA	14	206.88	HAU1219	3.48	4.70	-0.79		
		CCRI45	15HNZ	14	206.88	HAU1219	2.92	3.95	-0.58		
	qFS-C14-4	CCRI45	09HNA	14	206.88	HAU1219	2.58	3.51	-0.77		
		CCRI45	11HNA	14	206.88	HAU1219	3.22	4.35	-1.16		
		CCRI45	15HNZ	14	206.88	HAU1219	2.51	3.40	-0.84		
SER-C15-1	qFM-C15-1	CCRI45	10HNA	15	26.90	HAU1058a	2.91	3.99	0.19		Chen et al., 2018; Wang et al., 2016
		CCRI45	11HNA	15	26.90	HAU1058	2.62	3.59	0.15		
	qFS-C15-2	CCRI45	09HNA	15	26.90	HAU1058	6.72	8.93	1.03		Chen et al., 2018
		CCRI45	11XJA	15	26.90	HAU1058	3.09	4.17	0.88		
		CCRI45	14XJA	15	26.90	HAU1058	3.02	4.10	0.84		
		CCRI45	14XJK	15	26.90	HAU1058	4.82	6.42	0.74		
		CCRI45	15HNA	15	26.90	HAU1058	4.71	6.33	0.67		
SER-C16-1	qFM-C16-1	CCRI45	09HNA	16	16.79	CGR5149	2.52	3.43	0.24		
		CCRI45	10HNA	16	16.79	CGR5149	3.34	4.52	0.20		
	qFS-C16-1	CCRI45	09HNA	16	16.79	CGR5149	3.57	4.82	0.76		
		CCRI45	11XJA	16	16.79	CGR5149	2.66	3.61	0.79		
		CCRI45	14XJA	16	16.79	CGR5149	2.70	3.65	0.65		
		CCRI45	14XJK	16	16.79	CGR5149	6.15	8.17	0.84		
		CCRI45	15HNZ	16	16.79	CGR5149	6.35	8.40	0.77		
SER-C16-2	qFL-C16-3	CCRI36	10HNA	16	176.71	BNL3065	3.10	3.50	-0.63		Shi et al., 2019
		CCRI36	11HNA	16	176.71	BNL3065	2.75	3.10	-0.70		
		CCRI36	11LNL	16	176.71	BNL3065	3.05	3.44	-0.64		
	qFS-C16-4	CCRI36	10HNA	16	176.71	BNL3065	3.81	4.21	-0.99		Shi et al., 2019
		CCRI36	11HNA	16	176.71	BNL3065	3.02	3.39	-0.69		
		CCRI36	11LNL	16	176.71	BNL3065	3.32	3.73	-0.82		
		CCRI36	11XJS	16	176.71	BNL3065	3.28	3.69	-0.72		
SER-C19-1	qFL-C19-1	CCRI45	11XJA	19	17.39	NAU3405	4.28	5.76	0.70		Wang et al., 2017a
		CCRI45	11HNA	19	17.39	NAU3405	7.37	9.70	0.96		
		CCRI45	14XJK	19	17.39	NAU3405	2.51	3.41	0.56		
	qFS-C19-1	CCRI45	10HNA	19	17.39	NAU3405	2.62	3.56	0.65		
		CCRI45	11XJA	19	17.39	NAU3405	6.80	9.00	0.99		
		CCRI45	11HNA	19	17.39	NAU3405	15.48	19.27	1.53		
SER-C20-1	qFL-C20-3	CCRI45	11XJA	20	139.90	HAU1491	7.79	10.25	0.59		
		CCRI45	14XJA	20	139.90	HAU1491	3.66	4.93	0.33		
		CCRI45	14XJK	20	139.90	HAU1491	3.78	5.07	0.35		
		CCRI45	15HNZ	20	139.90	HAU1491	3.94	5.30	0.44		
	qFS-C20-3	CCRI45	09HNA	20	139.90	HAU1491	4.66	6.38	0.55		
		CCRI45	11XJA	20	139.90	HAU1491	4.25	5.71	0.52		
		CCRI45	11HNA	20	139.90	HAU1491	2.63	3.55	0.61		
		CCRI45	14XJA	20	139.90	HAU1491	3.34	4.51	0.63		
		CCRI45	14HNZ	20	139.90	HAU1491	2.93	3.95	0.29		
		CCRI45	15HNA	20	139.90	HAU1491	5.72	6.90	0.63		
		CCRI45	15HNZ	20	139.90	HAU1491	2.51	3.41	0.40		
SER-C21-1	qFL-C21-4	CCRI45	10HNA	21	238.32	PGML00972	3.31	4.31	1.80		

		CCRI45	11XJA	21	238.32	PGML00972	2.65	3.60	0.56		
		CCRI45	11HNA	21	238.32	PGML00972	7.61	9.99	1.15		
		CCRI45	14HNZ	21	238.32	PGML00972	3.21	4.34	0.73		
		CCRI45	15HNZ	21	238.32	PGML00972	2.95	4.01	0.69		
	qFS-C21-4	CCRI45	09HNA	21	238.32	PGML00972	2.79	3.78	1.08		Wang et al., 2016; Shi et al., 2019
		CCRI45	11HNA	21	238.32	PGML00972	3.73	5.00	2.19		
SER-C21-2	qFL-C21-5	CCRI45	10HNA	21	241.43	Gh132	3.32	4.53	1.43		
		CCRI45	11XJA	21	241.43	Gh132	2.70	3.66	0.62		
		CCRI45	11HNA	21	241.43	Gh132	2.50	3.40	0.49		
		CCRI45	14XJK	21	241.43	Gh132	3.42	4.59	1.46		
		CCRI45	14HNZ	21	241.43	Gh132	4.31	5.77	1.38		
		CCRI45	14HNZ	21	241.43	Gh132	5.64	7.20	1.90		
		CCRI45	15HNA	21	241.43	Gh132	3.45	4.64	0.74		
		CCRI45	15HNZ	21	241.43	Gh132	3.47	4.67	0.57		
	qFS-C21-5	CCRI45	11HNA	21	241.43	Gh132	4.27	5.51	1.87		
		CCRI45	15HNA	21	241.43	Gh132	3.03	4.09	1.11		
SER-C22-1	qFL-C22-3	CCRI45	11XJA	22	152.64	NAU1325	7.55	9.91	0.92		
		CCRI45	11HNA	22	152.64	NAU1325	2.58	3.51	0.54		
		CCRI45	14XJK	22	152.64	NAU1325	4.44	5.98	0.68		
	qFS-C22-3	CCRI45	10HNA	22	152.64	NAU1325	2.79	3.79	0.63		
		CCRI45	11XJA	22	152.64	NAU1325	7.24	9.55	0.97		
		CCRI45	11HNA	22	152.64	NAU1325	17.15	21.12	1.52		

Env. Environment; BI-QTL, background-independent QTL
